# High-resolution strain-level microbiome composition analysis from short reads

**DOI:** 10.1186/s40168-023-01615-w

**Published:** 2023-08-17

**Authors:** Herui Liao, Yongxin Ji, Yanni Sun

**Affiliations:** Department of Electrical Engineering, City University of Hong Kong, Kowloon, China

**Keywords:** Strain composition analysis, Metagenomic data, *k*-mers indexing structure

## Abstract

**Background:**

Bacterial strains under the same species can exhibit different biological properties, making strain-level composition analysis an important step in understanding the dynamics of microbial communities. Metagenomic sequencing has become the major means for probing the microbial composition in host-associated or environmental samples. Although there are a plethora of composition analysis tools, they are not optimized to address the challenges in strain-level analysis: highly similar strain genomes and the presence of multiple strains under one species in a sample. Thus, this work aims to provide a high-resolution and more accurate strain-level analysis tool for short reads.

**Results:**

In this work, we present a new strain-level composition analysis tool named StrainScan that employs a novel tree-based *k*-mers indexing structure to strike a balance between the strain identification accuracy and the computational complexity. We tested StrainScan extensively on a large number of simulated and real sequencing data and benchmarked StrainScan with popular strain-level analysis tools including Krakenuniq, StrainSeeker, Pathoscope2, Sigma, StrainGE, and StrainEst. The results show that StrainScan has higher accuracy and resolution than the state-of-the-art tools on strain-level composition analysis. It improves the F1 score by 20% in identifying multiple strains at the strain level.

**Conclusions:**

By using a novel *k*-mer indexing structure, StrainScan is able to provide strain-level analysis with higher resolution than existing tools, enabling it to return more informative strain composition analysis in one sample or across multiple samples. StrainScan takes short reads and a set of reference strains as input and its source codes are freely available at https://github.com/liaoherui/StrainScan.

Video Abstract

**Supplementary Information:**

The online version contains supplementary material available at 10.1186/s40168-023-01615-w.

## Background

There is accumulating evidence showing that strains within a species can have different metabolic and functional versatility due to the genomic variations [1–3]. Strains under the same species can exhibit high sequence diversity and different gene organizations [4]. Unique genes or SNPs to a strain may lead to new enzymatic functions, antibiotic resistance, virulence, different infecting viruses, etc. For example, there are at least thousands of strains identified for *E. coli*, with some of them containing virulence factors while others being commensal. A notable example is 2011 *E. coli* outbreak in Germany caused by a strain O104:H4, which acquired a Shiga toxin-encoding prophage and other virulence factors [5].

As different strains can have different biological properties, pinpointing the strains is important for both composition and functional analysis of microbiome. Metagenomic sequencing data, which contains sequenced genetic materials from a host-associated or environmental sample, has become a major source to study strain-level compositions of bacteria. There is an increasing number of studies generating new knowledge about strains’ genotypes and phenotypes in different samples. For example, Pollard et al. showed that many prevalent bacterial species have strain-level composition associated with a geographic location in 198 marine metagenomes [6]. A closely related study showed that dominant *E. coli* strains change over time in the gut microbiome of a Crohn’s disease patient [7]. *P. copri*, another very common bacterium in the human gut, has been proven to have a tight link between its strains and the host’s geographical location and dietary habits [8, 9]. Some strains of the potential probiotic *A. muciniphila* are found to have anti-inflammatory properties, which could have beneficial effects on obesity and diabetes [10]. In addition, there are differences in the distribution of strains in different parts of the human body. For example, a past study [11] has found that strains of *C. acnes* and *S. epidermidis* collected from different sites of the body are heterogeneous and multiphyletic.

**Fig. 1:**
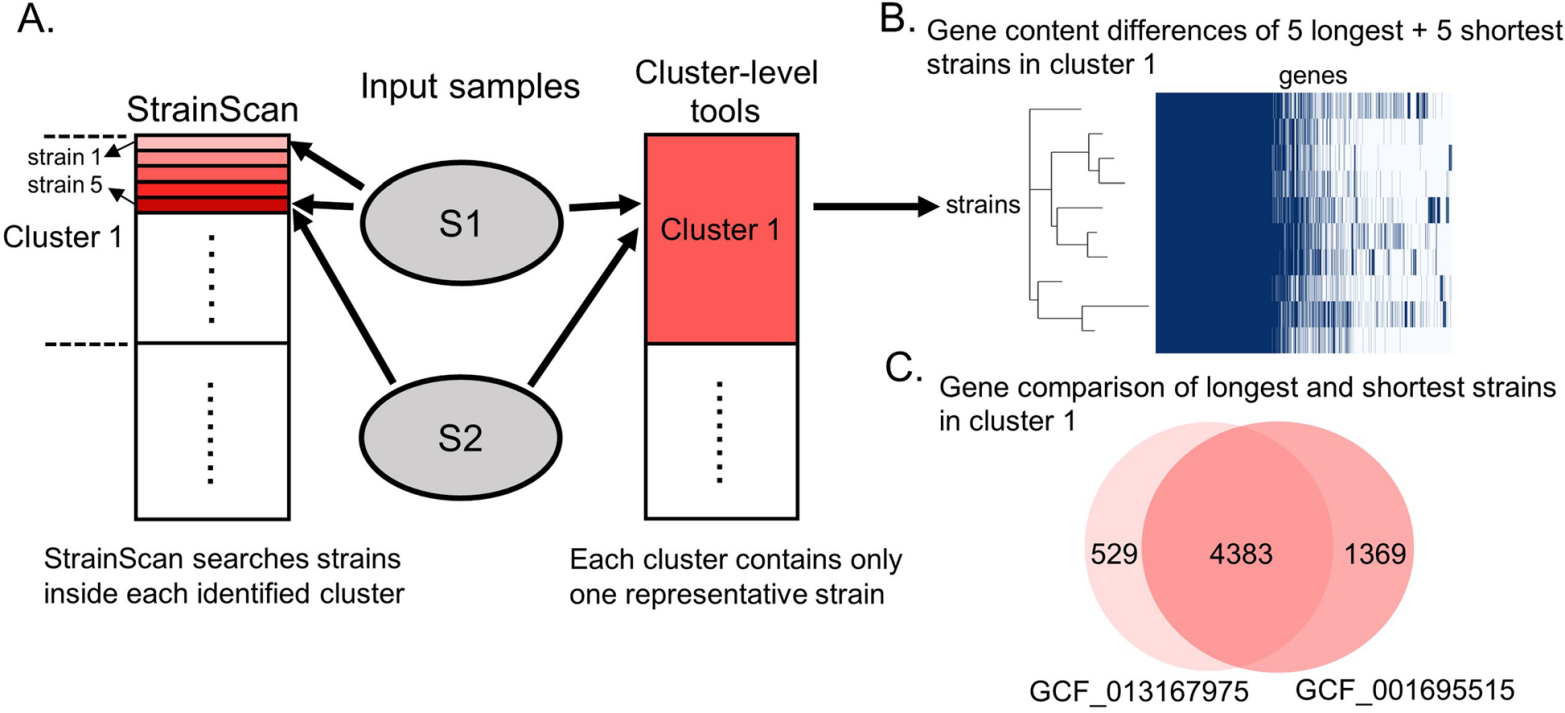
**A** StrainScan achieves higher strain-level resolution by searching strains inside identified clusters. In contrast, cluster-level tools like StrainGE and StrainEst only return the representative strain of the identified cluster and will not search for other strains in the cluster. “S1” and “S2” are two input metagenomic samples. **B–C** Gene content differences between strains from a real cluster (named cluster 1) containing 216 *E. coli* strains. The 10 strains in **B** have a total of 1722 strain-specific genes. “GCF_001695515” and “GCF_013167975” in **C** are the longest and shortest strain in cluster 1, respectively

Despite the importance of strain-level analysis, it remains difficult to conduct the taxonomic analysis below the species level. One challenge comes from the fact that multiple highly similar strains can exist simultaneously in one sample [12]. For example, one recent study [13] found that 2 or 3 Staphylococcus epidermidis strains can coexist in human fecal samples with a Mash [14] distance of approximately 0.005. Similarly, there are reports showing that multiple strains of *C. acnes*, an important component in the human skin microbiome, often form a complex mixture [15]. Some of these coexisting strains exhibit high sequence similarities, with a Mash distance of approximately 0.0004. Moreover, a study [16] analyzing 2144 human fecal metagenomes revealed that numerous samples contained highly similar strains of *Bacteroides dorei*, coexisting with one another. Commonly used metagenomic binning and assembly tools are not designed to distinguish different strains. Although there are strain analysis tools, they may either require multiple samples from the same population [17], only output the dominant strain [18–21], or pose a restriction on the similarity between the strains [22]. The second challenge that immediately follows is the resolution of strain-level identification. The resolution here is reflected by the size of the reference database, with a larger number of reference strains indicating a higher resolution [23]. Although some of the strains share very high similarities, there are no known similarity cutoffs below which the genetic differences can be ignored. For example, *E. coli* CFT073 and *E. coli* Nissle 1917, which are pathogenic and probiotic respectively, have a sequence similarity of 99.98% [24]. Similarly, one phage-host coevolution study [25] found that even if the bacterial strains contain high genome ANI (>99.9%), the strains can be infected by different phages, showing different defense or adsorption mechanisms. For some species with high strain-level diversity, even a few SNVs can lead to phenotypic variations [26, 27]. Thus, a higher resolution can enable a more accurate characterization of the relationship between genotypes and phenotypes. Tools including StrainGE [28] and StrainEst [29] are designed to untangle strain mixtures, but are limited to reporting a representative strain in a sampled strain genome database. Their clustering cutoffs (0.9 *k*-mer Jaccard similarity (StrainGE) or 99.4% ANI (StrainEst)) can still lead to large clusters for some bacteria. Although StrainGE can further identify SNPs/deletions against the identified representative strain in a sample, it doesn’t pinpoint the specific strain in the identified clusters. Two *k*-mer-based tools, Krakenuniq [30] and StrainSeeker [31], also have a very low resolution in strain-level identification when strains in the database share high similarities. The third challenge is the identification of low-abundance strains. For example, the de novo strain construction tools [32, 33], which aim to reconstruct strains by using assembly-based strategies, usually require a high coverage of strains to achieve an accurate strain reconstruction. Besides, many strain-analysis tools [34–36] also require strain coverage greater than 10X to return accurate identification. Thus, it remains a challenge to identify strains with low coverage for these tools. The last challenge is strain identification time. According to the recently published studies [23, 37], most alignment-based strain-level identification tools including Sigma [38], and Pathoscope2 [39] can be computationally expensive when the database is large. While the large reference database can increase coverage of intra-species diversity, it also requires more computational resources.

Thus, there is a pressing need to provide more sensitive, accurate, and efficient strain-level analysis for metagenomic data. In this work, we introduce StrainScan, an open-source tool that can accurately detect known strains from sequencing data, including metagenomic data or whole-genome sequencing data. In order to strike a balance between the resolution and computational complexity, we developed a novel hierarchical *k*-mers indexing structure for a large number of strains, which usually demonstrate heterogeneous similarity distributions. In the first step, we cluster highly similar strains into clusters. Then we design a novel Cluster Search Tree (CST), a tree-based indexing structure for cluster search. By carefully balancing the number of *k*-mers in each node, we optimize the CST to prevent false positive strain identification for low abundance strains. In the second step, we use strain-specific *k*-mers and *k*-mers that represent SNVs and structural variations to determine which strains are likely to present. The final output of StrainScan includes the identified strains and their abundances. By searching strains inside the identified clusters, StrainScan achieves a higher resolution than cluster-level tools such as StrainGE and StrainEst, which only keep one representative strain for each cluster. As shown in Fig. 1A, different resolutions can lead to different observations and conclusions. While StrainScan can identify two different strains in sample S1, StrainGE or StrainEst does not distinguish them because they are from the same cluster. Similarly, pinpointing a specific strain rather than a cluster when comparing two samples (S1 and S2) can lead to more accurate gene composition-based analyses because the strains in one cluster can still possess very different gene contents (Fig. 1B and C).

By benchmarking StrainScan with other available tools on multiple simulated and real sequencing datasets, we demonstrate that StrainScan can output strain-level composition with higher accuracy than the state-of-the-art tool. In particular, when compared to the state-of-the-art tools such as StrainGE, StrainScan improved the F1 score by more than 20% in identifying multiple strains at the strain level. StrainScan is a targeted strain composition analysis tool, requiring users to provide reference genomes for bacteria of interest. By supporting customized construction of the indexing structure for any set of reference genomes, it can be applied to any bacterium.

## Methods

### Overview of StrainScan

**Fig. 2:**
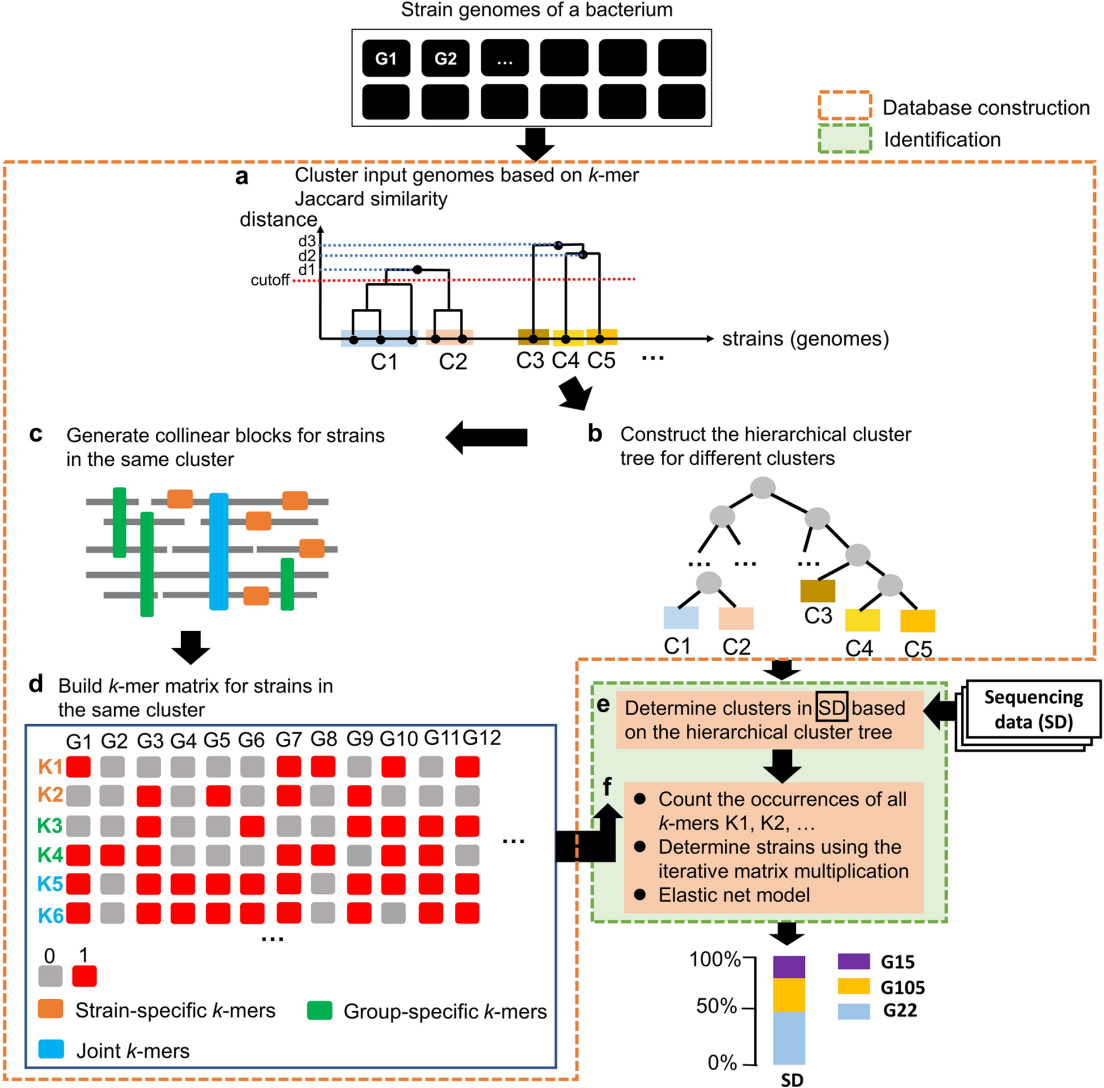
The overview of StrainScan. (a) The sketch of the strain genome clustering process. Given the strain genomes (G1, G2, ...) of the bacteria of interest, all-against-all *k*-mers Jaccard similarities are computed using Dashing [40]. Genomes are then clustered using single-linkage hierarchical clustering. By default, the clustering threshold is set to a Jaccard similarity of 0.95. In this example, given the cutoff represented by the dashed red line, five clusters from C1 to C5 are output by the clustering process. (b) Given the clusters, construct the hierarchical cluster tree for later cluster-level identification. (c) Generate collinear blocks to extract *k*-mers that can help distinguish different strains inside the same cluster. (d) Step d concludes the indexing structure process for the reference genomes. (e) and (f) The indexing structure and the sequencing data (reads) are input for strain search. (e) Search for clusters. (f) Strains are identified by the iterative matrix multiplication, and the relative abundance profile is finally inferred by elastic net regression

StrainScan is designed to identify known strains from short reads directly. Because there are many species-level composition analysis tools for metagenomic data, the inputs to StrainScan are the short reads in “fastq” format and strain genomes for targeted bacteria in “fasta” format. To strike a balance between the strain identification resolution and computational cost, we design a hierarchical indexing method that combines a fast but coarse-grained Cluster Search Tree (CST) and a slower but fine-grained strain identification strategy inside a cluster. As shown in the flowchart in Fig. 2, we first create a cluster tree-based indexing structure. With our efficient and accurate cluster search method on this tree, we can first pinpoint a cluster that is present in the sample. Then we will use carefully chosen *k*-mers to distinguish different strains in the identified clusters. The hierarchical method has several advantages. First, it allows us to accommodate the heterogeneous similarity distribution between strains with some strains sharing much higher similarities than others. The strains with low similarity can be quickly identified by our fast CST search strategy. And only those highly similar strains need a finer distinction in the second step. Second, the hierarchical method can increase the search accuracy by allowing us to use more unique *k*-mers (Supplementary Table S1). Any *k*-mers that is shared between clusters now can be utilized for within-cluster search. Third, the hierarchical method can reduce the memory footprint. Without the hierarchical method, we need to search strains from all references that contain a large number of *k*-mers. Given the clusters identified by CST search, StrainScan only needs to search strains in identified clusters that contain fewer strains and *k*-mers. For example, the total number of *k*-mers in *E. coli* reference set before clustering is 192,325,016, while the number of *k*-mers in the largest cluster after clustering is reduced to 16,071,080 (Supplementary Table S1).

**Fig. 3:**
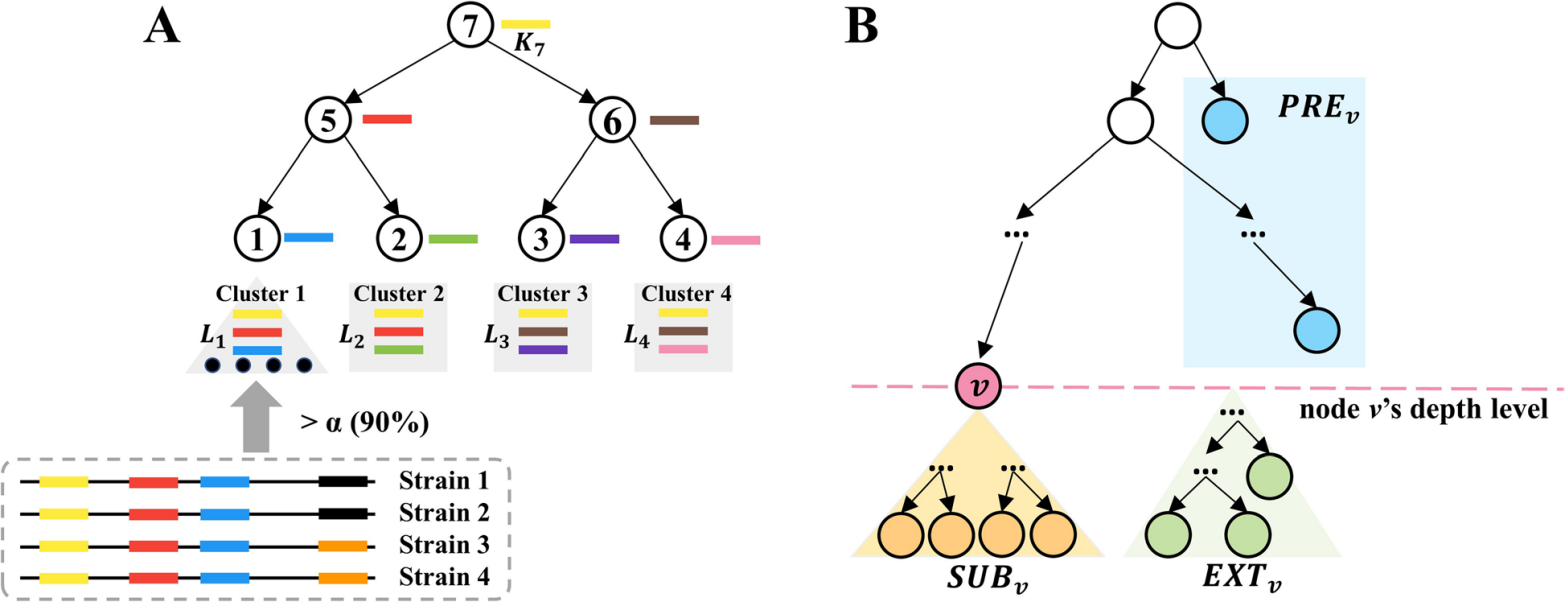
**A** An example of the *k*-mers assignment in the CST-based indexing structure. Each node possesses *k*-mers unique to its rooted subtree and is shared by most of the strains in the subtree. Each bar with a specific color represents a *k*-mers and each node is assigned with one unique *k*-mers in this example. **B** When constructing node *v*’s *k*-mers set, all leaf nodes will be divided into three groups named $PRE_v$, $SUB_v$, and $EXT_v$

### Cluster Search Tree (CST) construction

Given many strains’ genomes of the same species, we first calculated a Jaccard similarity matrix with an alignment-free, *k*-mers based method Dashing [40] ($k = 31$). Then, we performed the agglomerative hierarchical clustering (single-linkage) based on this matrix, grouping the strains into a dendrogram. Finally, we chose a fixed height cutoff *H* (0.95 by default) to cut the dendrogram into many clusters, with which consisting of one or more strains. The strains inside each cluster have the *k*-mer-based Jaccard similarity $\ge 0.95$, which roughly corresponds to average nucleotide identity (ANI) of 99.89% [28].

To pinpoint the cluster where a strain is contained, we will convert the clusters and the dendrogram into a CST to support both accurate and efficient cluster search. The CST keeps the same tree topology as the dendrogram except that each cluster is represented by a leaf node in the tree. In addition, we discard the distance information in the dendrogram so that the distance between each node and its parent (or child) is uniform, regardless of their Jaccard similarities. Thus, the CST is a full binary tree. In order to support the cluster search, each node contains a set of *k*-mers that are unique to the subtree rooted by this node. By conducting *k*-mers match, the CST will guide us to take either the left child or the right child until reaching one or multiple leaf nodes (i.e., clusters). We first describe how we assign k-mers for each node.

#### *k*-mers assignment for the nodes

A CST is defined by two elements: the tree topology and the *k*-mers set assigned for each node. In this section, we will describe how we assign *k*-mers for the nodes to support the cluster search. For a node *v* in the CST, we denote the subtree rooted by *v* as $T_v$. The *k*-mers assignment of *v* follows two criteria. First, the *k*-mers should be shared by most of the strains in the leaf nodes of $T_v$. Second, the *k*-mers are unique to the strains in $T_v$. The two criteria are visualized using an example in Fig. 3A.

Following the two criteria, we first assign leaf nodes with *k*-mers extracted from strains in their corresponding clusters. To use *k*-mers that represent relatively well-conserved features in the underlying strains, only the *k*-mers that appear in at least $\alpha \%$ of the strains will be kept for clusters with multiple strains. Big $\alpha$ indicates that only *k*-mers shared by many strains are used for building the CST while small $\alpha$ allows the CST to use strain(s)-specific *k*-mers. We compared the cluster identification performance using a range of $\alpha$ in our experiments. According to the empirical results in Supplementary Fig. S1, we set the default $\alpha$ as 90.

Hereafter, we denote the initial *k*-mers set for a leaf node *v* as $\mathbf {L_v}$. Next, starting from the leaf nodes, we recursively move the shared *k*-mers between every two sibling nodes towards their parent. In the last step, all *k*-mers that occur in more than one node will be removed. At the end of this process, each node *v* (an internal node or a leaf node) contains a set of unique *k*-mers denoted as $\mathbf {K_v}$. Specifically, $K_v$ for a node *v* can be constructed using a set operation as shown in Equation (1). For the node *v* with depth $d_v$, all the leaf nodes are divided into three groups based on their relationship with *v*, as shown in Fig. 3B and defined below.
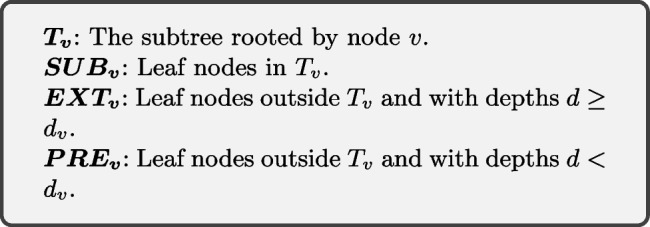

1 $$\begin{aligned} K_{v} = \bigcap \limits _{i\in SUB_{v}} L_{i} - \bigcup \limits _{i\in EXT_v} L_{i} - \bigcup \limits _{i\in PRE_v} L_{i} \end{aligned}$$

The CST constructed so far is similar to the tree built in StrainSeeker [31]. Although using the unique *k*-mers $\mathbf {K_v}$ can guide the search for identifying strain clusters, a significant limitation is that some nodes only contain a small number of unique *k*-mers, which can lead to false positive (FP) matches more likely than nodes with many *k*-mers. This was observed when applying StrainSeeker. Take the StrainSeeker database built from 112 *P. copri* strains as an example. Out of 222 nodes, 21 nodes are empty, and 104 nodes have *k*-mers fewer than 1000. The nodes with small *k*-mers sets tend to be matched by chance and thus lead to FP identification. In order to address this limitation, we will augment those nodes by adding *k*-mers that do not add ambiguity to the cluster search. The details of the CST optimization method can be found in Supplementary Section 1.1.

### Cluster search in the CST

**Fig. 4:**
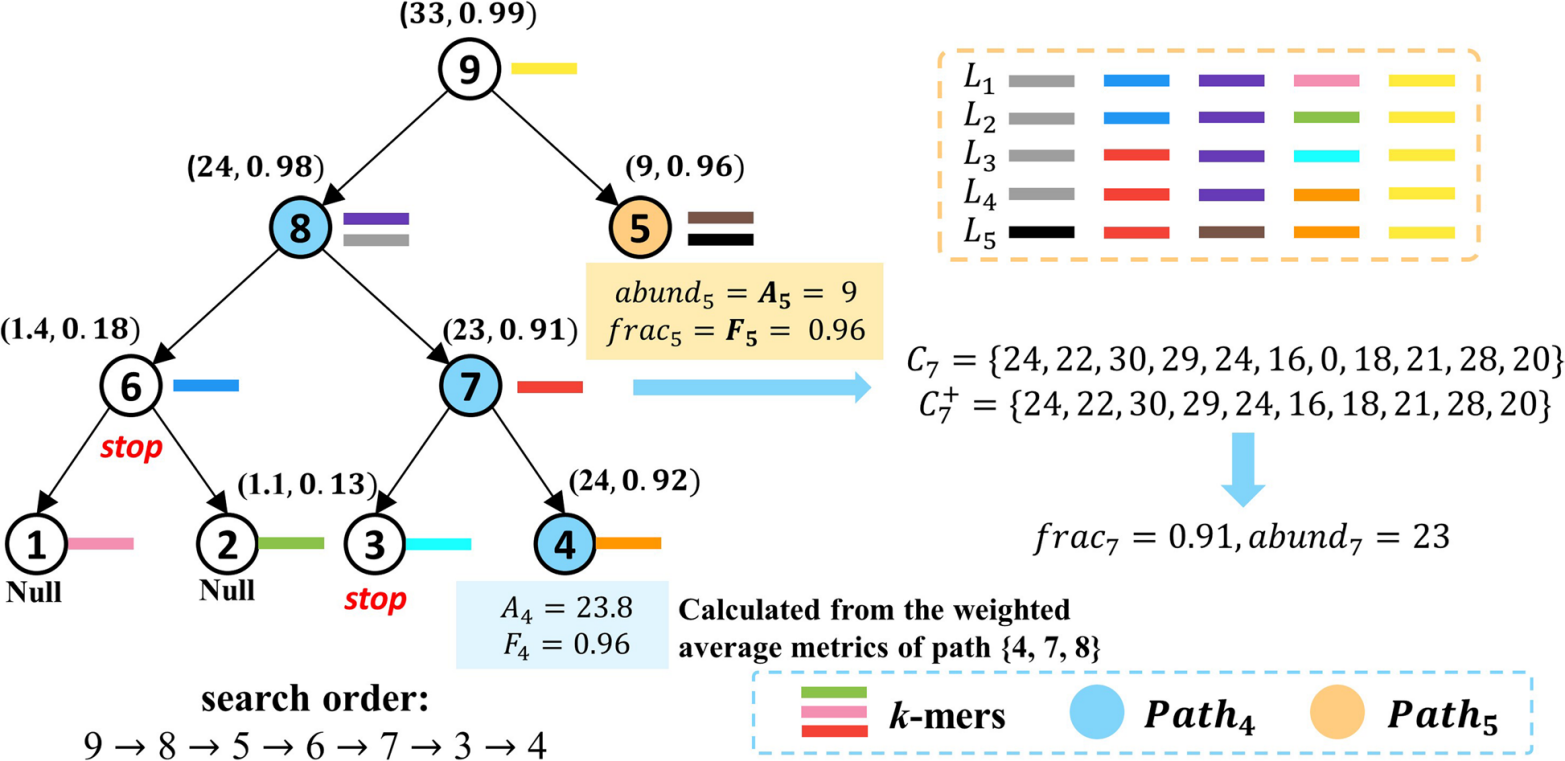
An example of the cluster search process. The values of the two scoring metrics $(abund_i, frac_i)$ are shown beside each node *v*. The search results contain clusters 4 and 5 with estimated abundance 23.8 and 9, respectively. Nodes 6 and 3 did not pass the binomial test, thereby failing to traverse their descendants. The nodes in $Path_4$ and $Path_5$ are colored by blue and orange, respectively. $K_4$ shares the same orange *k*-mers with $L_5$. Thus, we need to adjust $C_4$ based on cluster 5’s estimated abundance $A_5$ to calculate the accurate scoring metrics

Given the input sequencing data, we first extract all *k*-mers from the CST and conduct fast *k*-mers match for all short reads using Jellyfish [41]. Then, the *k*-mers match counts by all reads will be mapped back to the CST. Each node *v* will be assigned with a one-dimensional numerical vector $\mathbf {C_v} = (c_1, c_2, ..., c_{|Kv|})$, with each cell recording a *k*-mers match count. The cluster search algorithm is based on Breadth-first Search (BFS), starting from the root and examining *k*-mers matches for nodes level by level (Fig. 4). The *k*-mers match vector $C_v$ of each node *v* is used to decide whether or not to traverse *v*’s descendants based on a binomial test. The final search results contain one or multiple leaf nodes representing the strain clusters present in the sequencing data.

#### Scoring metrics

When the search visits a node *v*, two scoring metrics will be calculated to decide which child nodes will be visited. As shown in Fig. 4, the first metric is the fraction of matching *k*-mers ($\mathbf {frac_v}$), which represents the fraction of *k*-mers in $K_v$ that is present in the sequencing data. It is defined as:

2 $$\begin{aligned} frac_v = \frac{|C^+_v|}{|C_v|} \end{aligned}$$

where $C^+_v$ represents the vector which contains all positive *k*-mers counts from $C_v$.

The second metric is the average *k*-mers match count ($\mathbf {abund_v}$), which is computed only using *k*-mers with positive matching counts. And when $frac_v < 0.1$, $abund_v$ will be set to 0.

3 $$\begin{aligned} abund_v = \left\{ \begin{array}{lr} \frac{\sum _{c \in C^+_v}{c}}{|C^+_v|}, &{} {frac_v \ge 0.1} \\ 0, &{} {frac_v < 0.1} \\ \end{array} \right. \end{aligned}$$

#### The search strategy

After we calculate the two scoring metrics of *v*, we conduct a binomial test to decide the traversal order in CST [18]. Because sequencing errors can incur *k*-mers matches, the main goal of the binomial test is to distinguish random matches by sequencing errors from true matches for a true strain, which is particularly important for strains of low abundance. Given a node *v*, we examine whether we can reject the null hypothesis that $abund_v$ is generated by sequencing errors.

Specifically, we first round $abund_v$ and $abund_p$ (*p* is the parent node of *v*) to their nearest integers $abund_v'$ and $abund_p'$. Then, given the sequencing error rate *e* (1% by default), we reject the error-caused null hypothesis when the probability of $abund_v$ being generated from sequencing errors is smaller than $\beta$ (0.05 by default). The probability is estimated with

4 $$\begin{aligned} P_{X\tilde{B}(abund_p', 1-e)}(X\le abund_p'-abund_v') \end{aligned}$$

where $B(abund_p, 1-e)$ is the probability mass function of the binomial distribution with $abund_p'$’s trials and the successful rate $1-e$. Failing to reject the null hypothesis indicates that we cannot distinguish low-coverage *k*-mers matches and sequencing noise. Thus, we consider $abund_v$ is just from sequencing errors, and the search will stop for *v*’s descendants. Otherwise, if we succeed in rejecting the null hypothesis, we believe that one or multiple strain clusters in $T_v$ are present in the sequencing data. And the CST search will add *v*’s two child nodes to the end of the BFS queue, preparing to traverse them later. Unlike the traditional binary search tree (BST), the two sibling nodes of the same parent can both reject the error-caused null hypothesis. Therefore, we can traverse all of their descendants, and the search results of CST are probably more than one.

#### Cluster identification

Once we reach a leaf node, we will further examine the *k*-mers matching and abundance estimation statistics using both the leaf node and its ancestor nodes that contain *k*-mers moved from the leaf nodes. If there is only a single leaf node identified, all the nodes along the path from the root to *v* can be used to compute the *k*-mers statistics. However, if there are multiple leaf nodes identified, not all the ancestor nodes should be used. Instead, only the ones that contribute uniquely to the leaf node *v* should be used to compute the final abundance. These nodes can collectively constitute a path $Path_v$, where all of the *k*-mers matches on $Path_v$ only originate from strains in the leaf node *v*. To identify $Path_v$, we first identify the maximum subtree that only contains *v* as the identified node. And $Path_v$ is equivalent to the path between the root of this subtree to *v*. Using the *k*-mers counts of nodes on $Path_v$ all together to estimate the cluster’s abundance will provide higher confidence than using a single leaf node. Take $Path_4$ in Fig. 4 as an example, two leaf nodes 4 and 5 are identified. In this case, $Path_4$ is the root-to-leaf path containing *v* in node 8’s rooted subtree $T_{v_8}$. Subsequently, we can collect all *k*-mers match counts $C_i$ on $Path_v$ to calculate the weighted average fraction of matching *k*-mers $\mathbf {F_v}$ and the weighted average *k*-mers match count $\mathbf {A_v}$:

5 $$\begin{aligned} F_v = \frac{\sum \limits _{i\in Path_v} |C^+_i|}{\sum \limits _{i\in Path_v} |C_i|} \end{aligned}$$

6 $$\begin{aligned} A_{v} = \frac{\sum \limits _{i\in Path_v} |C^+_i|\cdot abund_{i}}{\sum \limits _{i\in Path_v} |C^+_i|} \end{aligned}$$

If $F_v$ is larger than a given cutoff (the default value is 0.4, but users can modify the value to adapt to different conditions), we consider the cluster in *v* is present in the sequencing data. After finishing the CST search, all identified clusters will be output with their estimated abundances (calculated by $A_v$). Besides, when the sequencing data contains multiple strains in different clusters, some FPs will be introduced because of the added *k*-mers during the weak node augmentation. The detailed method to address this problem can be found in Supplementary Section 1.2.

### Strain identification within the clusters

**Fig. 5:**
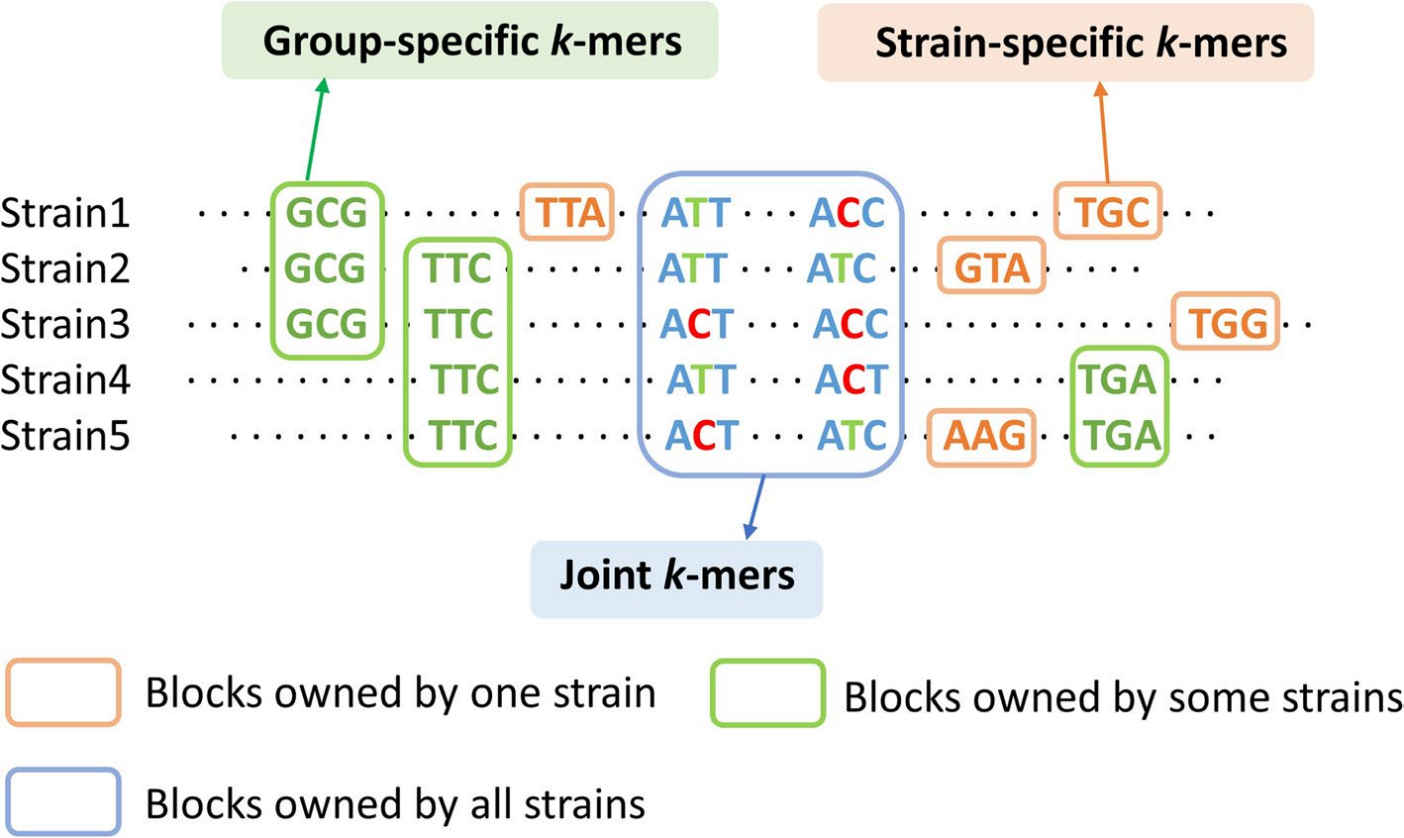
Use strain-specific *k*-mers, group-specific *k*-mers and joint *k*-mers to distinguish five strains in the same cluster. Each strain has a unique *k*-mers combination

The CST is optimized for distinguishing clusters with similarity below a given cutoff. Using CST to distinguish highly similar strains can lead to a large number of weak nodes that cannot be augmented because of the large percentage of shared *k*-mers. Thus, once we pinpoint a cluster, we need a fine-grained method to distinguish highly similar strains. Once we pinpoint a cluster, the number of strains to distinguish is significantly reduced compared to the original problem space. Thus, we can afford to use all *k*-mers with distinguishing power. The first feature used is the unique *k*-mers from strain-specific regions, here we call it the strain-specific *k*-mer. The second feature used is the group-specific *k*-mer, which may come from structural variants (SVs) common to some strains. In a recent study [42], SVs have been used to distinguish different strains. Inspired by that study, we extract group-specific *k*-mers from the SVs shared by some strains. However, relying only on strain-specific and group-specific *k*-mers still suffers from low resolution in some cases. For example, in Fig. 5, both Strain4 and Strain5 have the same group-specific *k*-mers, and when the strain-specific *k*-mers of Strain5 is not present in the sample, we cannot make a fine distinction between the two strains. Therefore, to further improve the resolution, we add joint *k*-mers sets, which contain SNVs and indels from core genomic regions present in all genomes [29, 38, 43]. As shown in Fig. 5, for all joint *k*-mers, although each *k*-mers is not strain-specific, the joint *k*-mers set for each strain is unique. However, the number of joint *k*-mers is often not as many as the first two types of *k*-mers (Supplementary Table S2). They need to be combined together to improve the resolution of identification. By utilizing these three types of *k*-mers, we improve the resolution of identification and reduce the search space at the same time.

To efficiently extract these *k*-mers, we utilize Sibeliaz [44], an efficient tool designed for identifying locally collinear blocks in closely related genomes. Based on the blocks generated by Sibeliaz, we develop a hash-based algorithm to extract these *k*-mers from strain genomes and save them in a matrix for later usage. The algorithm’s main pseudocode is shown in Supplementary Section 1.3. The input to the algorithm is strain genomes within the same cluster and blocks generated by Sibeliaz. By using the efficient hash table, the algorithm can extract target *k*-mers quickly. Finally, all extracted *k*-mers from a cluster are saved in a matrix X of size $M\times N$, where *M* is the number of *k*-mers and *N* is the number of strains in this matrix. Then, X$[i,j]=1$ if strain j has *i*th *k*-mer, otherwise, X$[i,j]=0$. When there are multiple clusters, multiple corresponding matrices are created, respectively.

#### Strain identification using chosen *k*-mers

After extracting the *k*-mers in the previous step, we need to use these features for strain identification. To disentangle complex communities of closely related strains in the same cluster, we apply the iterative matrix multiplication to determine all coexisting strains and predict their relative abundance using elastic net regression.

The main goal of the iterative matrix multiplication is to determine strains in the same cluster by using three types of *k*-mers (Fig. 5) in the *k*-mers matrix. To achieve this goal, we compare the *k*-mers in the sample to those in the *k*-mers matrix X using an iterative strategy similar to that of QuantTB [37]. The method is described as follows. Given the cluster selected by the tree search and *k*-mers from its *k*-mers matrix X, we will apply Jellyfish [41] to count all these selected *k*-mers in the sequencing data. Denote the occurrences for all selected *k*-mers from the Jellyfish as a vector *y*: $y= (y_{1}, y_{2}, y_{3}, ..., y_{M})^{\textrm{T}}$, where $y_{i} \ge 0$ and represents the occurrences of the *i*th *k*-mers in the matrix. However, the overlapping *k*-mers from other identified clusters could lead to false *k*-mers matches or wrong abundance estimation. To remove the influence of other clusters, if one *k*-mers is found in other clusters detected by the tree search, its occurrence will be replaced with 0. For the $M\times N$ *k*-mers matrix X, its *j*th column X[ : , *j*] is defined as:

7 $$\begin{aligned} X[:,j] = (X[1,j], X[2,j], X[3,j], ..., X[M,j])^{\textrm{T}}, \nonumber \\ j = 1, ..., N \end{aligned}$$

Based on X and *y*, we use the iterative matrix multiplication, which can detect all possible strains in a sample accurately and quickly. Given X and *y*, the function will calculate a score $f_j=$ X$[:,j] \cdot y$ for each strain. Note that we regard values beyond the 5th and 95th percentile to be outliers, and we will set the value of all outliers as 0. The function will rank all strains according to their scores. After ranking, the function will output the top 1 strain in the ranked list and then update *y* by replacing the occurrences of all *k*-mers in identified strain with 0. This process is repeated. It continues to calculate the score and identify the most likely strain in each iteration until the occurrences of *k*-mers with nonzero value is below the given threshold, whose default value is $31*40=1240$ *k*-mers. All the experiments in this work are conducted using the default cutoffs.

Knowing the possible strains in the sample, we use the elastic net regression model to predict sequencing depths and relative abundances of identified strains. We choose the elastic net model instead of the Lasso model because the Lasso model tends to underestimate the number of strains, leading to a decrease in recall. After iterative matrix multiplication, we obtain the filtered *k*-mers matrix X$'=M \times N'$, where $N'$ is the number of identified strains. Sequencing depths, which are the regression coefficients $\beta '$, are predicted by minimizing the elastic net penalized residual sum of squares:

8 $$\begin{aligned} \beta ' = \underset{\beta \in {X'}^{N'}|\beta _k>=0}{\textrm{argmin}} \left\| y-X'\beta \right\| _{2}^{2}+\lambda (\alpha \left| \beta \right| _1+\frac{(1-\alpha )}{2}\left| \beta \right| ^2 ) \end{aligned}$$

$\alpha$ and $\lambda$ are two important parameters that will affect the model performance and therefore need to be tuned. We have designed a function to tune the $\alpha$ and $\lambda$ based on cross-validation to obtain the model with the lowest predictive error. Given this best model, we calculate the strain relative abundance $a=(a_1, a_2, a_3, ..., a_{N'})$ by normalizing the regression coefficients $\beta '$ of the model. However, if multiple clusters are detected by the tree search, the relative abundance of one strain *i* will be recalculated according to the abundance of clusters. So, the final relative abundance (RA) of each strain *i* is calculated as:

9 $$\begin{aligned} RA_i = \frac{a_i*C_i}{\sum _{j=1}^na_j*C_j} \end{aligned}$$

where *C* is the abundance of the cluster predicted by the tree search, and *n* is the total number of all identified strains.

**Table 1:**
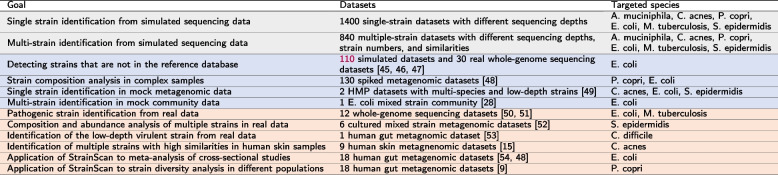
The overview of all experiments. Gray block: simulated data; Blue block: mock or spiked datasets; Orange block: real sequencing datasets. The strain composition of all datasets is provided by the simulation process or by the original publications [9, 15, 28, 45–54]

### Prediction accuracy evaluation

In order to test the performance of each method, we calculated the recall, precision, and F1 score for every test category. True positive (TP) refers to the number of correctly identified strains. False negative (FN) refers to the number of strains present in the sample but missed by a tool. False positive (FP) is the number of misidentified strains.

$$\begin{aligned} Precision = \frac{TP}{TP+FP},\ Recall = \frac{TP}{TP+FN} \end{aligned}$$

$$\begin{aligned} \ F1 = \frac{2*Precision*Recall}{Precision+Recall} \end{aligned}$$

In all experiments, we used the Jensen-Shannon divergence (JSD) [55] to measure the distance between the true and predicted relative abundance. If the predicted and true abundance have different dimensions, we will calculate JSD by adding zeros to the one with the lower dimension. Suppose there are two probability distributions T and P, their Jensen-Shannon divergence is a value between [0, 1] and is defined as:

$$\begin{aligned} JSD(T\ ||\ P) = \frac{1}{2}D(T\ ||\ K) + \frac{1}{2}D(P\ ||\ K) \end{aligned}$$

where

$$\begin{aligned} K = \frac{1}{2}(T\ + \ P) \end{aligned}$$

and $D(T\ ||\ K)$ is called the Kullback-Leibler divergence from T to K and it is defined as:

$$\begin{aligned} D(T\ ||\ K) = \sum \limits _{i}T(i) \log {\frac{T(i)}{K(i)}} \end{aligned}$$

## Results

Because StrainScan focuses on identifying known strains, we test the performance of StrainScan on six bacteria that can pose computational challenges for strain-level analysis. All chosen bacteria have at least 100 sequenced strains. Some of them have a large number of known strains such as *E. coli* and *S. epidermidis*. Some have strains with extremely high sequence similarity, such as *M. tuberculosis*. In addition, we choose bacteria that usually inhabit different ecosystems such as the human gut and human skin, including *A. muciniphila*, *P. copri*, and *C. acnes*. We carried out multiple experiments to evaluate StrainScan. The overview of all experiments is summarized in Table 1. First, we tested the ability of StrainScan in identifying one strain and multiple co-existing strains in simulated data and spiked metagenomic data. We generated different datasets by configuring the parameters such as strain similarity and strain sequencing depth, which help us compare the performance of different tools in difficult scenarios. Second, we tested StrainScan in three mock community datasets, which allow us to evaluate different tools in real sequencing data with known strain composition. Third, we tested StrainScan in 94 real sequencing datasets with various depths (Supplementary Table S3) [21, 29, 37, 39, 52]. Because there is usually no ground truth for the strain composition in the real sequencing data, we choose the datasets that had been analyzed by the authors of the data. By comparing the analysis results, we are able to draw some conclusions about different tools’ performance. In these experiments, we used the F1 score, precision, recall, and Jensen-Shannon divergence as the evaluation metrics, which are defined in the “Methods” section. We benchmarked StrainScan against popular reference-based strain-level analysis tools including Krakenuniq (V0.5.8) [30], StrainSeeker (V1.5) [31], Pathoscope2 (V2.0.6) [39], Sigma (V1.0.1) [38], StrainGE (V1.1.5) [28], and StrainEst (V1.2.4) [29].

Evaluation of StrainGE and StrainEst at two resolution levels Among these tested tools, StrainGE and StrainEst group strains into clusters and only keep a representative strain for each cluster [28, 29]. Thus, we evaluated their performance at two resolution levels: strain-level and cluster-level. Strain-level evaluation only counts the output as true positive (TP) if the identified representative strain is identical to the present strain. Cluster-level evaluation counts the output as TP if the returned representative strain is in the same cluster as the target strain. Correspondingly, the definition of FP is also more lenient at the cluster level. For all other tools, we used the strain-level resolution to calculate the related statistics. Below we present the experimental results.

### Reference database construction

For all the species tested in this work, we created the reference strain genome database as comprehensively as possible. Thus, we downloaded all complete and draft genomes from the NCBI RefSeq database for the tested bacteria. But there are 25,349 *E. coli* genomes, requiring >1TB memory. Due to the constraints of our hardware resources, we only used the complete *E. coli* genomes from RefSeq. Similar to *E. coli*, our hardware resources prevent us from using all draft and complete genomes for *M. tuberculosis*. In addition, some available genomes for *M. tuberculosis* only differ by fewer than 10 positions [37]. These near-identical strains will be clustered in our pre-processing step. Thus we computed pairwise Jaccard similarities of all *M. tuberculosis* strains using Dashing [40] and performed complete-linkage clustering using a *k*-mers Jaccard similarity threshold of 99%. Then, we only kept the strain with the highest average similarity to all other genomes in that cluster. As a result, 792 out of 6,752 genomes are kept for *M. tuberculosis*.

**Table 2:** The summary statistics of the reference genomes for 6 tested bacteria. “Average Jaccard similarity” is obtained by calculating the average of *k*-mers Jaccard similarity of all strains using Dashing [40]

Species	Average genome size	Average Jaccard similarity	# of input genomes	# of representative strains (StrainEst)	# of representative strains (StrainGE)
*A. muciniphila*	2.7 Mb	41.61%	157	42	48
*C. acnes*	2.5 Mb	63.69%	275	25	18
*P. copri*	3.5 Mb	30.94%	112	42	43
*E. coli*	4.9 Mb	40.62%	1433	333	662
*M. tuberculosis*	4.3 Mb	94.14%	792	63	10
*S. epidermidis*	2.5 Mb	58.20%	995	52	221

The final numbers of the strains and their other properties were recorded in Table 2. The numbers of genomes that are used as input to all tested tools are shown in the column “# of input genomes.” As mentioned before, StrainEst and StrainGE will cluster the input strain genomes and only keep one representative strain selected from each cluster in their final databases. As a result, there are significantly fewer strains left (Table 2). When we take a closer look at the clusters of StrainEst and StrainGE, we can observe significant differences in the gene contents and SNVs between the representative strain and other strains in the same cluster (Supplementary Fig. S3, S4, S5). For some species, there are over 5000 SNVs between the actual strain and the representative strain. Within the same cluster, the longest strain can have more than 1000 genes than the shortest strain. As one recent study [56] shows, “singletons” (the unique genes) found in specific strains are very important to the understanding of strain properties. Thus, these large gene content variations between strains in the same cluster may lead to different properties and functions. A notable example is that two highly similar strains, *E. coli* CFT073 and *E. coli* Nissle 1917, which are pathogenic and probiotic strains respectively, are grouped in the same cluster by StrainEst and StrainGE.

StrainScan also groups strains into clusters before conducting intra-cluster strain identification. Our experimental results show that the cluster search using CST can achieve 100% accuracy for all tested bacteria. For most bacteria, StrainScan has more fine-grained clusters than StrainEst and StrainGE (Supplementary Fig. S6), indicating a higher resolution at the cluster level.

**Fig. 6:**
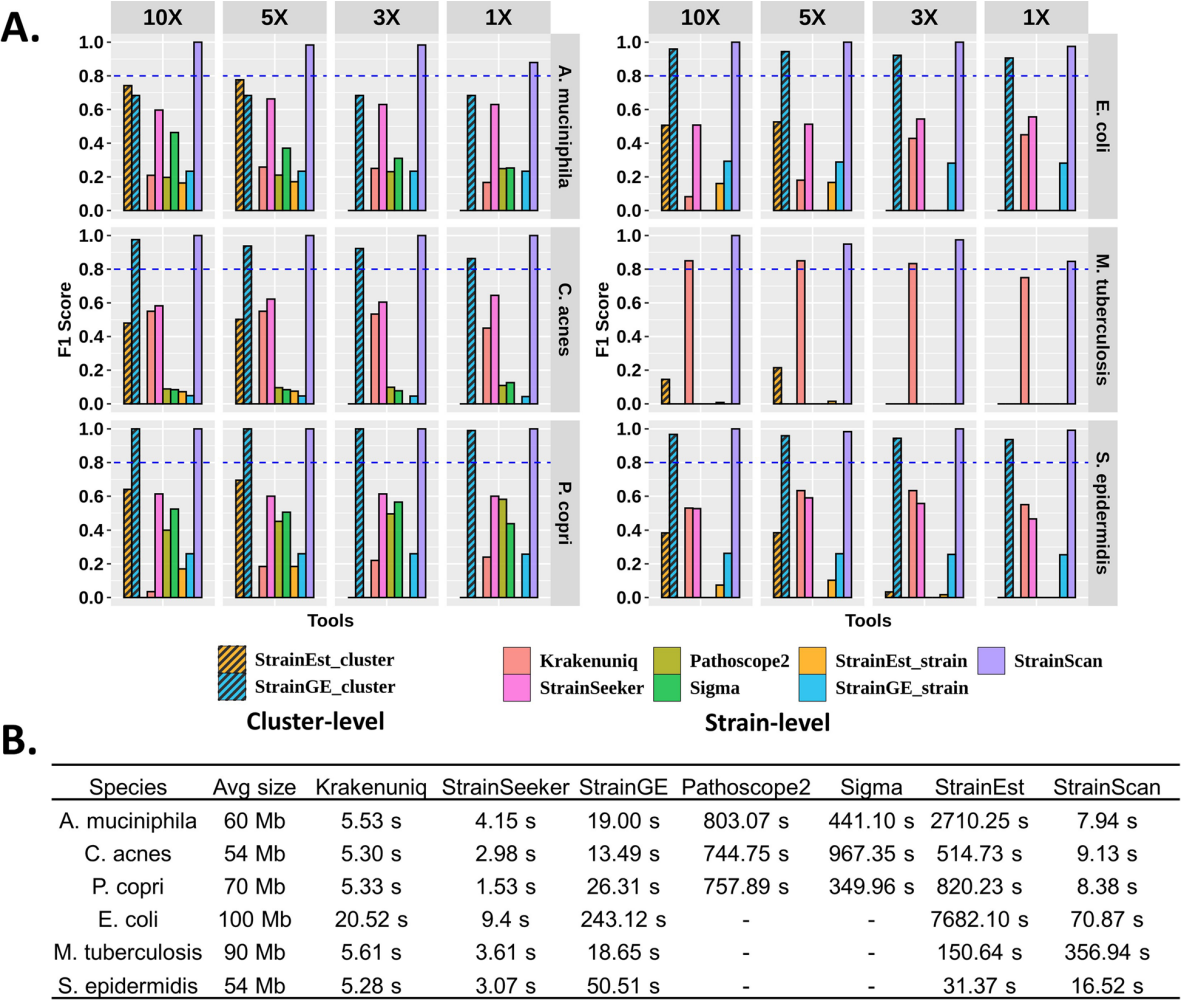
**A** The F1 score of 7 tools on “single-strain” simulated datasets under different sequencing depths. **B** Running time comparison of 7 tested tools. Sigma and Pathoscope2 have no values on some datasets because they are too computationally expensive to construct databases for the corresponding bacteria or to identify strains from simulated reads

### Detecting a reference strain from simulated reads

The purpose of this experiment is to test StrainScan and other tools for identifying the present strain in a sample. There are two challenges. The first challenge is to distinguish the true strain from other highly similar peers. The second challenge is to identify a strain with low depth. Thus we generated multiple datasets with different sequencing depths.

For each bacterium, we randomly picked a reference strain and used its simulated short reads as input to all tools. In order to avoid any data-related bias, we repeated the experiment 60 times, with a strain randomly picked each time. For *P. copri*, we only repeated the experiment 50 times because it has a small number of genomes. For each selected strain, we simulated reads with different sequencing depths (10X, 5X, 3X, 1X). Thus, there were 1400 datasets in total. For each dataset, we simulated Illumina reads using ART [57] with the following parameters: -p -l 250 -f *depth* -m 600 -s 150, where *depth* is the specified sequencing depth. StrainScan and the other six programs were used to identify strains from these simulated reads. As Sigma and Pathoscope2 are computationally expensive, we were not able to construct their databases for *E. coli*, *S. epidermidis*, and *M. tuberculosis*.

The F1 score of each program is shown in Fig. 6A. The TP, FN, FP, recall, and precision are recorded in Supplementary Table S4. Of these selected strains, some of them have more than 99.5% *k*-mer-based Jaccard similarity with at least one other reference strain genome. As a result, several tools have low F1 scores. StrainScan achieves near-perfect F1 scores on all datasets when the depth is higher than 1X. However, for the species containing highly similar strains (*A. muciniphila* and *M. tuberculosis*), the F1 score of StrainScan drops a little when the depth is 1X. When the depth is low and the strain similarity is very high, the CST algorithm fails to identify some strains due to low unique coverage, and thus the recall drops. Nevertheless, StrainScan still has the best F1 score for these species. Currently, the minimum depth accepted by StrainScan is 1X and the performance will drop rapidly if the depth is lower than 1X.

While Pathoscope2 and Sigma achieve relatively high recall, there are a lot of FPs in their output, which makes their precision much lower than other tools. Krakenuniq achieves a higher F1 score for the datasets where many strains have unique *k*-mers. However, highly similar strains of some bacteria lead to low recall and precision for Krakenuniq. StrainSeeker and StrainEst also have a large number of FPs, which leads to low precision. In addition, StrainEst is not able to identify strains with depths lower than 5X. StrainGE performs on par with StrainScan at the cluster-level resolution in many datasets. However, it returns more FPs when the sequencing depth decreases. For example, the cluster-level precision of StrainGE on *C. acnes* drops from 0.95 to 0.76 when the strain depth decreases from 10X to 1X. In contrast, StrainScan does not generate any FPs as the depth decreases (Supplementary Table S4). Even at the cluster level, the performance of StrainGE is not ideal for *M. tuberculosis* because those strains have high *k*-mers Jaccard similarities. Out of the tested tools, StrainSeeker tends to return multiple strains of the same score. This is similar to returning a representative strain by StrainEst and StrainGE, where a finer distinction between a group of strains is not provided. As a result, these tools suffer from low resolution. For example, StrainGE returns a representative strain for a cluster of size around 200 for *E. coli* (Supplementary Fig. S7). Based on our previous analysis of the genetic differences of the strains in those clusters (Supplementary Fig. S3), the resolution is not ideal.

Figure 6B shows the running time of different tools. StrainScan is efficient in all tested bacteria except *M. tuberculosis*. Due to high *k*-mer-based Jaccard similarities across strains of *M. tuberculosis*, StrainScan assigned most of the strains to one big cluster with a significant number of *k*-mers (Supplementary Fig. S6), and thus StrainScan took more time to distinguish them. Nevertheless, StrainScan still has the best recall and precision in terms of the identification of *M. tuberculosis* strains. All the strain identification experiments were tested on an HPCC CentOS 6.8 node with 2.4Ghz 14-core Intel Xeon E5-2680v4 CPUs and 128 GB memory. We used 8 threads for all tools. In summary, StrainScan is able to achieve higher precision without sacrificing resolution, even when the true strain has peers of high sequence similarity.

### Detecting co-existing strains from simulated data

It has been shown that human-associated microbiota is often a complex mixture of closely related strains of the same species [15]. To quantitatively compare the performance of Krakenuniq, StrainSeeker, StrainGE, StrainEst, and StrainScan on identifying multiple strains of the same species, we generated simulated datasets containing 2, 3, and 5 randomly selected strains from six bacteria. Because Sigma and Pathoscope2 took too long to process these datasets, they were not included in this experiment.

To investigate how the similarities between the strains affect the tool’s performance, we used two strategies in the selection of multiple strains. During the clustering step of StrainScan, strains with *k*-mer-based Jaccard similarity greater than or equal to 95% (corresponding to an approximate ANI of 99.89%) are grouped into the same cluster. Therefore, it is more difficult to identify and distinguish the co-existing strains that are in the same cluster than those from different clusters. To consider different levels of difficulty, our first strategy randomly picked strains from different clusters while the second strategy selected different strains from the same cluster. For each strategy, we randomly selected 2, 3, and 5 strains (3 groups) and simulated the short reads using different coverage profiles: 100X and 10X for 2 strains, 100X, 50X, and 10X for 3 strains, and 100X, 70X, 50X, 20X, and 10X for 5 strains. Other read simulation parameters are the same as the “single-strain” experiment. Then we repeated the experiment 10 times by choosing another group of strains. Ultimately, for each bacterial species, we generated 30 sets of data containing different numbers of strains using the first and the second strategies, for a total of 60 sets of data. So there were a total of 360 (60 × 6) simulated datasets for the six bacterial species.

Cluster-level performance evaluation for multi-strain cases If a sample contains *n* strains from the same cluster defined by StrainGE or StrainEst, only one representative strain will be returned based on these tools’ design. Using this one representative strain will lead to a very small recall for multi-strain experiments. To avoid that, the returned representative strain will be counted *n* times, which usually makes the recall 1.0 in these tools’ favor. Because our clusters have greater granularity than the ones defined by StrainGE and StrainEst, the samples that are simulated from strains of the same cluster all belong to this case.

**Fig. 7:**
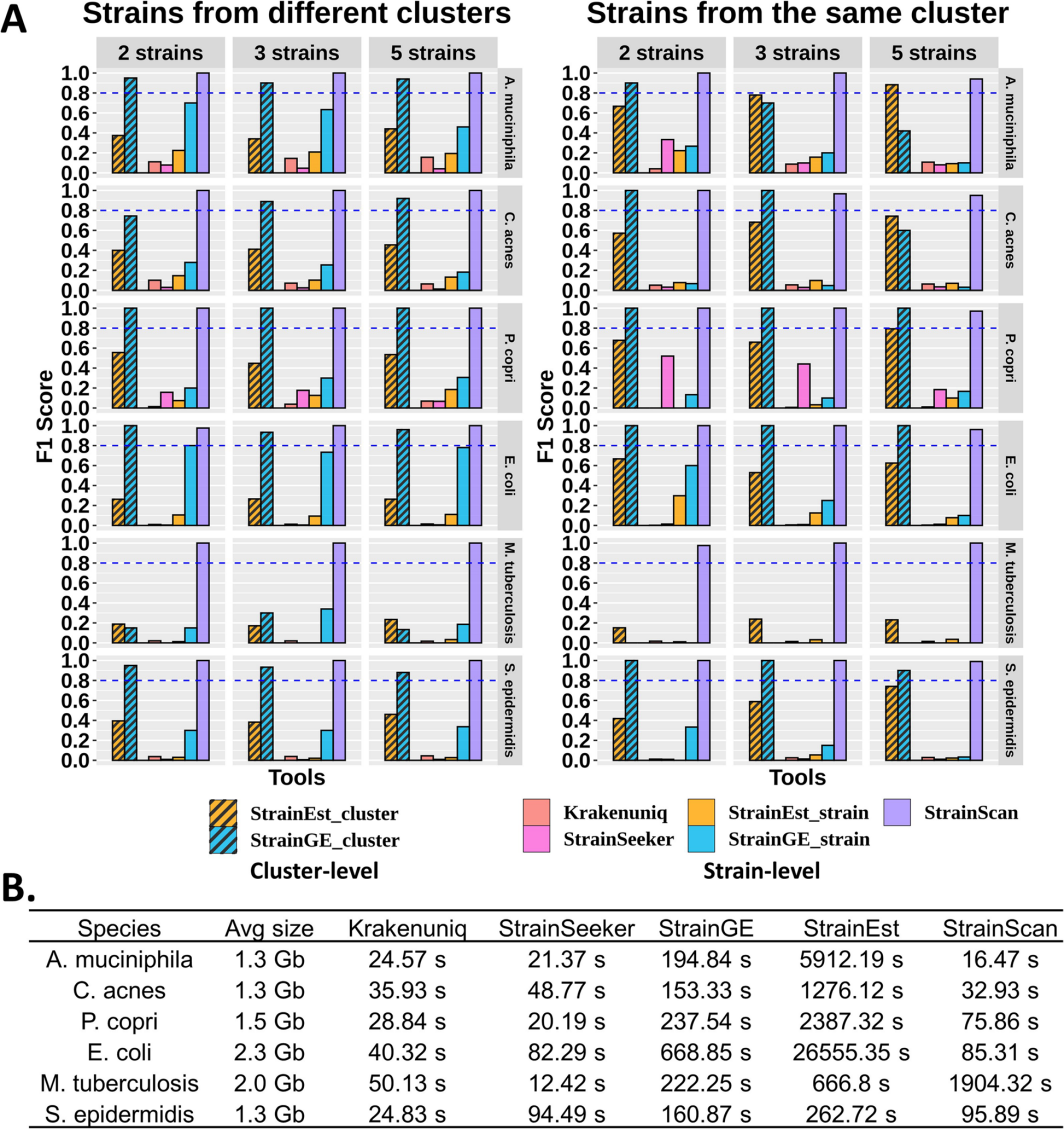
**A** The F1 score of 5 tools on “multiple-strain” simulated datasets. The “cluster” in the title refers to the clusters generated by the CST algorithm. There are 60 sets of simulated reads containing 2, 3, and 5 strains with different similarities for each bacterial species. Note that StrainSeeker is not able to identify strains of *M. tuberculosis* and therefore, the related scores are 0. **B** Running time comparison of 5 tested tools

Benchmark results The F1 score comparison of different tools is shown in Fig. 7A. The TP, FN, FP, recall, and precision are recorded in Supplementary Table S5 and S6. As shown in Fig. 7A, StrainScan achieves a near-perfect F1 score on all tested datasets. StrainGE has the same cluster-level F1 scores as StrainScan for experiments on *P. copri* and *E. coli*. Its cluster-level F1 score generally decreases with the increase of the similarity of the input strains, which can be observed by comparing the left and right panels of Fig. 7A. For example, its cluster-level recall on 5-strain datasets of *A. muciniphila* dropped from 0.94 to 0.42 (Supplementary Table S6). Similar to single-strain experiments, StrainGE’s cluster-level performance on *M. tuberculosis* is still poor. StrainEst has many FPs in its output, which leads to low precision and F1 score. However, because of the way we evaluate the cluster-level performance, StrainEst’s cluster-level recall for strains of the same cluster appears higher than strains of different clusters, leading to better F1 scores (the right panel of Fig. 7A). The remaining tools have lower F1 scores in general. Among them, Krakenuniq performed better in identifying strains from different clusters than in identifying strains from the same cluster, which was in line with its method. StrainSeeker has a lot of FPs in all tested datasets and its recall is also low for most tested bacteria, indicating that it was unsuitable for identifying multiple strains. Besides, by analyzing the number of returned strains of the same score (StrainSeeker) and the number of strains in a returned cluster (StrainEst and StrainGE), we also found that high similarities between strains further reduce the resolution of StrainSeeker, StrainGE, and StrainEst (Supplementary Fig. S8). Overall, compared to other tested tools, StrainScan achieves more than $20\%$ improvement in F1 score at the strain level for all datasets while keeping the high resolution.

StrainScan is also faster than StrainGE and StrainEst except *M. tuberculosis* (Fig. 7B). As mentioned in the previous section, due to the high similarity between strains of *M. tuberculosis*, StrainScan sacrifices the computational efficiency to distinguish the strains in the same big cluster and thus took longer to process *M. tuberculosis*.

**Fig. 8:**
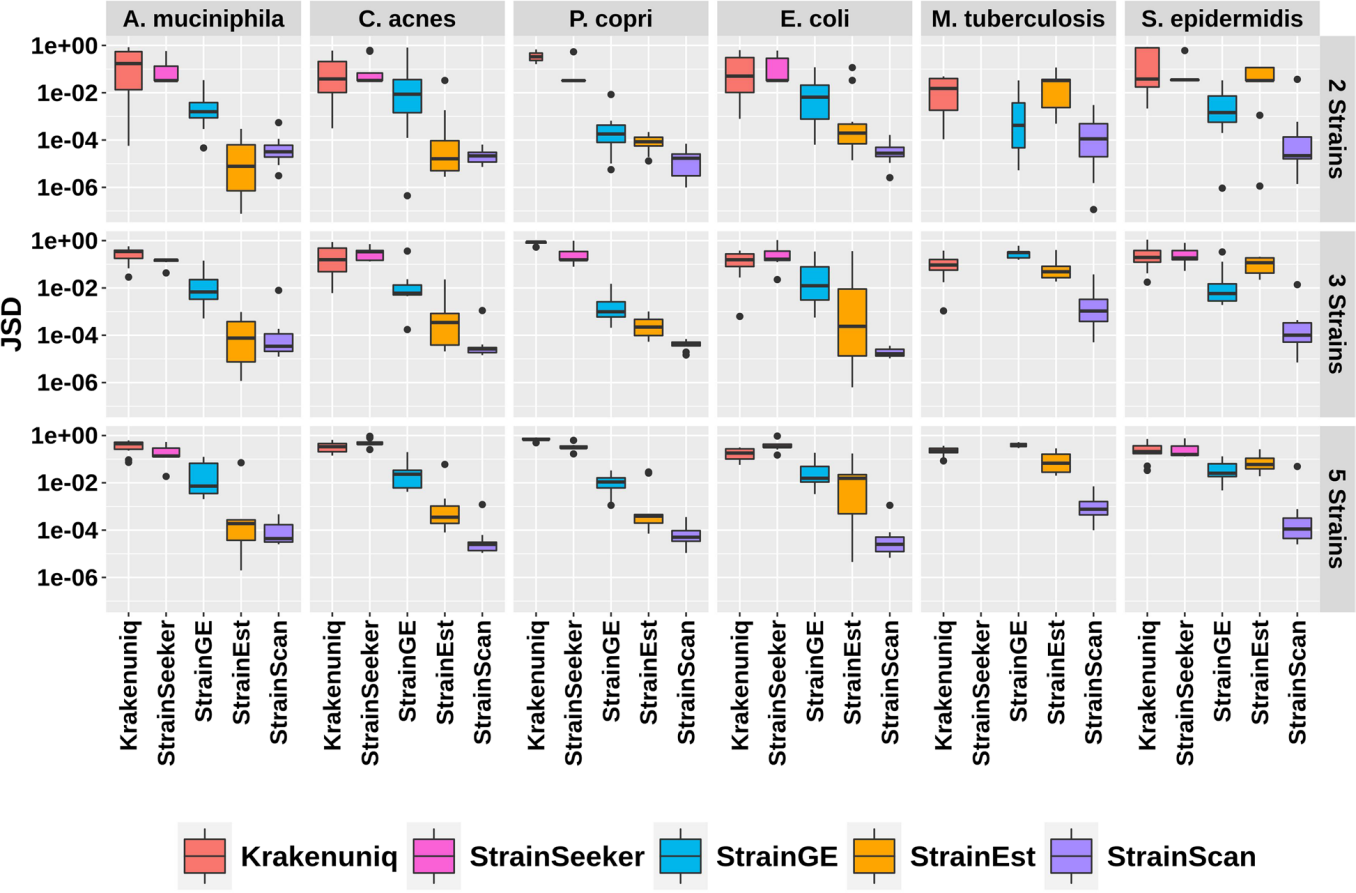
The Jensen-Shannon Divergence (JSD) of 5 tools between the ground truth and predicted relative abundance. Because StrainGE and StrainEst always return one representative strain for strains from the same cluster, the result shown here only includes strains from different clusters. The complete results containing strains from the same cluster and different clusters can be found in Supplementary Fig. S9

Relative abundance computation In order to measure the accuracy of the predicted strain profiles in synthetic data sets, we computed the Jensen-Shannon divergence (JSD) between the actual and the inferred frequencies. In case the dimension of predicted and true relative abundance may be different, we add zeros to the one with a lower dimension to calculate JSD. In the case of StrainGE and StrainEst, the abundance is calculated at the cluster level. For example, if there are two strains in a sample and these two strains have the same representative strain, then we use the representative strain and its abundance twice for calculating JSD. This leads to a smaller JSD than setting one of the strain’s abundance as zero. The result is shown in Fig. 8. In all cases the strain distribution reconstructed by StrainScan had high precision, with a median of Jensen-Shannon divergence (JSD) $<0.01$. StrainGE and StrainEst had worse performance in quantifying the composition with the increased number of strains. For species containing highly similar or a large number of strains like *E. coli*, *M. tuberculosis*, and *S. epidermidis*, StrainScan shows a clear advance over StrainGE and StrainEst. For StrainSeeker and Krakenuniq, the JSD was bigger than 0.1 in most cases, indicating that these tools cannot accurately quantify the composition of strains. With increasing strain numbers and similarity, the median of JSD values of most tested tools increases while StrainScan’s JSD doesn’t fluctuate much in all cases. These results show that StrainScan can better quantify the composition of complex samples than other tools, even for samples containing highly similar strains.

Low-depth experiments for multiple strains To evaluate the ability of different tools in identifying multiple strains at a lower depth, we simulated additional short reads from previously selected 2 strains using different coverage profiles. Then, we benchmarked all tools using these datasets. The results revealed that StrainScan demonstrated a clear advantage in identifying low-depth strains from many highly similar strains. For example, when identifying *C. acnes* strains with 10X and 1X coverage, StrainScan achieved an F1 score of 0.98, while Krakenuniq and StrainGE, in second and third place respectively, achieved F1 scores of only 0.88 and 0.81 (see Supplementary Table S7). Overall, StrainScan shows competitive performance in identifying low-depth multiple strains (Supplementary Table S7 and S8). Additional details regarding this experiment can be found in Supplementary Section 2.1.

**Fig. 9:**
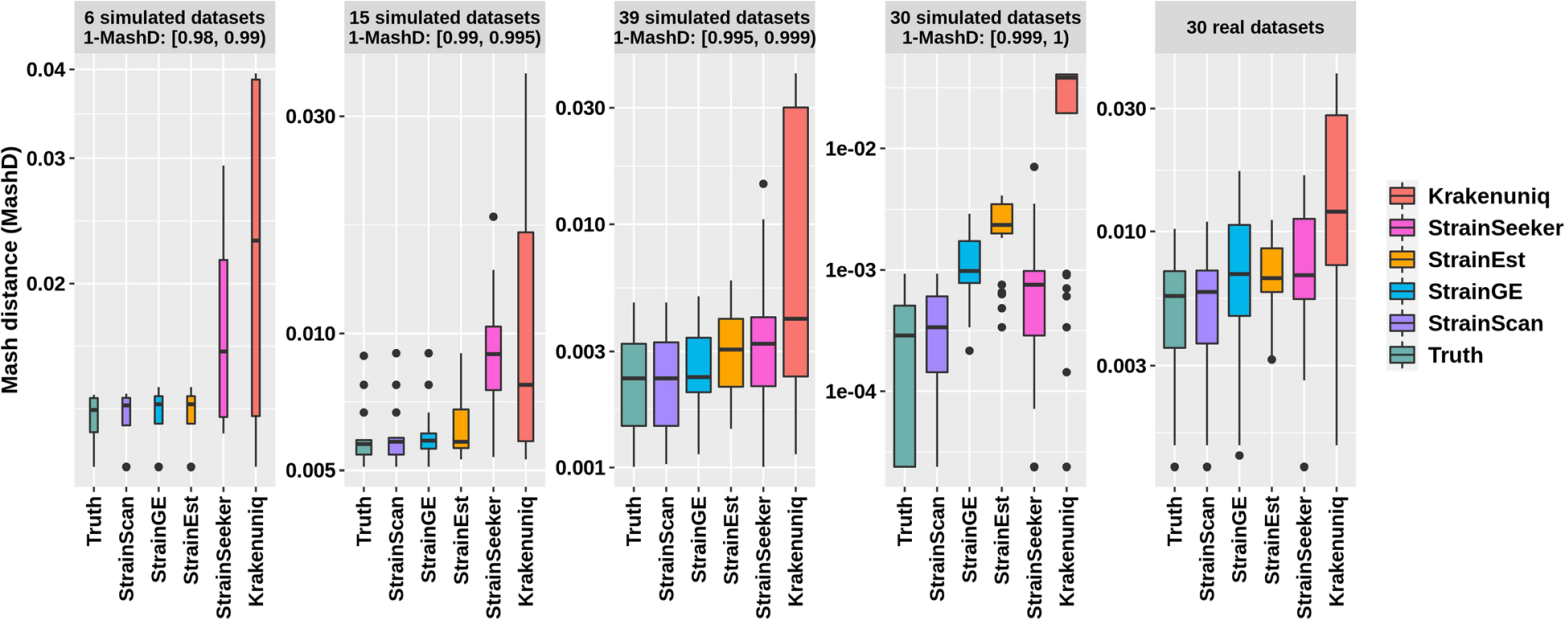
The Mash distance between the identified strains and the ground truth in 90 simulated and 30 real *E. coli* datasets. The simulated datasets are divided into four groups according to the “1-Mash distance” between the actual strain and its best match among all strains in the database of StrainScan

### What if the actual strain is not in the reference database?

When a target strain is not included in the database, we expect that the strain identification tools can return its best match in the database. To evaluate the performance of different tools in identifying the best match, we downloaded 90 complete *E. coli* genomes that were released in 2022 from NCBI (Supplementary Table S9). Because the *E. coli* reference databases of all tools were constructed using complete genomes available up to 2021, all these genomes are not in our constructed databases. Then, we simulated reads of 10X coverage from the 90 *E. coli* genomes and used them as input to all tools. For each dataset, the “truth” in Fig. 9 is the Mash distance between the actual strain and its best match in all 1433 *E. coli* strains (Table 2). We then recorded the Mash distance between the returned strain and the actual one for each dataset in the first four panels of Fig. 9. It should be noted that this is a single-strain experiment, and thus the Mash distance is computed using the returned strain (including the representative strain) and the actual strain. Out of these 90 strains, 54 strains’ clusters are missing from the reference database of StrainScan. Nevertheless, StrainScan can correctly identify the closest matched cluster in the database for all these 54 strains (Supplementary Table S9). Furthermore, strains identified by StrainScan have a smaller Mash distance to the actual strain than other tools. Although the closest matches identified by StrainGE and StrainEst also have similar Mash distance to “truth” when “1-MashD” ranges from 0.98 to 0.99, their performance drops when “1-MashD” increases. For example, when “1-MashD” between the actual strain and the best match is within (0.999, 1), StrainGE and StrainEst tend to return a representative strain from a relatively large cluster (Supplementary Table S9). As a result, these tools returned representative strains with a larger Mash distance than the actual strain. In contrast, StrainScan achieves intra-cluster strain identification and returns more accurate best matches.

For a more robust test, we downloaded 30 real *E. coli* whole-genome sequencing data with draft genomes in NCBI. To avoid data-related bias, the 30 real sequencing datasets are randomly selected from 3 different projects (PRJNA509690, PRJNA479542, PRJEB21464), and their coverage ranges from 20X to 94X [46, 47]. According to NCBI, the assembly levels of these draft genomes are all “Scaffold” rather than “Complete”. Thus, all these genomes are also not in the *E. coli* reference databases that were constructed using complete genomes. We further compared the RefSeq accession of these 30 draft genomes with the genomes in our constructed databases and did not find any matches. Then, we applied all tools to these real datasets and compared the identified strains with the draft genomes (the last panel of Fig. 9). As a result, StrainScan still returns more accurate best matches than other tools, demonstrating its utility in real applications.

Given that multiple strains lacking reference sequences in the database may coexist within a sample, we conducted an additional 2-strain experiment using 90 *E. coli* genomes from the single-strain experiment. The results show that StrainScan achieves a 95% F1 score in identifying multiple strains without reference genomes in the database, while the second-best tool, StrainGE (cluster-level), only achieves a 78% F1 score (see Supplementary Fig. S10). Furthermore, StrainScan did not produce any false positive identifications in all of the tested datasets, and the strains identified by StrainScan had a smaller Mash distance to the ground truth than other tools (see Supplementary Table S10). Additional details regarding this experiment can be found in Supplementary Section 2.2.

### Assessment of StrainScan on spiked metagenomic sequences

While previous experiments mainly used simulated or real whole genome sequencing data, we now evaluate whether Strainscan keeps the same performance on metagenomic data, which contains reads from different species. To this end, we conducted an experiment using spiked metagenomic data. Specifically, by mixing simulated datasets of *P. copri* and *E. coli* with real data, we generated 130 spiked metagenomic data, which we subsequently analyzed using StrainScan. The results show that StrainScan has the same results on these spiked metagenomic datasets as on the simulated whole genome sequencing data, demonstrating the robustness of StrainScan on complex samples (Supplementary Table S11). Further details regarding this experiment can be found in Supplementary Section 2.3.

### Evaluation of StrainScan on mock data

The HMP mock data In this experiment, we tested StrainScan on two samples from the Human Microbiome Project [49]. They contain 21 known organisms with even (SRR172902) or staggered composition (SRR172903). Out of the 21 organisms, 3 bacteria (*E. coli*, *C. acnes*, and *S. epidermidis*) represent hard cases for strain-level analysis, and we have established reference indexing structures for them. Thus, we conducted a strain-level analysis using StrainScan for the three bacteria. According to the given data description, each bacterium has only 1 strain in these two datasets. Although this is a single-strain detection, this test is challenging because some targets have low abundance in the samples. For comparison, we also used Krakenuniq, StrainSeeker, StrainGE, and StrainEst to identify the strains of these bacteria in these two datasets. The results of these 5 tools are shown in Table 3.

**Table 3:** Analysis of two mock communities from the HMP project. “#”: there are multiple hits with identical scores in the output. “NA”: missing values. Bold font: Mash distance of predicted dominant strain to the truth is $< 0.05\%$. For StrainSeeker, we took the strain with the smallest Mash distance to the truth as the predicted dominant strain. The two numbers in parentheses represent the number of multiple hits and the average Mash distance between all hits and the ground truth, respectively

Samples	Tools	Species	Predicted strains	Mash distance to the truth	Running time (s)
SRR172902 (Even)	Krakenuniq	*C. acnes*	GCF_003384685	0.0165552	28.89
		*S. epidermidis*	GCF_001068615	0.0165552	34.17
		*E. coli*	GCF_006364695	0.0165552	46.09
	StrainSeeker	*C. acnes*	GCF_000008345	**0**	11.42
		*S. epidermidis*	GCF_900458515 (#)	**0.0002398 (3, 0.00027)**	14.21
		*E. coli*	GCF_000005845 (#)	**0.0000238 (119, 0.00045)**	13.71
	StrainGE	*C. acnes*	GCF_000008345	**0**	146.33
		*S. epidermidis*	GCF_900458515	**0.0002398**	192.81
		*E. coli*	GCF_001308125	**0.0000476**	429.02
	StrainEst	*C. acnes*	GCF_005937545	**0.0002883**	743.95
		*S. epidermidis*	GCF_000751035	0.0068392	57.75
		*E. coli*	NA	NA	5307.61
	StrainScan	*C. acnes*	GCF_000008345	**0**	22.61
		*S. epidermidis*	GCF_900458515	**0.0002398**	36.94
		*E. coli*	GCF_002953895	**0.0000476**	114.61
SRR172903 (Staggered)	Krakenuniq	*C. acnes*	GCF_003384685	0.0165552	22.79
		*S. epidermidis*	GCF_001068615	0.0165552	17.64
		*E. coli*	GCF_006364695	0.0165552	38.76
	StrainSeeker	*C. acnes*	GCF_000008345	**0**	12.45
		*S. epidermidis*	GCF_900458515 (#)	**0.0002398 (3, 0.00027)**	18.31
		*E. coli*	GCF_000005845 (#)	**0.0000238 (119, 0.00045)**	12.43
	StrainGE	*C. acnes*	GCF_000008345	**0**	175.23
		*S. epidermidis*	GCF_900458515	**0.0002398**	232.56
		*E. coli*	GCF_001308125	**0.0000476**	459.44
	StrainEst	*C. acnes*	NA	NA	239.08
		*S. epidermidis*	GCF_000751035	0.0068392	152.02
		*E. coli*	GCF_000750555	**0.0000238**	9414.24
	StrainScan	*C. acnes*	GCF_000008345	**0**	20.49
		*S. epidermidis*	GCF_900458515	**0.0002398**	59.26
		*E. coli*	GCF_002953895	**0.0000476**	117.49

For all tested species, StrainScan correctly identified the presence of one dominant strain that is highly similar (Mash distance to the truth $<0.05\%$) to the bona fide strain. Besides StrainScan, StrainSeeker and StrainGE also returned strains that are highly similar to the ground truth. However, the output of StrainSeeker often contains multiple hits with identical scores, making accurate evaluations difficult. For example, it returns 119 *E. coli* strains in two tested datasets, which makes it hard for users to know the actual strain present in these samples. In Table 3, we take the strain with the smallest Mash distance to the truth as the predicted dominant strain by StrainSeeker. StrainGE also returns strains with a small Mash distance to the ground truth. Of the two remaining tools, StrainEst was unable to identify low-abundance strains and it took a long time to run, while Krakenuniq returned results that differed significantly from the ground truth.

The *E. coli* mock community with multiple strains To evaluate each tool’s performance on a real-world sample with known multi-strain composition, we downloaded a mock community sequencing dataset (SRR13355226), which contains a large number of reads from the host (i.e., human) as well as four different *E. coli* strains. All the reads are used as input to all tools. StrainScan was the only tool that identified four strains at the strain level correctly, with no false positive identifications (Supplementary Fig. S11). StrainGE correctly identified four representative strains and had no false positive identifications. However, there are 136 different genes between the representative strain identified by StrainGE and the true strain. The genes are predicted using Prokka [58] and the comparison analysis is finished by Roary [59]. Though StrainEst also correctly identified four representative strains, it returned many FPs. The remaining two tools did not correctly identify all four strains, among which StrainSeeker’s output contained multiple hits with identical scores, while Krakenuniq reported many false positive strains.

### StrainScan detects the pathogenic strain from real sequencing data

To illustrate the potential application of StrainScan in pathogen detection, we applied StrainScan to examine the presence of the pathogenic strain of *E. coli* and *M. tuberculosis* in two studies (BioProject Accession: PRJEB1775 and PRJEB2777). The first study is related to the 2011 *E. coli* outbreak in Germany [50], which was caused by an enteroaggregative (EAEC) strain. These datasets are sequenced using metagenomic sequencing from stool samples. According to the original article, the pathogenic strain is *E. coli* O104:H4. The second study investigates the frequency of *M. tuberculosis* relapses within patients from the REMoxTB clinical trial, which evaluated the treatment for *M. tuberculosis* in previously untreated patients [51]. The sequencing data is obtained from bacterial isolates, and each dataset has a publicly available assembled genome representing the strain contained in the sample. From each of these two studies, we selected six samples for the experiment. For comparison, we also applied other tools to detect the pathogenic strains in these real sequencing data. As shown in Table 4, StrainScan was able to identify the correct strains in all tested datasets while other tools failed to detect correct strains in some datasets. Although StrainEst could identify correct strains in most datasets, it only returned the representative strain of the correct strain for some datasets.

**Table 4:**
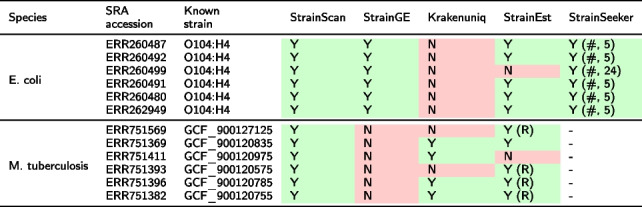
Performance of StrainScan, StrainGE, KrakenUniq, StrainEst, and StrainSeeker for the identification of pathogenic strain of *E. coli* and *M. tuberculosis*. Green: consistent results with the ground truth. Red: inconsistent results with the ground truth. “#”: there are multiple hits with identical scores in the output. “R”: the identified strain is the representative strain of the ground truth. Note that StrainSeeker is not able to identify strains of *M. tuberculosis* and therefore related results are “-”. For StrainSeeker, the number in parentheses represents the number of multiple hits

### StrainScan accurately detects the dynamics of antibiotic-resistant *S. epidermidis* strains

**Fig. 10:**
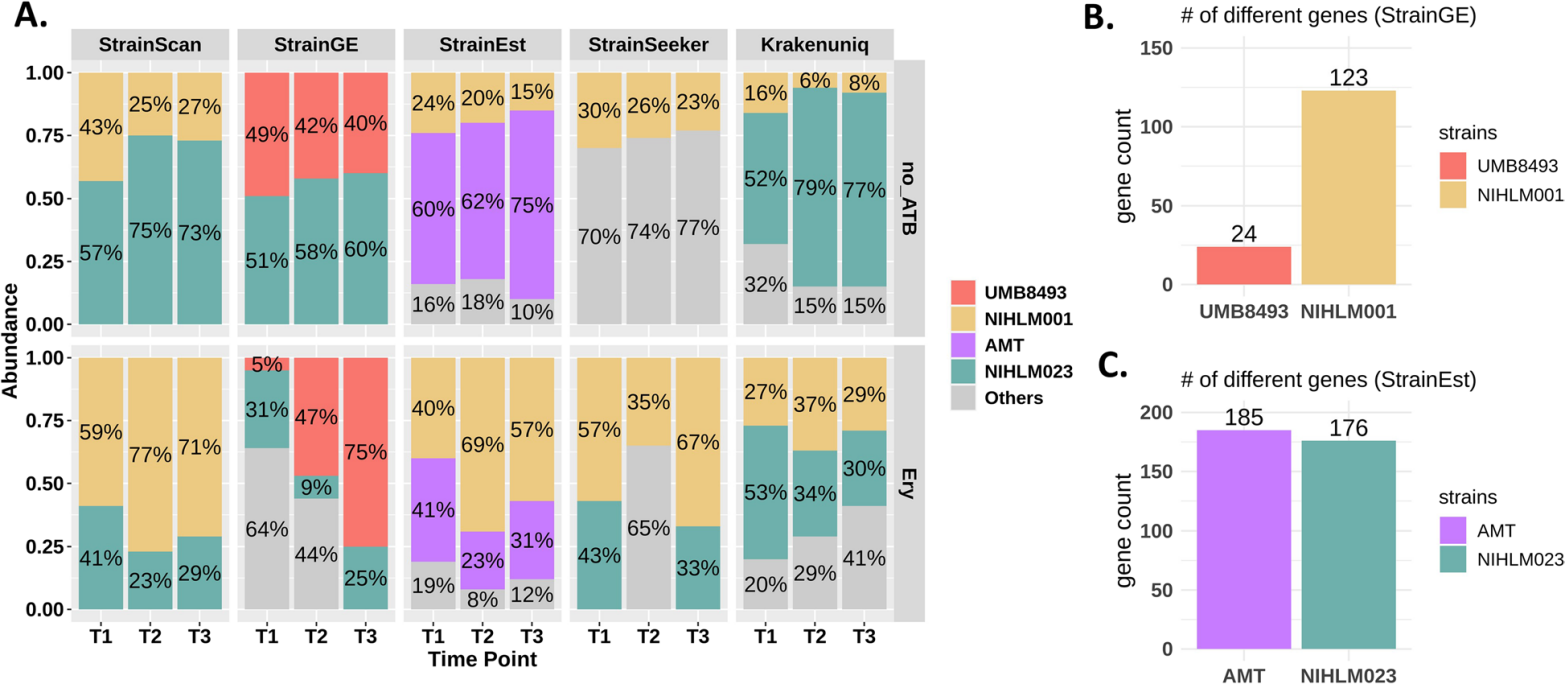
A The estimated abundance of *S. epidermidis* strains by 5 tools in 6 real metagenomic samples. No_ATB: the group without antibiotic treatment, and the non-resistant strain NIHLM023 is the dominant strain. Ery: the group with antibiotic erythromycin treatment, and the resistant strain NIHLM001 is the dominant strain. Each color represents a strain. B-C The number of different genes between the actual strain and the representative strain identified by StrainGE and StrainEst

In this experiment, we tested the ability of StrainScan to detect the dynamics of two *S. epidermidis* strains in public metagenomic datasets (PRJNA490375). According to the original study [52], these datasets are generated from a mixed strain culture in vitro. The authors grew two skin isolates of *S. epidermidis* with 1:1 ratio in two groups. One group was grown with antibiotic erythromycin treatment (Ery) and the other was grown without antibiotic treatment (no_ATB). Among these two isolates, NIHLM023 is not resistant to the antibiotic erythromycin while NIHLM001 is highly resistant to erythromycin. Finally, six datasets were obtained from two groups by metagenomic sequencing at three different time points. Although the relative abundance of these two strains is not given, the coverage ratio at each time point is given, which can be used to evaluate the proportion change of the two strains. According to the coverage ratio reported by the original study, NIHLM023 was always the dominant strain in the group of no_ATB while NIHLM001 was the dominant one in the group of Ery at each time point. Then, we applied StrainScan and other tools to these six datasets, and the result is shown in Fig. 10A. StrainScan is the only tool that returns the correct strain proportion of 2 strains at each time point and has no false-positive identifications in all samples. Among the tested tools, StrainGE and StrainEst returned the correct representative strains in some samples. Thus, we investigated the different genes between these representative strains and the actual strains. The genes are predicted by Prokka [58] and the comparison analysis is done by Roary [59]. As shown in Fig. 10B and C, there are still many different genes between these strains. Among these differential genes, some are very important for strain functions. For example, NIHLM001 has the gene ssaA_1, which has been shown to be associated with many properties of the strain, such as drug resistance [60–62]. But this gene is not possessed by the representative strain UMB8493. Ideally, the strain-level resolution is preferred for more accurate strain-level analysis.

### StrainScan identifies the low-depth virulent *C. difficile* strain from metagenomic data

To further test the ability of StrainScan in identifying low-depth pathogenic strains, we applied StrainScan and other tools to one metagenomic dataset containing the low-depth virulent *Clostridioides difficile* strains [53]. According to the original study, two virulent *C. difficile* strains with low depth ($\sim$1X) were detected in this dataset. There are only 3 mutations reported between these two strains. Another study [63] also detected the same mutations between *C. difficile* strains, suggesting that they were a result of the presence of multiple highly similar strains rather than sequencing errors. Because *C. difficile* is not one of the six targeted bacteria, we first constructed the reference databases for each tool using 102 complete *Clostridioides difficile* genomes downloaded from NCBI RefSeq. StrainScan, StrainGE, Krakenuniq, Pathoscope2, and Sigma were able to detect the strain with about 1X depth (Table 5). However, Krakenuniq, Pathoscope2, and Sigma identified more than 10 strains, indicating the presence of a large number of false positives. Although StrainGE output only one strain, the identified strain did not contain the two genes TcdA and TcdB that the virulent *Clostridioides difficile* should have. StrainScan was the only tool that detected the low-depth virulent strain without any false positives. However, both StrainScan and StrainGE missed the other strain due to their low depth and ultra-high similarities with the dominant strain.

**Table 5:** The identification results of 7 tools on one metagenomic dataset containing the low-depth virulent *Clostridioides difficile* strains. “NA”: missing values. “TcdA” and “TcdB”: two important genes of the virulent *C. difficile* strains. The presence of “TcdA” and “TcdB” is verified using the Carbohydrate-Active enzymes database [64]

Tools	Identified dominant strain	# of identified strains	TcdA	TcdB
StrainScan	GCF_015238635	1	$\checkmark$	$\checkmark$
StrainGE	GCF_002234355	1	X	X
Krakenuniq	GCF_016767015	11	$\checkmark$	$\checkmark$
Pathoscope2	GCF_018885065	26	$\checkmark$	$\checkmark$
Sigma	GCF_002812605	15	$\checkmark$	X
StrainEst	NA	NA	NA	NA
StrainSeeker	NA	NA	NA	NA

**Fig. 11:**
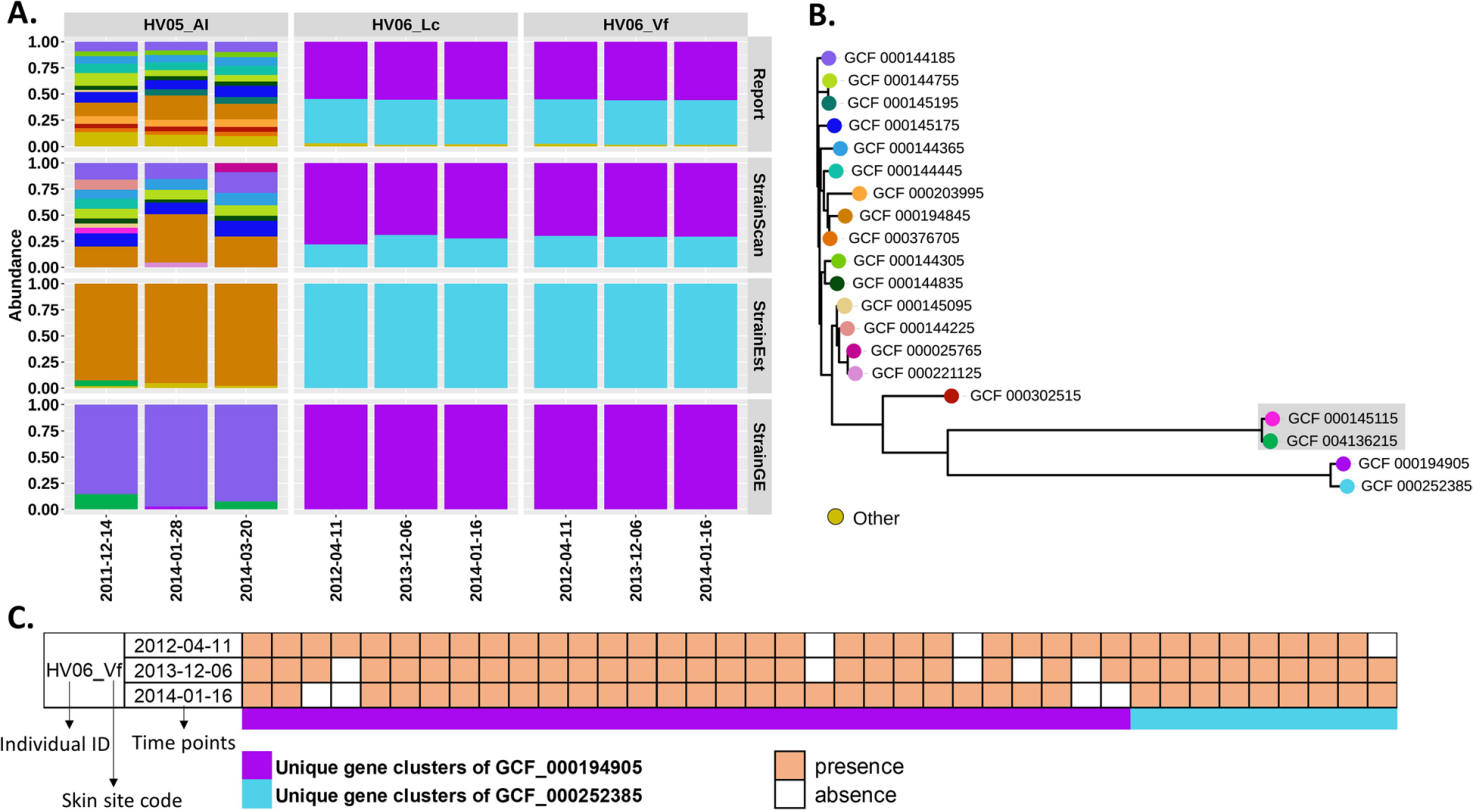
A StrainScan reveals the greater diversity of *C. acnes* in 9 real metagenomic samples. These samples were taken from three different sites of the skin (AI, Lc, and Vf) of two healthy individuals (HV05 and HV06), at different time points. The site codes are described in the original work [15]. B Phylogenetic tree of the identified strains. Leaves are colored using the same schema as in (A) and the distance is the Mash distance [14]. The tree is visualized by iTOL [65]. C The presence of unique gene clusters of “GCF_000194905” and “GCF_000252385”. “GCF_000194905” and “GCF_000252385” are highly similar and are in the same cluster of StrainGE and StrainEst. The presence is given by the original study [15] and each column refers to one unique gene cluster

### StrainScan reveals the greater diversity of *C. acnes* on human skin

Previous studies [11, 15] show that *C. acnes* is one of the most common bacteria on human skin and usually has a complex multi-strain community. StrainEst was applied to re-analyze the human skin data set (SRP002480) from the study [29]. However, we found that in some samples, the number of strains identified by StrainEst was often less than the number of strains reported by the original study. In the original study, the authors used an in-house pipeline to determine the strains and predict their relative abundance in the samples, and the accuracy of this in-house pipeline was previously validated with extensive simulations for human skin microbiome data [11, 15] Therefore, we selected nine samples from two individuals who fit this case and then re-analyzed these samples using StrainEst and StrainScan. Then, we compared their outputs with those reported in the original study. Considering the competitive performance of StrainGE, we also added it into the comparison. For consistency, we selected all strains used in the original study to build the new custom databases for StrainEst, StrainGE, and StrainScan, and used these newly established databases for subsequent analyses. The result is shown in Fig. 11. StrainEst and StrainGE only returned one or two strains in all tested datasets while more strains are reported according to the original study. StrainScan displayed a more similar relative abundance pattern with the reported result than StrainEst and StrainGE. Besides, StrainScan also detected some strains in the samples of “HV05_AI” that were not found in original studies, which implied the greater diversity of *C. acnes*. For example, two highly similar strains “GCF_000145115” and “GCF_004136215” were identified by StrainScan and StrainGE/StrainEst, respectively. As shown in the gray box in Fig. 11B, they are highly close in the tree, and “GCF_004136215” is the representative strain of “GCF_000145115” for StrainGE and StrainEst. However, they have 76 different genes. According to the SNP analysis of original study [11], 22 unique SNPs of “GCF_000145115” were identified in the sample “HV05_AI” on “2011-12-14” while no unique SNPs of “GCF_004136215” were detected. Thus, it is more likely that “GCF_000145115” rather than “GCF_004136215” is present in the sample, which shows the advantage of the high resolution of StrainScan.

In addition, StrainGE and StrainEst identified different representative strains for the same sample “HV06” (Fig. 11A). This result shows that the identification results of cluster-based methods can be influenced by different clustering and representative strain selection strategies. In contrast, StrainScan identifies two highly similar strains, “GCF_000194905” and “GCF_000252385” in these samples, which are consistent with the reported result. The different strain composition results can affect the downstream analysis. We took the analysis result of “HV06_Vf” as one example here. By aligning reads to unique gene clusters of “GCF_000194905” and “GCF_000252385,” the original study analyzed the gene content change of these 2 strains in “HV06_Vf” over time. As shown in Fig. 11C, the presence of the unique gene clusters from two strains varied at different time points, which reflected the strain-level functional variation in this individual. This result shows that StrainScan distinguishes highly similar strains, which can be used together for more comprehensive strain-level functional analysis.

### Two examples showing more applications of StrainScan on real metagenomic sequencing data

To show the wide application of StrainScan on real metagenomic sequencing data, we applied StrainScan to analyze *E. coli* strains in cross-sectional studies [48, 54] and *P. copri* strains in different populations [9]. The analysis results show that StrainScan can accurately identify strains at a higher resolution from metagenomic samples, which can lead to more comprehensive biological insights. For example, StrainScan is the only tool capable of distinguishing *E. coli* strains in samples from three countries into three distinct groups (Supplementary Fig. S12). Similarly, by analyzing *P. copri* strains identified by StrainScan, we observed that strains from Omnivores samples are clearly separated from Vegans samples in terms of the phylogenetic relationship, while strains from Vegetarians samples lie somewhere in between (Supplementary Fig. S12). Additional details regarding these two experiments can be found in Supplementary Sections 2.4 and 2.5.

## Discussion

In this work, we presented StrainScan, a new strain-level composition analysis tool for short reads. We designed a novel tree-based *k*-mers indexing structure to strike a balance between strain identification accuracy and computational complexity. Then, by applying informative *k*-mers and the elastic net model to identify strains and predict their abundance, StrainScan improved the resolution of the strain-level analysis and the accuracy of abundance estimation.

StrainScan shows higher accuracy and resolution than other tested tools across all benchmark datasets with different complexity. In particular, StrainScan outperforms all other tools on datasets containing strains with higher similarity and various sequencing depths. This level of high resolution can be achieved by alignment-based tools such as Pathoscope2 and Sigma. But StrainScan is at least 10 times faster than them. The experimental results of mock data and real data further demonstrate that StrainScan can provide more comprehensive strain-level composition analysis.

Novel strains that are not in the reference database tend to appear often. While tools like StrainGE and StrainEst return representative strains that are most similar to the present strains in a sample, StrainScan returns a strain that is the closest match. As a result, the returned strains by StrainScan can represent the actual strains with higher accuracy. Additionally, StrainScan offers information on the identified clusters in the output, enabling users to consider the identified cluster(s) as the subject for downstream analysis in cases where the genome sequence of the actual strain is unavailable. As shown in our benchmark experiments (Fig. 9 and Supplementary Fig. S10), StrainScan is able to identify the strain that has a smaller Mash distance to the actual strain in the sample, which can provide more accurate reference strain for the downstream analysis. Besides, as shown in Fig. 11C, highly similar reference strains can still contain strain-specific genes and thus using all of them (rather than a representative) can reveal more comprehensive strain-level functional changes in the comparison of multiple samples. These results indicate that using strains identified by StrainScan for downstream analysis has a great potential to generate new biological insights compared to tools that only return representative strains.

When the strains are highly similar, the sequencing depth is the main factor affecting the resolution of StrainScan. Thus, when the strains have high similarities and low depths, our cluster search using CST will fail to identify some clusters due to the low coverage. As a result, the performance of StrainScan will drop rapidly if the depth of the strain is lower than 1X. Moreover, we investigated the minimum sequencing depth required by StrainScan and the likelihood of detecting a strain in low-depth samples (Supplementary Section 2.6). We tested the limits of StrainScan on building the CST for 25,349 complete and draft *E. coli* genomes. The program requires > 1TB of memory. Our empirical tests show that StrainScan is efficient for building the CST for less than 5000 genomes (see Supplementary Table S12). However, for bacteria with many genomes and high intra-species diversity (e.g., *E. coli*), the construction of CST can take a longer time. Thus, we recommend users to use only complete genomes for constructing the indexing structure if there are more than 5000 genomes. Our first step based on cluster search can efficiently reduce the search space. However, if all the reference genomes are highly similar and only differ by a handful of bases, they tend to be grouped in one cluster. In this case, the cluster search still returns a large search space for the second step, which does not take full advantage of the cluster indexing structure. *M. tuberculosis* has a large cluster, which slows down the strain identification. It should be noted that the quality of the reference genomes can affect reference-based methods [43] including StrainScan. StrainScan allows users to build the database with their own genomes. Thus, pre-processing can be conducted to mitigate data contamination or bias problems in the input genomes.

As discussed above, the scalability of input reference genomes can be a limitation of StrainScan. However, many efficient data structures [66] or methods [12] have been developed to increase the scalability of microbiome strain-level analysis. For example, one tool called mSWEEP [67] can identify strain lineages accurately from large-scale reference genomes by utilizing pseudoalignment technology. Thus, for future work, we plan to extend pseudoalignment-based methods or efficient data structures such as HyperLogLog [30] to increase the scalability of StrainScan, and speed up both the database construction and identification process of StrainScan.

## Conclusions

In conclusion, we developed an accurate, efficient, and high-resolution strain-level composition analysis tool for short reads. The experiment results on simulated and mock datasets show that StrainScan can achieve more accurate strain-level microbiome composition analysis than existing tools while keeping the high resolution. The results of these benchmark experiments also prove StrainScan’s robustness with complex samples, low-abundance strains, and strains that are not in the reference database. Furthermore, the analysis results of real metagenomic datasets show that StrainScan can benefit pathogenic strain identification, strain-level composition and functional analysis, and meta-analysis across different studies or datasets. Based on these results, we believe that StrainScan is an important contribution to the field and offers improved performance over state-of-the-art tools.

### Supplementary Information

**Additional file 1.** Supplementary sections, figures, and tables. Supplementary information is contained in the additional PDF file.

## Data Availability

All data and codes used for this study are available online. The source code of StrainScan is freely available at https://github.com/liaoherui/StrainScan.
