# Supplementary material for: High-resolution strain-level microbiome composition analysis from short reads
Source: Microbiome. 2023 Aug 17;11:183. doi: 10.1186/s40168-023-01615-w (PMC10433603; doi:10.1186/s40168-023-01615-w)
Supplement: Supplementary file 2 — Additional file 1. Supplementary sections, figures, and tables. Supplementary information is contained in the additional PDF file. [file 40168_2023_1615_MOESM1_ESM.pdf]

Supplementary information for "High-resolution strain-level microbiome composition analysis from short reads"

Herui Liao, Yongxin Ji, and Yanni Sun

Department of Electrical Engineering, City University of Hong Kong, Kowloon, Hong Kong SAR

July 20, 2023

1 Supplementary Methods

1.1 CST optimization

The main task of the optimization process is to reconstruct all small k-mer sets. In the CST, if a node has a large k-mer set, it is called a *strong node*. And the nodes with small k-mer sets are called *weak nodes*. To distinguish strong and weak nodes, we need to set a specific cutoff of the k-mer number.

Ideally, we assume that reads are randomly distributed across a genome. Thus, the count of each k-mer follows a Poisson distribution with mean λ equal to the average coverage across the whole genome [1]. When a node only possesses a few k-mers, its k-mer count distribution will not reflect the coverage of the strains in a sample. This not only causes a high FP rate but also reduces the accuracy of strain abundance estimation. Thus, we require that each node has enough k-mers so that its k-mer counts still exhibit a Poisson distribution with a mean roughly equal to λ. If these k-mers are independently distributed, estimating the minimum k-mer number cutoff of the reliable k-mer set would be easy. When the k-mer number reaches 1,000, the KS distance decreases very slowly and approaches 0.02 (Supplementary Figure S2). Therefore, we set the k-mer number cutoff as 1,000 in this work.

Once we determine the cutoff, we can identify all weak nodes with k-mer numbers below the cutoff. A key observation behind the weak node augmentation is that we adopt Breadth-first Search (BFS) for the CST traversal and thus only need to choose among nodes at the same depth. As long as we can ensure that the nodes at the same depth have no overlapping k-mers, we can conduct the search without any ambiguity. This means that we can tolerate some non-unique k-mers in the nodes of CST. When we traverse to a node v at depth d, any leaf node i at a depth smaller than d has been examined, and thus k-mers from L_i might be reused by node v. If v is a weak node, we can change it into a strong node using this method. Specifically, we employ the set operations of k-mers to reconstruct every weak node:

$$\begin{aligned} K'_v &= K_v + (\bigcup_{i \in SUB_v} L_i - \bigcup_{i \in EXP_v} L_i) \cap \bigcup_{i \in PRE_v} L_i \\ &= \bigcap_{i \in SUB_v} L_i - \bigcup_{i \in EXT_v} L_i \end{aligned} \quad (1)$$

where $\mathbf{K'_v}$ is the reconstructed k-mer set for the given weak node v. Compared with equation (1) in the main text, a part of k-mers shared between PRE_v and SUB_v can be added to v's k-mer set, thereby achieving the weak node augmentation.

After implementing the reconstruction method, most of the weak nodes will become strong nodes. For instance, in the optimized CST built from 112 P. copri strains, only three out of 101 nodes are weak using our method. In order to decide the presence of a strain in a sample with high precision, we require all leaf nodes to be strong nodes. If the above procedure still leaves weak leaf nodes, we will combine them with their sibling nodes so that enough k-mers can be derived from the combined node. This process will iterate until all leaf nodes become strong nodes.

1.2 Adjusting $\mathrm{C_v}$ for accurate abundance estimation

A special case may occur in the cluster search process. When the sequencing data contains multiple strains in different clusters, some overlapping *k*-mers introduced during the CST optimization will cause FPs for the nodes' *k*-mer matches. Specifically, suppose we reach a reconstructed node v after a leaf r has been identified to be present with an estimated abundance A_r. In this case, the match counts of the *k*-mers in $K_v \cap L_r$ are the sum of *k*-mer match counts from both r and strains in T_v. Using the estimated abundance A_r, we can estimate the match counts of $K_v \cap L_r$ originated from r based on the Poisson distribution with $\lambda = A_r$:

$$P(c = k) = \frac{A_r^k \cdot e^{-A_r}}{k!} \tag{2}$$

The estimated *k*-mer match vector from r is denoted as $\tilde{C}_r$ (the size is $|K_v \cap L_r|$). Then, both of the vectors C_v and $\tilde{C}_r$ are sorted on the basis of increasing value of *k*-mer match count c_i, and we adjust C_v by $C_v = C_v - \tilde{C}_r$. This method can be applied to multiple identified leaf nodes at different depths. After adjusting C_v for all identified leaf nodes, we can use C_v to calculate scoring metrics without any ambiguity.

1.3 The *k*-mer matrix construction

The pseudocode of constructing the k-mer matrix is given in Algorithm 1. To speed up strain search in the same cluster, we use a two-dimensional hash table H, which keeps track of the matched *k*-mers of each strain. H has size of m by n, where m is the number of *k*-mers in the cluster and n is the number of strains in the cluster. Each cell $H[i][j]$ (0 or 1) represents whether the ith *k*-mer is present in the jth strain. By using H, the algorithm can easily find strain-specific *k*-mers. Then, the algorithm divides the blocks obtained by Sibeliaz [2] into two parts. The first part is the blocks owned by some strains, which are SVs. The second part is the blocks that are shared by all strains, which are used to find joint *k*-mers. For *k*-mers from the second part, we use H to keep all eligible *k*-mers as a way to obtain all joint *k*-mers. An eligible *k*-mer here means that the strains with this *k*-mer are a subset of all strains in the block.

Algorithm 1 Build the *k*-mer matrix for strains in the same cluster.

Require: The strain genome set R = $\{R_1, R_2, \ldots, R_N\}$ with N strains. Collinear blocks B of R generated by Sibeliaz.
Define an empty sparse matrix X. ▷ The output matrix
Define a two-dimensional hash table H for recording the strain label of all *k*-mers
Define a hash table S for recording all output *k*-mers
for R_1 to R_N **do** ▷ Initalize H
    for all *k*-mers $x \in R_i$ ($i = 1$ to N) **do**
        $H[x][R_i] = Null$, $H[revcomp(x)][R_i] = Null$ ▷ $revcomp$ returns the reverse compelement of a dna sequence.
for all *k*-mers $x \in H$ **do** ▷ Find strain-specific *k*-mers of each strain
    if $len(H[x]) == 1$ **then**
        $S[x] = Null$, $S[revcomp(x)] = Null$
for all blocks $b \in B$ **do**
    for all *k*-mers $x \in b$ **do**
        if not $len(b) == N$ **then** ▷ Find group-specific *k*-mers from blocks
            if $len(H[x]) == len(b)$ **then**
                $S[x] = Null$, $S[revcomp(x)] = Null$
        else ▷ Find joint *k*-mers from blocks
            if $H(x) \subset R$ **then**
                $S[x] = Null$, $S[revcomp(x)] = Null$
Initialize X as a $N \times M$ matrix, $M = len(S)$
for all *k*-mers $x \in S$ **do** ▷ Fill out the *k*-mer matrix
    for R_1 to R_N **do**
        if R_i ($i = 1$ to N) $\in H[x]$ **then**
            $X[x, R_i] = 1$
        else
            $X[x, R_i] = 0$

2 Supplementary Experiments

2.1 Low-depth experiments for multiple strains

To test the ability of different tools on identifying multiple strains at a lower depth, we simulated more short reads from previously selected 2 strains using different coverage profiles. Each dataset contains one dominant strain (10X) and one minor strain with 10X, 5X, 3X and 1X depths, respectively. Thus, there were 80 datasets for each bacterium, 40 containing strains from different clusters and 40 containing strains from the same cluster. As a result, we generated a total of 480 (80x6) datasets for the six bacteria. Then, all tools were applied to identify strains in these datasets. The recall, precision, and F1 score are shown in Supplementary Table S7 and S8. StrainScan shows good robustness in identifying strains that are from different clusters and outperforms other tools. In particular, StrainScan shows a clear advantage in identifying low-depth strains from many highly similar strains. For example, when identifying 10X and 1X *C. acnes* strains that are from different clusters, StrainScan has 0.98 F1 score, while Krakenuniq and StrainGE, in the second and third place, have only 0.88 and 0.81 F1 scores. However, when the strains are from the same cluster and the minor strain has only 1X depth, StrainScan tends to miss the minor strain. Nevertheless, in this case, StrainScan still shows a higher F1 score for most bacteria at the strain level than other tools. When the depth of minor strain increases to 5X, StrainScan achieves the best F1 score at the strain level for all tested bacteria.

2.2 Detecting two strains without reference genomes in the database

To evaluate different tools' ability to detect multiple strains without reference genomes in the database, we generated twenty simulated datasets with 90 *E. coli* strains used in the main article. Each dataset contains two strains randomly selected from the 90 strains. These two strains have 100X and 10X depths, respectively. Then, we applied all tools to identify the strain in each dataset and recorded the result. Here, we considered the closest matched strains of two actual strains in the database as the ground truth. The F1 score of all tools and the mash distance of identified strains to the ground truth were shown in Supplementary Figure S10. The results show that StrainScan achieves a 95% F1 score, while the second-best tool StrainGE (cluster-level) only achieved a 78% F1 score. In addition, StrainScan has no false positive identifications in all tested datasets, and the strains identified by StrainScan have a smaller mash distance to the ground truth than other tools (Supplementary Table S10).

2.3 Assessment of StrainScan on spiked metagenomic sequences

In this experiment, we evaluate whether Strainscan keeps the same performance on metagenomic data, which contains reads from different species. We used spiked metagenomic data in this experiment. Considering that *P. copri* and *E. coli* are two very common species in the human gut and their reference genomes differ significantly, we decided to take these two as the target species in this experiment. Then, we collected two real human gut metagenomic samples devoid of *P. copri* and *E. coli*, respectively (SRR769529 and SRR341648) according to the provided species-level analysis at NCBI SRA. Furthermore, we also applied all tested tools to these two samples to detect *P. copri* in SRR769529 or *E. coli* in SRR341648. None of the tested tools can identify any strains of *P. copri* or *E. coli* in the corresponding datasets. After the data validation, we mixed all simulated "single-strain" and 10 simulated "multiple-strain" datasets from *P. copri* and *E. coli* with the two metagenomic datasets, respectively. As a result, there are 130 mixed datasets, which contain the strains of the target species (*E. coli* and *P. copri*) and many other species. We applied StrainScan to these mixed datasets and compared its performance with the original result (see Supplementary Table S10). The comparison shows that StrainScan has the same results on these spiked metagenomic datasets as on the simulated whole genome sequencing data, demonstrating the robustness of StrainScan on complex samples.

2.4 Analysis of real metagenomic sequencing data in cross-sectional studies

Two studies [3, 4] have demonstrated a correlation between the distribution of *E. coli* strains and the geographical location of their hosts. StrainScan was applied to derive the correlation using datasets from different studies. To show this utility, we applied StrainScan to determine the strain distribution of *E. coli* in several recent studies. We have analyzed two sets of metagenomic and one set of whole genomic sequencing sample from stool or blood samples of different studies, with one dataset including 6 infants in Estonia [5], the second including 6 Chinese adults [6], and the third including 6 USA adults (PRJNA278886). Considering that these samples are from different studies and countries, we only keep the dominant strain of each analyzed sample. We also applied other tools to these samples

(Supplementary Figure S11). StrainScan is the only tool that distinguishes *E. coli* strains into three distinct groups (Supplementary Figure S11B, S11F). According to the phylogenetic tree analysis using Prokka [7] and Roary [8], these identified strains also fall into three distinct clades (Supplementary Figure S11B). The result of StrainSeeker shows a highly similar pattern to StrainScan while it also outputs a large number of strains for each sample, which may include a lot of false positives. The results of the remaining tools differed significantly from each other, making it difficult to draw conclusions. It is worth mentioning that both StrainGE and StrainEst only returned the same representative strain for all 6 USA samples, while the other tools could distinguish different strains with high similarities from these samples. This result indicates that cluster-based tools such as StrainGE and StrainEst suffer from low resolution for the identification of highly similar strains.

2.5 Investigation of strain diversity of *P. copri* in different populations

The evolution of some host-associated microbes can be linked to factors such as host diet. For example, one recent study shows that the host diet and the distinct genetics of human intestinal *P. copri* strains are closely related [9]. However, the original study mainly explored the diversity of strains within different populations from a functional perspective, and not from a taxonomic perspective. Thus, we applied StrainScan to re-analyze 18 human gut metagenomic datasets that are from the same study and have high abundance of *P.copri*. As mentioned earlier, these samples were all taken from the study investigating diet and strain diversity [9]. According to the original study, these samples can be divided into three groups: Omnivores (O), Vegetarians (VG), and Vegans (V). We selected six samples from each group, all six of which have a high abundance of *P. copri* strains. Then, we applied StrainScan to these samples that contain multiple strains of *P. copri* and selected the strains with relative abundance > 0.02 for further analysis. The relative abundance profile of each sample is shown in Supplementary Figure S11E. In Supplementary Figure S11E, we can find some strains are specific to one group, and therefore we label these strains as "specific" strains of that group. These "specific" strains are then analyzed using the principal coordinates analysis (PCoA) (Supplementary Figure S11C) and the phylogenetic tree analysis (Supplementary Figure S11D). The analysis result shows that "V-specific" strains are clearly separated from "O-specific" strains. Interestingly, "VG-specific" strains lie somewhere in between, which is consistent with the conclusion of the original study and our general knowledge. Also, we applied other tools to these samples to compare their performance with StrainScan, and the result is shown in Supplementary Figure S11G. Among these tools, the result of StrainGE shows a highly similar pattern to StrainScan. However, StrainScan identifies more strains with high similarity than StrainGE, which indicates the higher resolution of StrainScan. For the results of the remaining tools, it's hard to get a consistent conclusion as the original study.

2.6 Investigation of the minimum sequencing depth required by StrainScan and the probability of detecting a strain in low-depth samples

To investigate the lower bound of the sequencing depth for the standard CST search algorithm, we used the same 60 *E. coli* strains as the main article (Section "Detecting a reference strain from simulated reads") and simulated 360 low-depth reads for each of the six depths, 1X, 0.5X, 0.4X, 0.3X, 0.2X, and 0.1X. The performance of StrainScan on the six groups of low-depth reads is shown in Supplementary Figure 13. We can observe that StrainScan achieves a recall higher than 0.8 when the sequencing depth is larger than 0.5X. Thus, we consider 0.5X as the lower bound for the standard CST search algorithm of StrainScan.

However, the number of *k*-mers contained in each node can be used to derive a probability for the identification of potential low-depth strains. Thus, we developed a new cluster detection algorithm specially for low-depth strains with depth <1X. Specifically, given the input sequencing data, we first conduct fast *k*-mer match for all short reads against all *k*-mers from the CST. Second, we will calculate $frac_v$ for all nodes based on the mapped *k*-mer match count vectors. Then, instead of performing the Breadth-first Search (BFS) based CST search, we will calculate a probability P_{pathi} for every RTL path $Path_i$ ($\{r,\ i\_node_1, i\_node_2, \ldots, l_i\}$), where r is the root node, i_node_1 is an internal node, and l_i is the leaf node on the path $Path_i$):

$$P_{pathi} = \prod_{v \in Path_i} \sqrt[|Path_i|]{S_v} \tag{3}$$

Where $|Path_i|$ is the node number of P_{pathi}. Taking the $|Path_i|$th root is to normalize the probability P_{pathi}. S_v is the score of the node v calculated with $frac_v$:

$$S_v = \begin{cases} 1 & frac_v > 0.1 \\ log_{10}\left(90 \times frac_v + 1\right) & frac_v \leq 0.1 \end{cases} \tag{4}$$

P_{pathi} reflects the probability that the leaf node (cluster) i contains the low-depth target strain. Finally, we will output the ranking of the clusters with positive probability. In order to evaluate the performance of this algorithm, we simulated reads with the same manner in Figure 1 (the 60 *E. coli* strains) with two very low depths, 0.1X and 0.2X. For each output ranking, we select the cluster with the largest probability as the identification result. To summarize, the algorithm correctly identified 55 out of 60 low-depth data with 0.2X (recall of 91.67%), and 40 out of 60 data with 0.1X (recall of 66.67%). The experimental results showed that the new algorithm can identify most of the low-depth sequencing data with a lower bound of 0.2X. This algorithm has been implemented as a function of StrainScan, and users can make their own choice to use this function on low-depth sequencing data.

3 Supplementary Figures

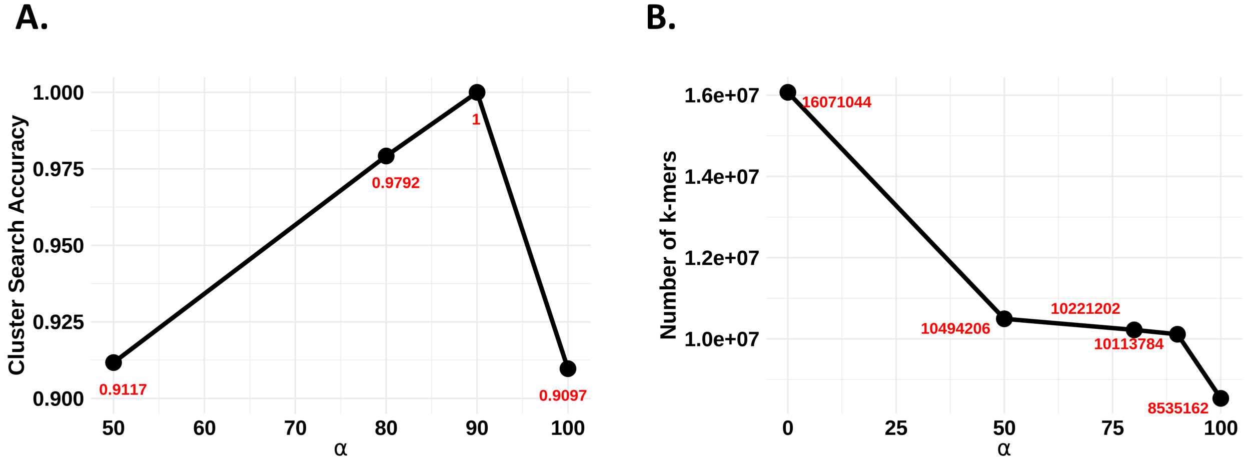

Supplementary Figure S1. (A). The cluster search accuracy when α is 50, 80, and 100, respectively. We generate 1,008 "single-strain" simulated datasets of *S. epidermidis* to test the cluster search performance. The experiments show that the performance is the best when $\alpha = 90$. When we only use k-mers that must occur in all strains ($\alpha = 100$), the resolution of the cluster search decreases. (B). The numbers of selected k-mers using different α values from a relatively large cluster that contains 201 *E.coli* strains.

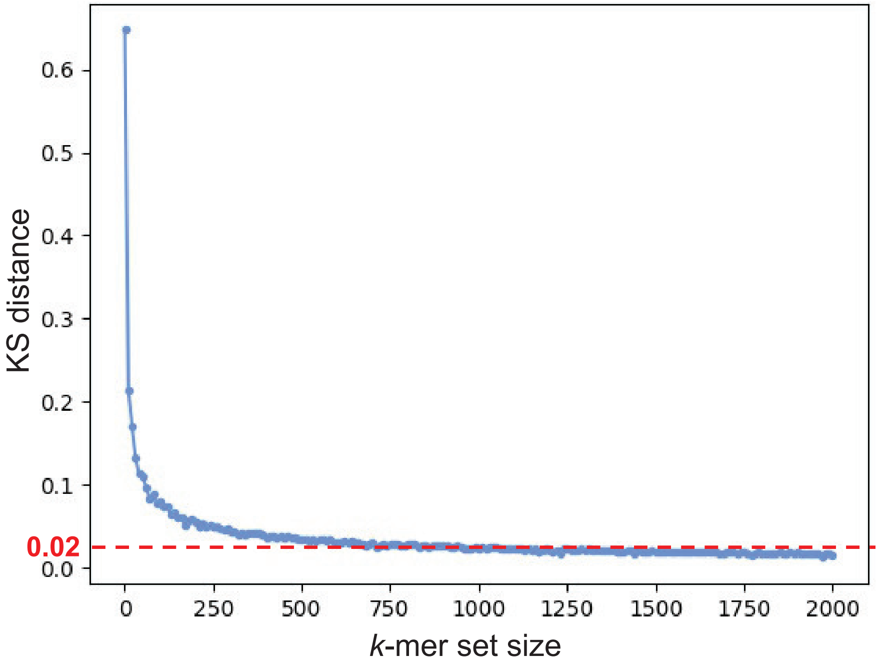

Supplementary Figure S2. The change of the KS distance between the k-mer count distributions on a k-mer set and the k-mers of the whole genome with the k-mer set size. The k-mer set's sizes range from 1 to 2,000 (X-axis). The k-mer set is randomly sampled from the genome multiple times. And the KS distances are the average values from these repeated experiments. The coverage of the simulated reads is 10x. When the KS distance is less than 0.02, there is no significant difference between the two distributions.

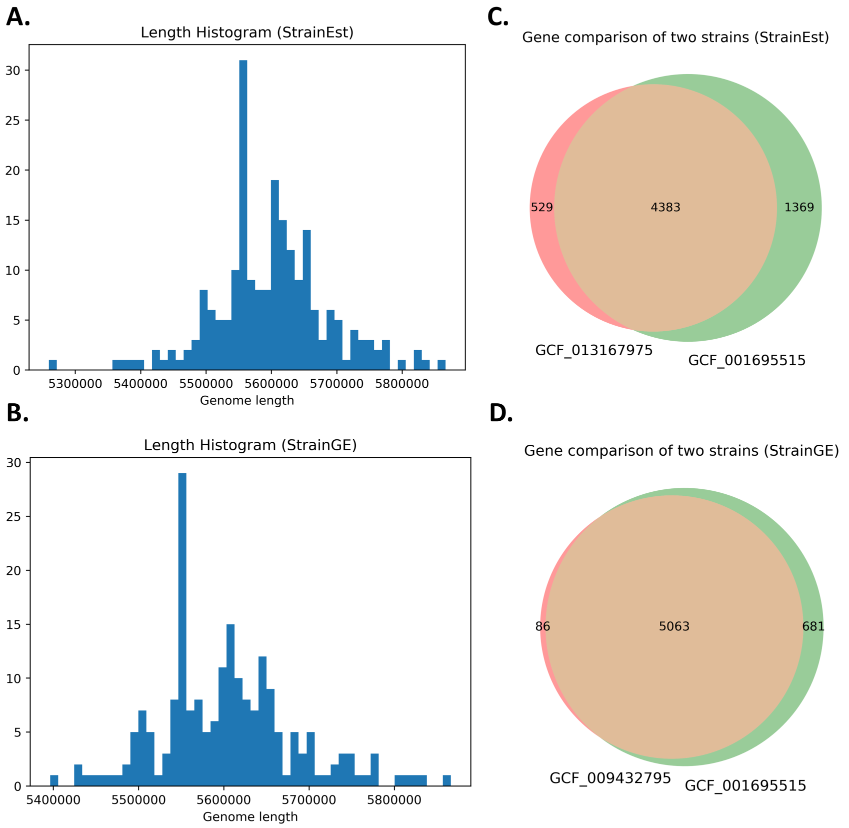

Supplementary Figure S3. A-B. The genome length histogram for the strains in the largest clusters of StrainEst's and StrainGE's *E. coli* database. C-D. Comparison of genes between the longest and shortest strains in the largest clusters of StrainEst's and StrainGE's *E. coli* database. The genes are predicted by Prokka [7] and the comparison analysis is conducted by Roary [8].

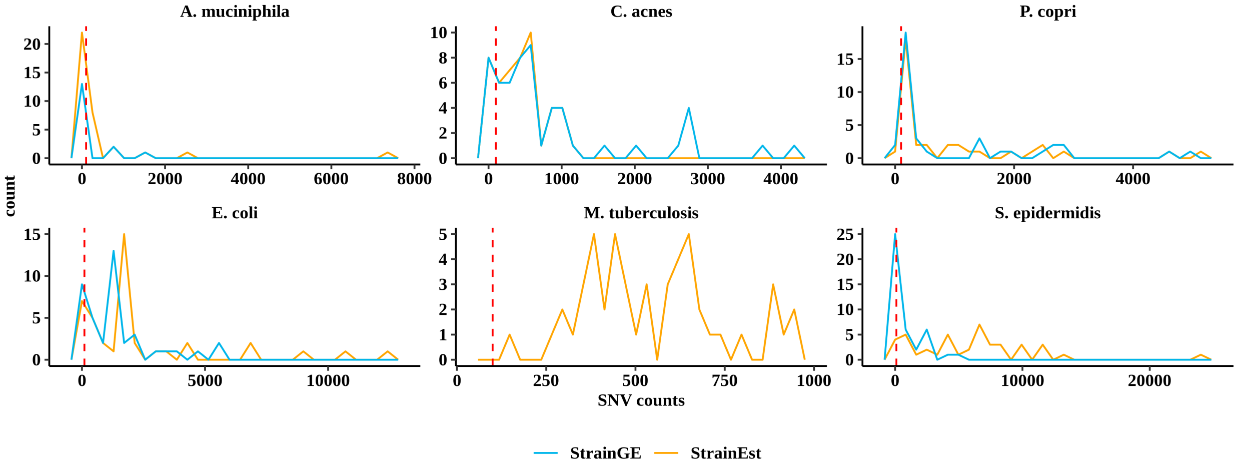

Supplementary Figure S4. The histogram of different SNVs between the representative strains and the actual strains identified by StrainGE and StrainEst in the simulated "single-strain" datasets. The SNVs are detected by MUMmer [10]. The red line in the figure refers to 100 SNVs between the representative strain and the acutual strain.

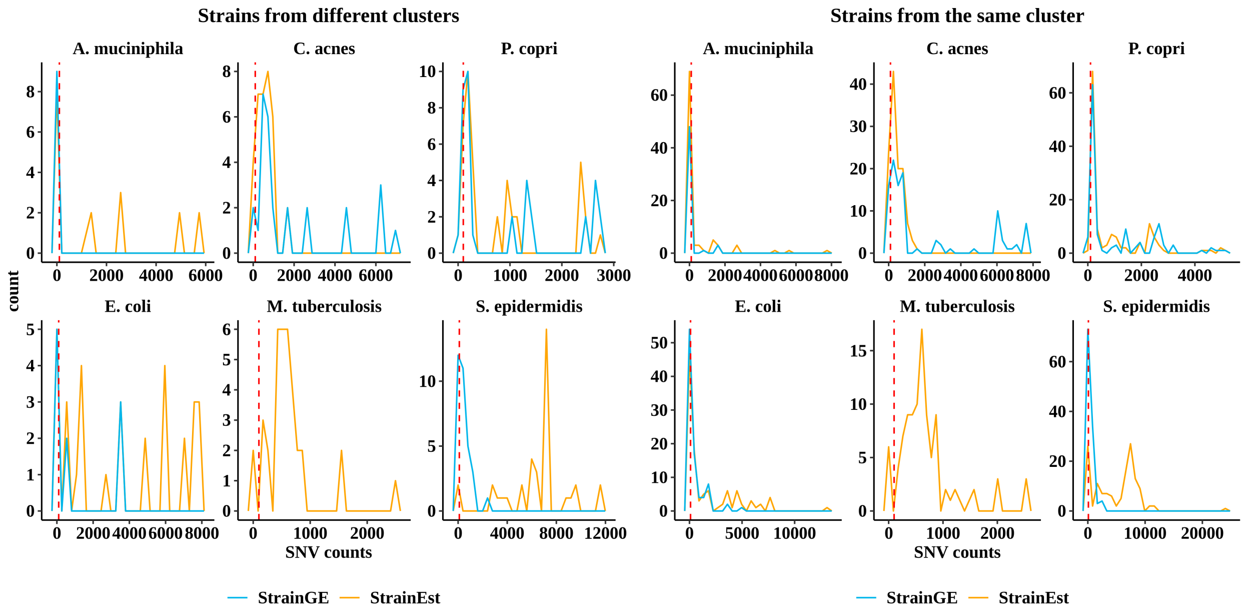

Supplementary Figure S5. The histogram of different SNVs between the representative strains and the actual strains identified by StrainGE and StrainEst in the simulated "multiple-strain" datasets. The SNVs are detected by MUMmer [10]. The red line in the figure refers to 100 SNVs between the representative strain and the acutual strain.

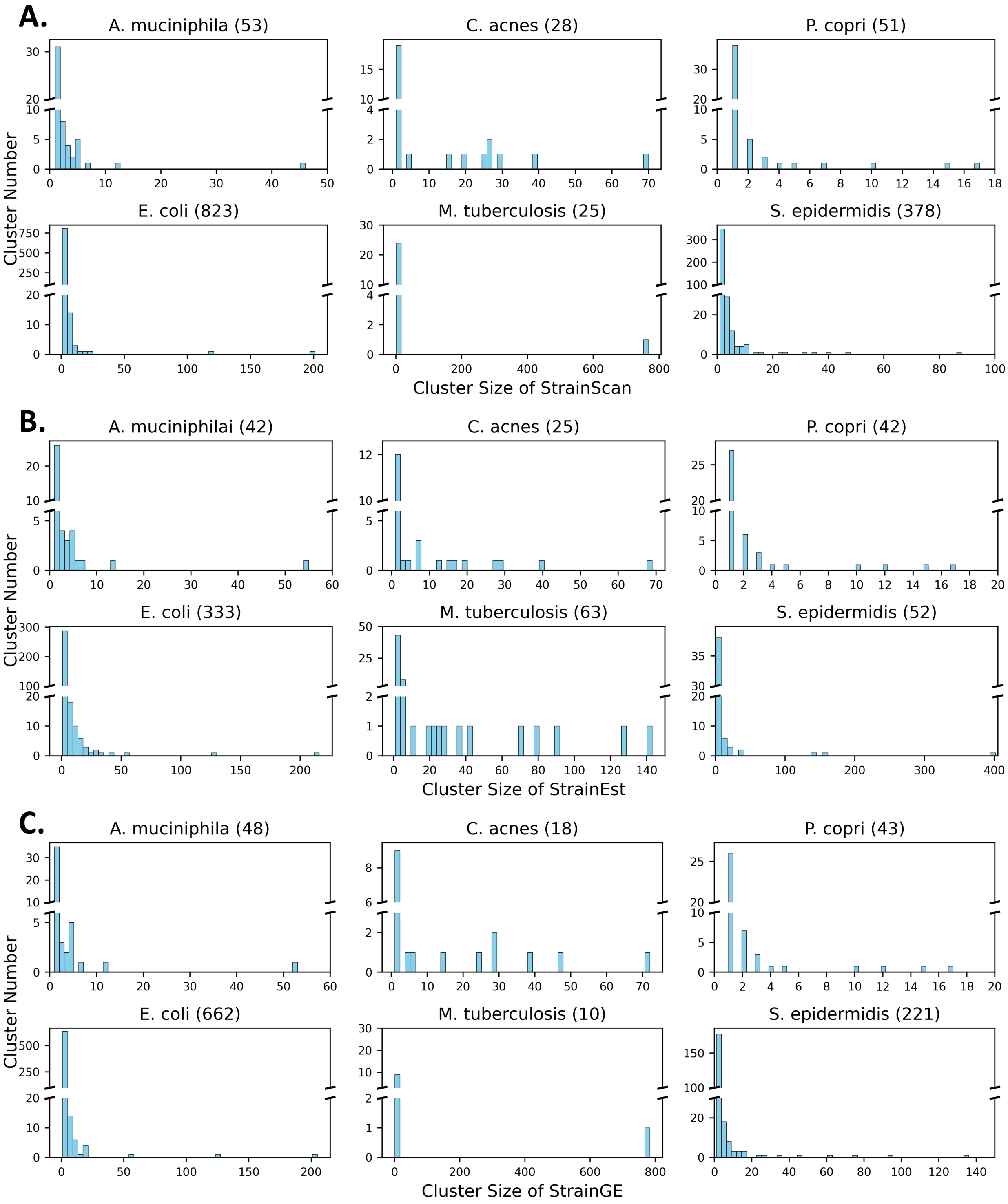

Supplementary Figure S6. (A). The cluster size histogram for clusters in the CST (leaf nodes) of StrainScan. The number in the blank represents the total number of clusters. (B-C). The cluster size histogram for clusters in StrainEst's and StrainGE's reference databases. For each cluster, only one representative strain will be selected and put into the final database for the identification. Although many clusters are small, there are also big ones. Using only the representative strain for the big clusters significantly decreased the resolution of strain identification. The number in the blank represents the total number of clusters.

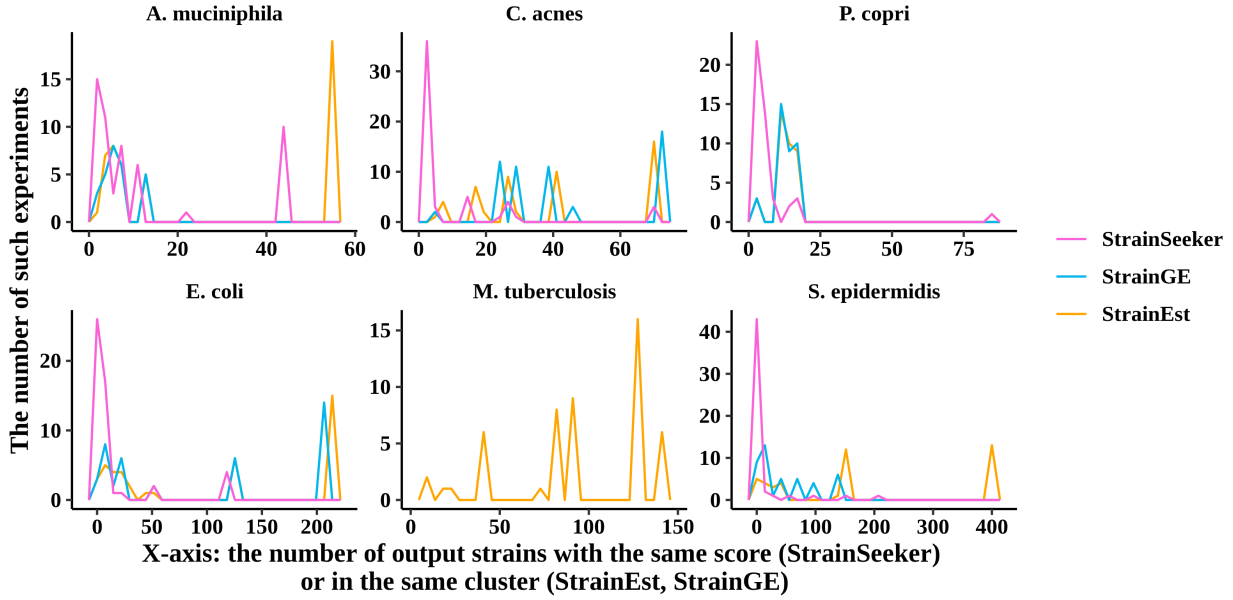

Supplementary Figure S7. The histogram of the identical outputs of three tools. X-axis: the number of output strains with the same score (StrainSeeker) or in the same cluster (StrainEst, StrainGE). Only clusters with at least 2 strains are plotted. Y-axis: the number of such experiments. For example, "50" on the X-axis means that the tool outputs 50 strains with the same score and one of them is correct. Some tools have no results on some species because they have no correct outputs or they returned one strain (StrainSeeker) or single-strain clusters (StrainEst, StrainGE) for all datasets of these species.

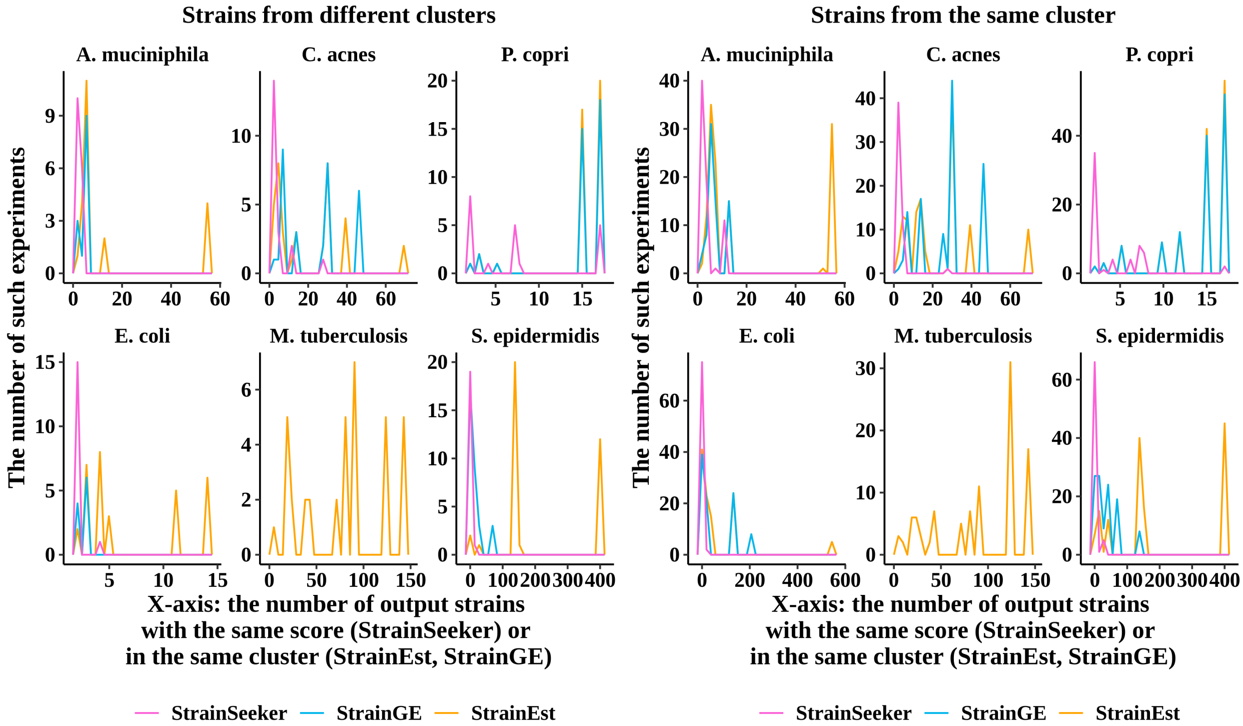

Supplementary Figure S8. The histogram of the identical outputs of three tools. X-axis: the number of output strains with the same score (StrainSeeker) or in the same cluster (StrainEst, StrainGE). Only clusters with at least 2 strains are plotted. Y-axis: the number of such experiments. For example, "50" on the X-axis means that the tool outputs 50 strains with the same score and one of them is correct. Some tools have no results on some species because they have no correct outputs or they returned one strain (StrainSeeker) or single-strain clusters (StrainEst, StrainGE) for all datasets of these species.

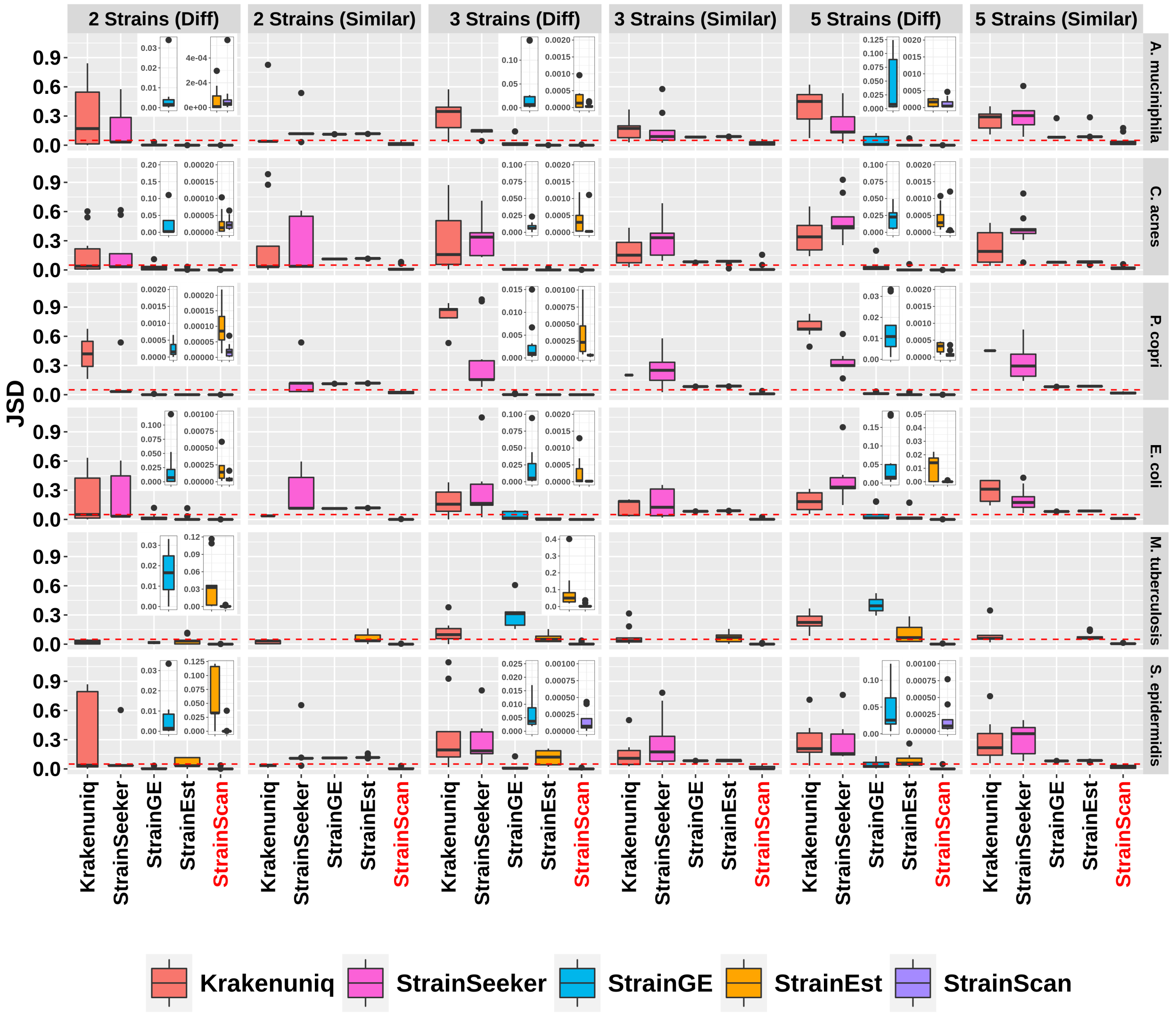

Supplementary Figure S9. The Jensen-Shannon Divergence (JSD) of 5 tools between the ground truth and predicted relative abundance. "Diff" refers to strains from different clusters with k-mer Jaccard similarity $< 95\%$, and "Similar" refers to strains from the same cluster with k-mer Jaccard similarity $\geq 95\%$. The red line in the figure refers to 0.05 of Jensen-Shannon divergence (JSD). The small windows in some of the plots show the JSD distribution for StrainGE, StrainEst, and StrainScan at a much smaller scale.

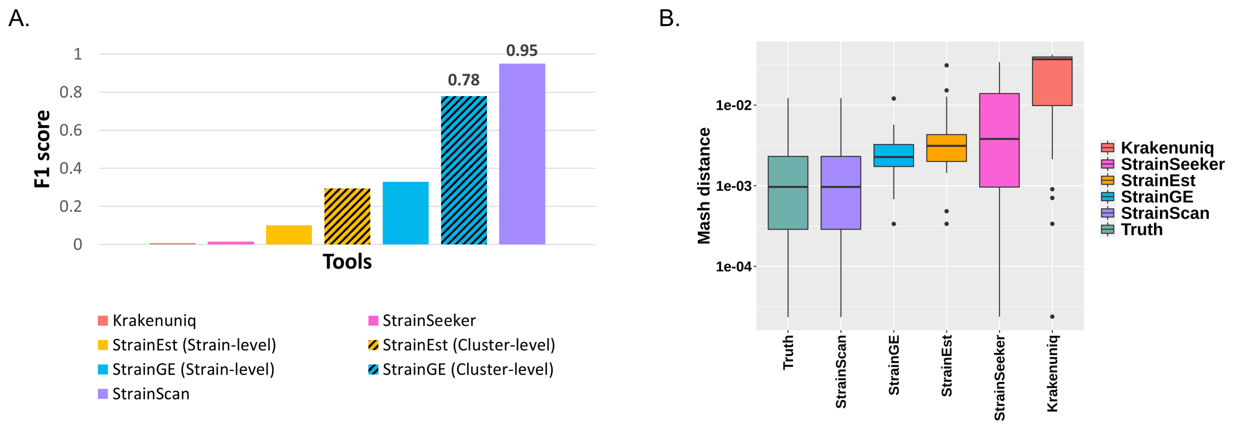

Supplementary Figure S10. (A). Comparison of F1 scores for different tools in identifying two strains without reference genomes in the database across 20 mix-strain simulated datasets. The values on the bar refer to the best and second-best F1 scores. (B). The Mash distance between the identified strains and the ground truth in 20 mix-strain simulated datasets.

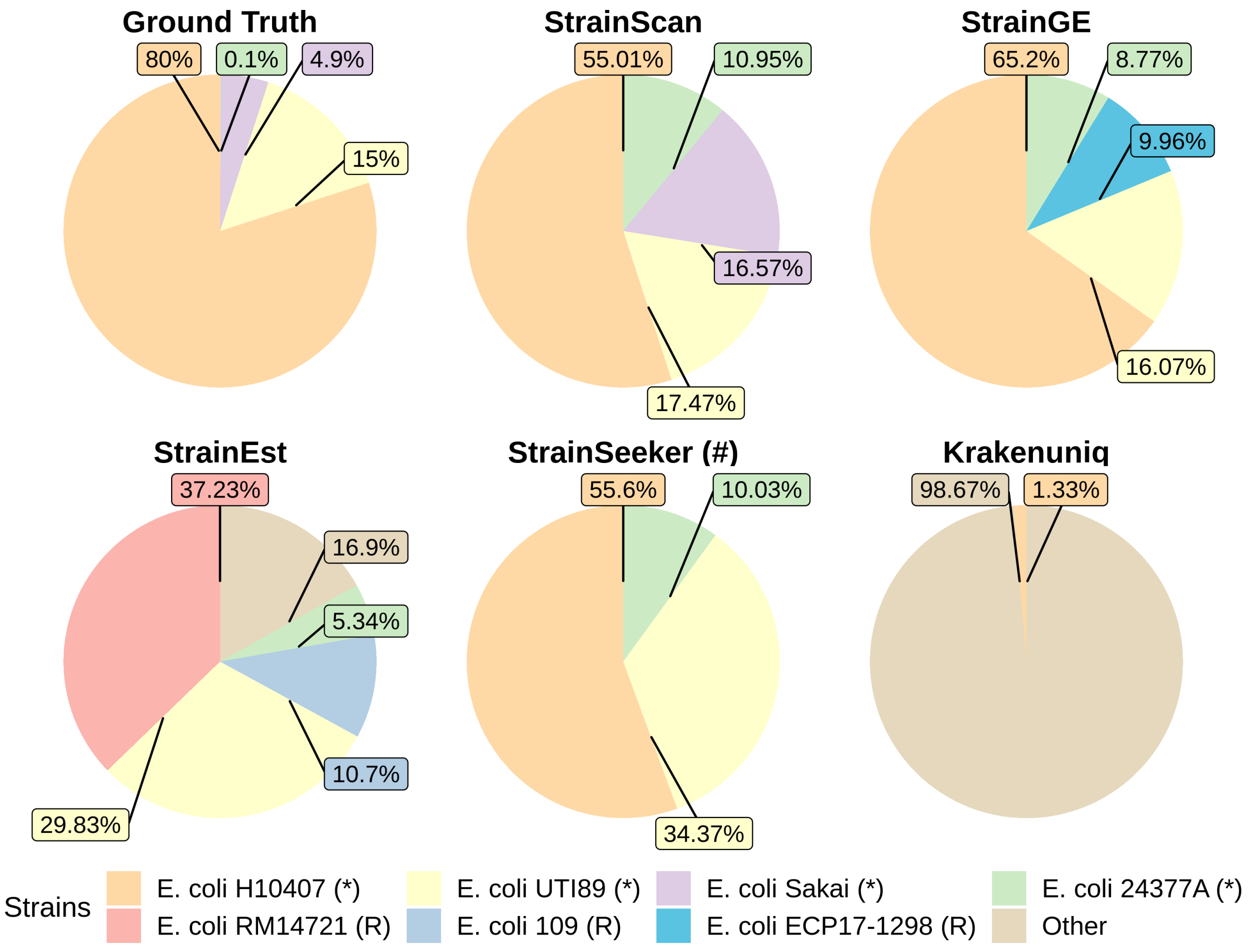

Supplementary Figure S11. Analysis of the mock *E. coli* community data (SRR13355226). #: there are multiple hits with identical scores in the output. *: strains in the ground truth. R: the identified strain is the representative strain of the ground truth. "RM14721" is the representative strain of "H10407" in StrainEst. "ECP17-1298" and "109" are the representative strains of "Sakai" in StrainGE and StrainEst, respectively.

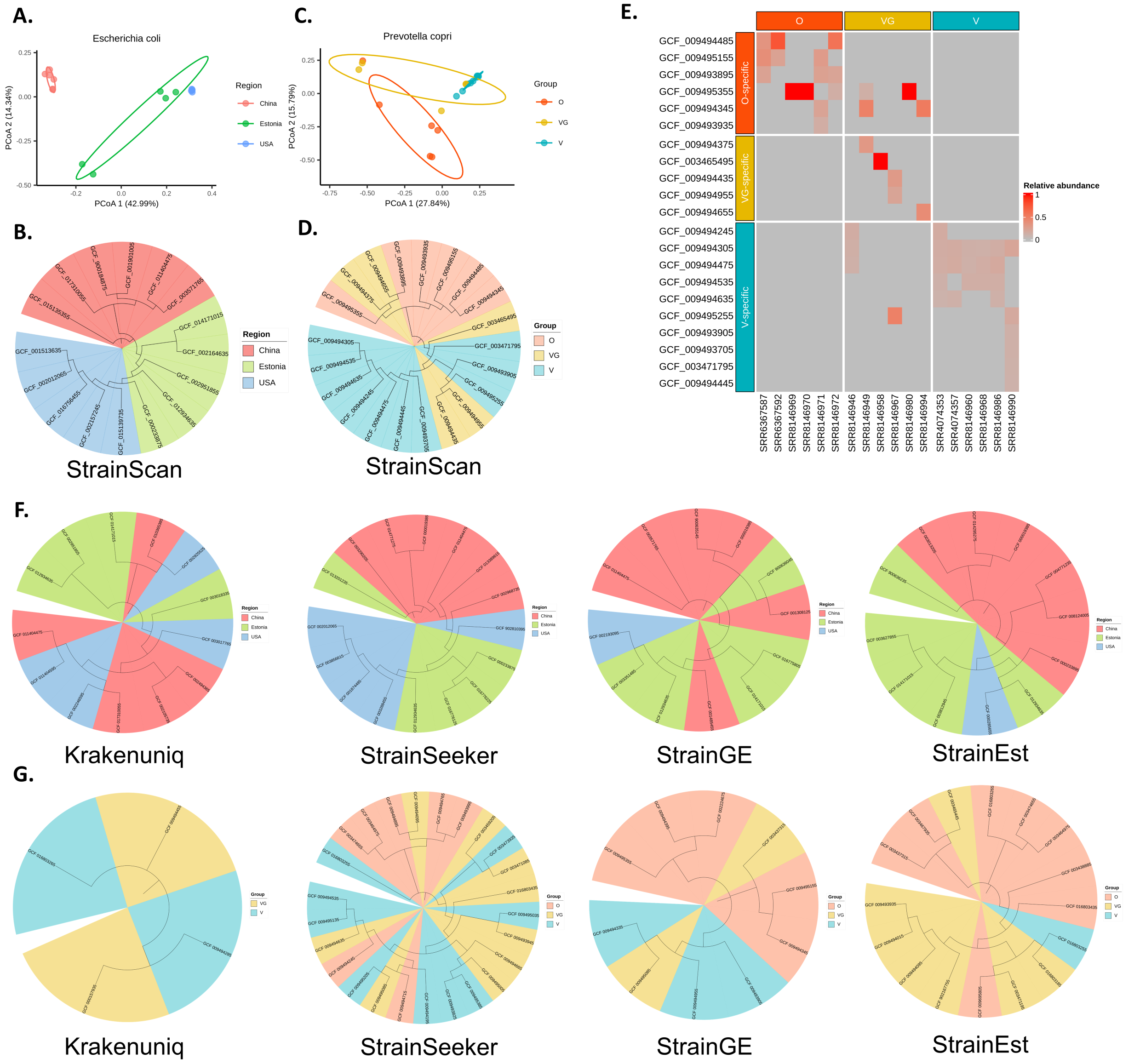

Supplementary Figure S12. (A-B). *E. coli* genomic diversity in the healthy sample of Chinese, Estonian and American cohorts as seen by principal coordinates analysis (PCoA) and the phylogenetic tree analysis of dominant strains. (C-D) *P. copri* genomic diversity in the healthy gut of Omnivores (O), Vegetarians (VG), and Vegans (V) cohorts as seen by principal coordinates analysis (PCoA) and the phylogenetic tree analysis of specific strains. (E). Strain relative abundance profiles of the 21 identified strains in the 18 metagenomic samples from three groups. (F). Identified *E. coli* genome diversity of Krakenuniq, StrainSeeker, StrainGE, and StrainEst from metagenomic samples as seen by the phylogenetic tree analysis of dominant strains. (G). Identified *P. copri* genome diversity of Krakenuniq, StrainSeeker, StrainGE, and StrainEst from metagenomic samples as seen by the phylogenetic tree analysis of specific strains.

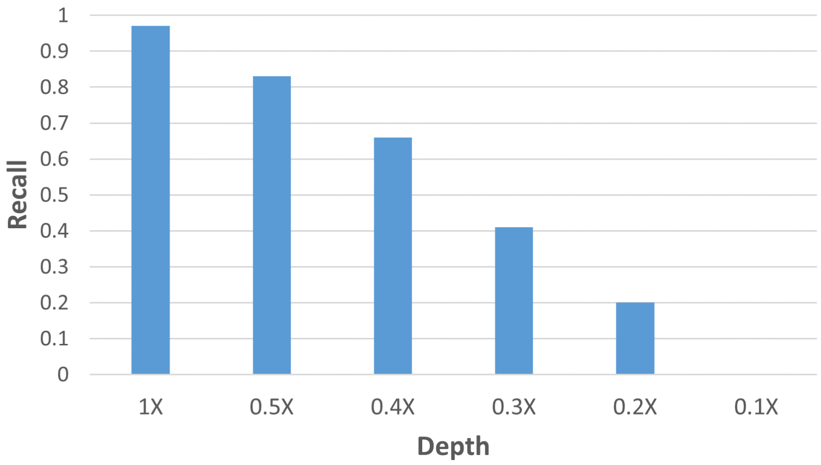

Supplementary Figure S13. The recall of StrainScan on the 360 low-depth reads of *E. coli* with six different sequencing depths.

4 Supplementary Tables

Species	# of strains	Before Clustering			After Clustering		
		# of *k*-mers	# of unique *k*-mers	# of strains without unique *k*-mers	# of *k*-mers*	# of increased unique *k*-mers	# of strains without unique *k*-mers
E. coli	1,433	192,325,016	69,097,590	60	16,071,080	30,587,430	49
S. epidermidis	995	65,345,200	28,559,277	110	7,354,669	29,709,206	26
M. tuberculosis	792	42,221,860	29,004,912	0	30,011,796	9,985,111	0
C. acnes	275	16,923,331	4,107,627	74	6,245,590	1,845,459	72
A. muciniphila	157	45,911,791	10,862,010	17	6,488,987	1,501,606	12
P. copri	112	97,438,063	42,697,948	0	7,942,630	1,509,892	0

Supplementary Table S1. Comparison of the number of *k*-mers before and after clustering. "*" in the table means these *k*-mers are from the largest cluster after clustering. "# of increased unique *k*-mers" is obtained by summing the difference between the number of unique *k*-mers before and after clustering of each strain, where strains without unique *k*-mers before clustering are not included in the calculation.

Species	# of strains	# of *k*-mers	# of chosen *k*-mers	# of strain-specific *k*-mers	# of group-specific *k*-mers	# of joint *k*-mers
E. coli	201	16,071,080	6,614,270	1,990,030	3,365,764	1,258,476
S. epidermidis	92	7,354,669	3,024,846	1,396,638	1,232,400	395,808
M. tuberculosis	768	30,011,796	8,832,212	764,110	3,778,226	4,289,876
C. acnes	70	6,245,590	2,049,674	722,356	272,526	1,054,792
A. muciniphila	46	6,488,987	832,674	556,508	206,952	69,214
P. copri	17	7,942,630	108,408	26,670	37,722	44,012

Supplementary Table S2. The statistics of different types of *k*-mers in the largest cluster of each species.

Datasets	Species/Strains	Depth	Ref
30 real sequencing datasets used in Figure 9	E. coli	20∼94X	[11, 12]
SRR172902 (HMP mock data)	C. acnes	2∼9X	[13]
	S. epidermidis	3∼10X	
	E. coli	1∼3X	
SRR172903 (HMP mock data)	C. acnes	1∼2X	
	S. epidermidis	23∼39X	
	E. coli	3∼10X	
SRR13355226 (E. coli mock community)	E. coli H10407	∼5X	[14]
	E. coli E24337A	∼3X	
	E. coli UTI89	∼3X	
	E. coli Sakai	∼1X	
12 real sequencing datasets used in Table 4	E. coli, M. tuberculosis	>10X	[15, 16]
6 cultured mixed strain metagenomic	S. epidermidis	5∼30X	[17]
1 metagenomic dataset containing low-depth Clostridioides difficile strain	Clostridioides difficile	∼1X	[18]
9 human skin metagnenomic datasets	C. acnes	>5X	[19]
18 human gut metagenomic datasets	E. coli	>10X	[5, 6]
18 human gut metagenomic datasets	P. copri	>10X	[9]

Supplementary Table S3. The approximate sequencing depths of the strains in the real sequencing datasets.

Species	Tools	TP				FN				FP				Recall				Precision			
		10X	5X	3X	1X	10X	5X	3X	1X	10X	5X	3X	1X	10X	5X	3X	1X	10X	5X	3X	1X
	Krakenuniq	16	16	15	10	44	44	45	50	77	48	45	50	0.27	0.27	0.25	0.17	0.17	0.25	0.25	0.17
	StrainSeeker	54	60	57	57	6	0	3	3	67	61	64	64	0.90	**1.00**	0.95	0.95	0.45	0.50	0.47	0.47
	StrainGE_cluster	41	41	41	41	19	19	19	19	19	19	19	19	0.68	0.68	0.68	0.68	0.68	0.68	0.68	0.68
	StrainGE_strain	14	14	14	14	46	46	46	46	46	46	46	46	0.23	0.23	0.23	0.23	0.23	0.23	0.23	0.23
A. muciniphila	Pathoscope2	54	55	55	51	6	5	5	9	434	408	362	299	0.90	0.92	0.92	0.85	0.11	0.12	0.13	0.15
	Sigma	60	60	60	58	0	0	0	2	139	204	267	341	**1.00**	**1.00**	**1.00**	**0.97**	0.30	0.23	0.18	0.15
	StrainEst_cluster	59	59	0	0	1	1	60	60	40	33	60	60	0.98	0.98	0	0	0.6	0.64	0	0
	StrainEst_strain	13	13	0	0	47	47	60	60	86	79	60	60	0.22	0.22	0	0	0.13	0.14	0	0
	StrainScan	60	59	59	51	0	1	1	9	0	1	1	5	**1.00**	0.98	0.98	0.85	**1.00**	**0.98**	**0.98**	**0.91**
	Krakenuniq	33	33	32	27	27	27	28	33	27	27	28	33	0.55	0.55	0.53	0.45	0.55	0.55	0.53	0.45
	StrainSeeker	53	56	55	58	7	4	5	2	69	64	67	62	0.88	0.93	0.92	0.97	0.43	0.47	0.45	0.48
	StrainGE_cluster	60	60	60	60	0	0	0	0	3	8	10	19	**1.00**	**1.00**	**1.00**	**1.00**	0.95	0.88	0.86	0.76
	StrainGE_strain	3	3	3	3	57	57	57	57	60	65	67	76	0.05	0.05	0.05	0.05	0.05	0.04	0.04	0.04
C. acnes	Pathoscope2	50	49	47	45	10	11	13	15	1018	910	848	720	0.83	0.82	0.78	0.75	0.05	0.05	0.05	0.06
	Sigma	57	56	54	54	3	4	6	6	1236	1214	1275	743	0.95	0.93	0.90	0.90	0.04	0.04	0.04	0.07
	StrainEst_cluster	60	60	0	0	0	0	60	60	130	119	60	60	**1.00**	**1.00**	0	0	0.32	0.34	0	0
	StrainEst_strain	9	9	0	0	51	51	60	60	181	170	60	60	0.15	0.15	0	0	0.05	0.05	0	0
	StrainScan	60	60	60	60	0	0	0	0	0	0	0	0	**1.00**	**1.00**	**1.00**	**1.00**	**1.00**	**1.00**	**1.00**	**1.00**
	Krakenuniq	13	13	12	12	37	37	38	38	685	78	47	38	0.26	0.26	0.24	0.24	0.02	0.14	0.20	0.24
	StrainSeeker	46	45	46	45	4	5	4	5	54	55	54	55	0.92	0.90	0.92	0.90	0.46	0.45	0.46	0.45
	StrainGE_cluster	50	50	50	50	0	0	0	0	0	0	0	1	**1.00**	**1.00**	**1.00**	**1.00**	**1.00**	**1.00**	**1.00**	0.98
	StrainGE_strain	13	13	13	13	37	37	37	37	37	37	37	38	0.26	0.26	0.26	0.26	0.26	0.26	0.26	0.25
P. copri	Pathoscope2	50	50	50	50	0	0	0	0	150	121	102	72	**1.00**	**1.00**	**1.00**	**1.00**	0.25	0.29	0.33	0.41
	Sigma	50	50	50	50	0	0	0	0	91	98	77	128	**1.00**	**1.00**	**1.00**	**1.00**	0.35	0.34	0.39	0.28
	StrainEst_cluster	49	49	0	0	1	1	50	50	54	42	50	50	0.98	0.98	0	0	0.48	0.54	0	0
	StrainEst_strain	13	13	0	0	37	37	50	50	90	78	50	50	0.26	0.26	0	0	0.13	0.14	0	0
	StrainScan	50	50	50	50	0	0	0	0	0	0	0	0	**1.00**	**1.00**	**1.00**	**1.00**	**1.00**	**1.00**	**1.00**	**1.00**
	Krakenuniq	27	28	27	27	33	32	33	33	571	223	39	33	0.45	0.47	0.45	0.45	0.05	0.11	0.41	0.45
	StrainSeeker	51	51	53	52	9	9	7	8	90	88	82	75	0.85	0.85	0.88	0.87	0.36	0.37	0.39	0.41
	StrainGE_cluster	59	59	59	58	1	1	1	2	4	6	9	10	0.98	0.98	0.98	**0.97**	0.94	0.91	0.87	0.85
E. coli	StrainGE_strain	18	18	18	18	42	42	42	42	45	47	50	50	0.30	0.30	0.30	0.30	0.29	0.28	0.26	0.26
	StrainEst_cluster	60	60	0	0	0	0	60	60	117	108	60	60	**1.00**	**1.00**	0	0	0.34	0.36	0	0
	StrainEst_strain	19	19	0	0	41	41	60	60	158	149	60	60	0.32	0.32	0	0	0.11	0.11	0	0
	StrainScan	60	60	60	58	0	0	0	2	0	0	0	1	**1.00**	**1.00**	**1.00**	**0.97**	**1.00**	**1.00**	**1.00**	**0.98**
	Krakenuniq	51	51	50	45	9	9	10	15	9	9	10	15	0.85	0.85	0.83	**0.75**	0.85	0.85	0.83	0.75
	StrainSeeker	0	0	0	0	60	60	60	60	0	0	0	0	0	0	0	0	0	0	0	0
	StrainGE_cluster	0	0	0	0	60	60	60	60	120	158	180	180	0	0	0	0	0	0	0	0
M. tuberculosis	StrainGE_strain	0	0	0	0	60	60	60	60	120	158	180	180	0	0	0	0	0	0	0	0
	StrainEst_cluster	53	43	0	0	7	17	60	60	617	297	0	0	0.88	0.72	0	0	0.08	0.13	0	0
	StrainEst_strain	3	3	0	0	57	57	60	60	667	337	0	0	0.05	0.05	0	0	0	0.01	0	0
	StrainScan	60	56	57	44	0	4	3	16	0	2	0	0	**1.00**	**0.93**	**0.95**	0.73	**1.00**	**0.97**	**1.00**	**1.00**
	Krakenuniq	36	38	38	33	24	22	22	27	40	22	22	27	0.60	0.63	0.63	0.55	0.47	0.63	0.63	0.55
	StrainSeeker	50	54	54	46	10	6	6	14	80	69	80	91	0.83	0.90	0.90	0.77	0.38	0.44	0.40	0.34
	StrainGE_cluster	59	59	59	59	1	1	1	1	3	4	6	7	0.98	0.98	0.98	0.98	0.95	0.94	0.91	0.89
S. epidermidis	StrainGE_strain	16	16	16	16	44	44	44	44	46	47	49	50	0.27	0.27	0.27	0.27	0.26	0.25	0.25	0.24
	StrainEst_cluster	52	30	2	0	8	30	58	60	159	66	58	60	0.87	0.50	0.03	0	0.25	0.31	0.03	0
	StrainEst_strain	10	8	1	0	50	52	59	60	201	88	59	60	0.17	0.13	0.02	0	0.05	0.08	0.02	0
	StrainScan	60	60	60	60	0	0	0	0	0	2	0	1	**1.00**	**1.00**	**1.00**	**1.00**	**1.00**	**0.97**	**1.00**	**0.98**

Supplementary Table S4. The TP, FN, FP, recall, and precision of 7 tools on “single-strain” simulated datasets under different sequencing depths. The bold text means the biggest value of the corresponding metric.

Species	Tools	TP			FN			FP			Recall			Precision		
		2 strains	3 strains	5 strains	2 strains	3 strains	5 strains	2 strains	3 strains	5 strains	2 strains	3 strains	5 strains	2 strains	3 strains	5 strains
A. muciniphila	Krakenuniq	13	19	23	7	11	27	202	214	222	0.65	0.63	0.46	0.06	0.08	0.09
	StrainSeeker	7	9	17	13	21	33	152	349	773	0.35	0.30	0.34	0.04	0.03	0.02
	StrainGE_cluster	19	27	47	1	3	3	1	3	3	0.95	0.90	0.94	0.95	0.90	0.94
	StrainGE_strain	14	19	23	6	11	27	6	11	27	0.70	0.63	0.46	0.70	0.63	0.46
	StrainEst_cluster	20	30	50	0	0	0	67	116	127	**1.00**	**1.00**	**1.00**	0.23	0.21	0.28
	StrainEst_strain	11	17	19	9	13	31	67	116	127	0.55	0.57	0.38	0.14	0.13	0.13
	StrainScan	20	30	50	0	0	0	0	0	0	**1.00**	**1.00**	**1.00**	**1.00**	**1.00**	**1.00**
C. acnes	Krakenuniq	16	20	28	4	10	22	282	510	804	0.80	0.67	0.56	0.05	0.04	0.03
	StrainSeeker	8	12	9	12	18	41	520	925	1375	0.40	0.40	0.18	0.02	0.01	0.01
	StrainGE_cluster	16	28	46	4	2	4	7	5	4	0.80	0.93	0.92	0.70	0.85	0.92
	StrainGE_strain	6	8	9	14	22	41	17	25	40	0.30	0.27	0.18	0.26	0.24	0.18
	StrainEst_cluster	19	29	50	1	1	0	56	82	120	0.95	0.97	**1.00**	0.25	0.26	0.29
	StrainEst_strain	6	6	12	14	24	38	56	82	120	0.30	0.20	0.24	0.10	0.07	0.09
	StrainScan	20	30	50	0	0	0	0	0	0	**1.00**	**1.00**	**1.00**	**1.00**	**1.00**	**1.00**
P. copri	Krakenuniq	2	6	13	18	24	37	262	276	313	0.10	0.20	0.26	0.01	0.02	0.04
	StrainSeeker	7	13	12	13	17	38	62	104	297	0.35	0.43	0.24	0.10	0.11	0.04
	StrainGE_cluster	20	30	50	0	0	0	0	0	0	**1.00**	**1.00**	**1.00**	**1.00**	**1.00**	**1.00**
	StrainGE_strain	4	9	15	16	21	35	16	21	33	0.20	0.30	0.30	0.20	0.30	0.31
	StrainEst_cluster	20	30	50	0	0	0	32	74	87	**1.00**	**1.00**	**1.00**	0.38	0.29	0.36
	StrainEst_strain	2	7	14	18	23	36	32	74	87	0.10	0.23	0.28	0.06	0.09	0.14
	StrainScan	20	30	50	0	0	0	0	0	0	**1.00**	**1.00**	**1.00**	**1.00**	**1.00**	**1.00**
E. coli	Krakenuniq	13	21	32	7	9	18	2565	3297	4234	0.65	0.70	0.64	0.01	0.01	0.01
	StrainSeeker	6	10	16	14	20	34	2754	4234	4957	0.30	0.33	0.32	0	0	0
	StrainGE_cluster	20	28	48	0	2	2	0	2	2	**1.00**	0.93	0.96	**1.00**	0.93	0.96
	StrainGE_strain	16	22	39	4	8	11	4	8	11	0.80	0.73	0.78	0.80	0.73	0.78
	StrainEst_cluster	19	28	44	1	2	6	106	153	241	0.95	0.93	0.88	0.15	0.15	0.15
	StrainEst_strain	7	9	17	13	21	33	106	153	241	0.35	0.30	0.34	0.06	0.06	0.07
	StrainScan	20	30	50	0	0	0	1	0	0	**1.00**	**1.00**	**1.00**	0.95	**1.00**	**1.00**
M. tuberculosis	Krakenuniq	18	29	38	2	1	12	1671	2782	4229	0.90	0.97	0.76	0.01	0.01	0.01
	StrainSeeker	0	0	0	20	30	50	0	0	0	0	0	0	0	0	0
	StrainGE_cluster	3	9	4	17	21	26	17	21	26	0.15	0.30	0.13	0.15	0.30	0.13
	StrainGE_strain	3	9	4	17	21	26	17	14	9	0.15	0.30	0.13	0.15	0.39	0.31
	StrainEst_cluster	16	21	39	4	9	11	134	194	244	0.80	0.70	0.78	0.11	0.10	0.14
	StrainEst_strain	1	0	5	19	30	45	134	194	244	0.05	0	0.10	0.01	0	0.02
	StrainScan	20	30	50	0	0	0	0	0	0	**1.00**	**1.00**	**1.00**	**1.00**	**1.00**	**1.00**
S. epidermidis	Krakenuniq	9	16	32	11	14	18	450	779	1336	0.45	0.53	0.64	0.02	0.02	0.02
	StrainSeeker	10	10	17	10	20	33	2004	3040	3415	0.50	0.33	0.34	0	0	0
	StrainGE_cluster	19	28	44	1	2	6	1	2	6	0.95	0.93	0.88	0.95	0.93	0.88
	StrainGE_strain	6	9	16	14	21	34	14	21	29	0.30	0.30	0.32	0.30	0.30	0.36
	StrainEst_cluster	16	22	43	4	8	7	45	63	94	0.80	0.73	0.86	0.26	0.26	0.31
	StrainEst_strain	1	1	2	19	29	48	45	63	94	0.05	0.03	0.04	0.02	0.02	0.02
	StrainScan	20	30	50	0	0	0	0	0	0	**1.00**	**1.00**	**1.00**	**1.00**	**1.00**	**1.00**

Supplementary Table S5. The TP, FN, FP, recall, and precision of 5 tools on "multiple-strain" simulated datasets, which contain strains from different clusters. The bold text means the biggest value of the corresponding metric.

Species	Tools	TP			FN			FP			Recall			Precision		
		2 strains	3 strains	5 strains	2 strains	3 strains	5 strains	2 strains	3 strains	5 strains	2 strains	3 strains	5 strains	2 strains	3 strains	5 strains
A. muciniphila	Krakenuniq	5	11	17	15	19	33	222	211	250	0.25	0.37	0.34	0.02	0.05	0.06
	StrainSeeker	17	20	17	3	10	33	65	355	357	0.85	0.67	0.34	0.21	0.05	0.05
	StrainGE_cluster	18	21	21	2	9	29	2	9	29	0.90	0.70	0.42	0.90	0.70	0.42
	StrainGE_strain	4	4	3	16	26	47	6	6	7	0.20	0.13	0.06	0.40	0.40	0.30
	StrainEst_cluster	20	30	49	0	0	1	20	17	12	**1.00**	**1.00**	**0.98**	0.50	0.64	0.80
	StrainEst_strain	5	4	3	15	26	47	20	17	12	0.25	0.13	0.06	0.20	0.19	0.20
	StrainScan	20	30	47	0	0	3	0	0	3	**1.00**	**1.00**	0.94	**1.00**	**1.00**	**0.94**
C. acnes	Krakenuniq	9	15	27	11	15	23	323	509	788	0.45	0.50	0.54	0.03	0.03	0.03
	StrainSeeker	9	13	20	11	17	30	540	853	1096	0.45	0.43	0.40	0.02	0.02	0.02
	StrainGE_cluster	20	30	30	0	0	20	0	0	20	**1.00**	**1.00**	0.60	**1.00**	**1.00**	0.60
	StrainGE_strain	1	1	1	19	29	49	9	11	14	0.05	0.03	0.02	0.10	0.08	0.07
	StrainEst_cluster	20	30	49	0	0	1	30	28	33	**1.00**	**1.00**	**0.98**	0.40	0.52	0.60
	StrainEst_strain	2	3	3	18	27	47	30	28	33	0.10	0.10	0.06	0.06	0.10	0.08
	StrainScan	20	29	48	0	1	2	0	1	3	**1.00**	0.97	0.96	**1.00**	0.97	**0.94**
P. copri	Krakenuniq	0	1	2	20	29	48	267	282	322	0	0.03	0.04	0	0	0.01
	StrainSeeker	13	15	20	7	15	30	17	23	147	0.65	0.50	0.40	0.43	0.39	0.12
	StrainGE_cluster	20	30	50	0	0	0	0	0	0	**1.00**	**1.00**	**1.00**	**1.00**	**1.00**	**1.00**
	StrainGE_strain	2	2	5	18	28	45	8	8	5	0.10	0.07	0.10	0.20	0.20	0.50
	StrainEst_cluster	20	30	50	0	0	0	19	31	26	**1.00**	**1.00**	**1.00**	0.51	0.49	0.66
	StrainEst_strain	0	1	4	20	29	46	19	31	26	0	0.03	0.08	0	0.03	0.13
	StrainScan	20	30	49	0	0	1	0	0	2	**1.00**	**1.00**	0.98	**1.00**	**1.00**	0.96
E. coli	Krakenuniq	3	10	9	17	20	41	2666	3440	4318	0.15	0.33	0.18	0	0	0
	StrainSeeker	17	19	25	3	11	25	2413	4035	4010	0.85	0.63	0.50	0.01	0	0.01
	StrainGE_cluster	20	30	50	0	0	0	0	0	0	**1.00**	**1.00**	**1.00**	**1.00**	**1.00**	**1.00**
	StrainGE_strain	9	5	3	11	25	47	1	5	7	0.45	0.17	0.06	0.90	0.50	0.30
	StrainEst_cluster	20	27	45	0	3	5	20	45	49	**1.00**	0.90	0.90	0.50	0.38	0.48
	StrainEst_strain	7	5	4	13	25	46	20	45	49	0.35	0.17	0.08	0.26	0.10	0.08
	StrainScan	20	30	49	0	0	1	0	0	3	**1.00**	**1.00**	0.98	**1.00**	**1.00**	0.94
M. tuberculosis	Krakenuniq	15	22	34	5	8	16	1660	3045	4345	0.75	0.73	0.68	0.01	0.01	0.01
	StrainSeeker	0	0	0	20	30	50	0	0	0	0	0	0	0	0	0
	StrainGE_cluster	0	0	0	20	30	50	20	30	50	0	0	0	0	0	0
	StrainGE_strain	0	0	0	20	30	50	20	20	20	0	0	0	0	0	0
	StrainEst_cluster	14	25	36	6	5	14	151	155	226	0.70	0.83	0.72	0.08	0.14	0.14
	StrainEst_strain	1	3	5	19	27	45	151	155	226	0.05	0.10	0.10	0.01	0.02	0.02
	StrainScan	20	30	50	0	0	0	1	0	0	**1.00**	**1.00**	**1.00**	**0.95**	**1.00**	**1.00**
S. epidermidis	Krakenuniq	3	10	20	17	20	30	472	728	1310	0.15	0.33	0.4	0.01	0.01	0.02
	StrainSeeker	15	18	22	5	12	28	2910	2575	3893	0.75	0.60	0.44	0.01	0.01	0.01
	StrainGE_cluster	20	30	45	0	0	5	0	0	5	**1.00**	**1.00**	0.90	**1.00**	**1.00**	0.90
	StrainGE_strain	5	3	1	15	27	49	5	7	9	0.25	0.10	0.02	0.50	0.30	0.10
	StrainEst_cluster	18	30	50	2	0	0	48	42	35	0.90	**1.00**	**1.00**	0.27	0.42	0.59
	StrainEst_strain	0	2	1	20	28	49	48	42	35	0	0.07	0.02	0	0.05	0.03
	StrainScan	20	30	50	0	0	0	0	0	1	**1.00**	**1.00**	**1.00**	**1.00**	**1.00**	**0.98**

Supplementary Table S6. The TP, FN, FP, recall, and precision of 5 tools on "multiple-strain" simulated datasets, which contain strains from the same cluster. The bold text means the biggest value of the corresponding metric.

Species	Tools	1X, 10X			3X, 10X			5X, 10X			10X, 10X		
		Recall	Precision	F1 score	Recall	Precision	F1 score	Recall	Precision	F1 score	Recall	Precision	F1 score
	Krakenuniq	0.65	0.43	0.52	0.65	0.28	0.39	0.65	0.21	0.31	0.65	0.13	0.21
	StrainSeeker	0.40	0.10	0.16	0.45	0.15	0.22	0.40	0.14	0.20	0.45	0.16	0.23
	StrainGE_cluster	0.95	0.95	0.95	0.95	0.95	0.95	0.95	0.95	0.95	0.95	0.95	0.95
A. mucinipa	StrainGE_strain	0.70	0.70	0.70	0.70	0.70	0.70	0.70	0.70	0.70	0.70	0.70	0.70
	StrainEst_cluster	0.90	0.24	0.38	1.00	0.22	0.36	1.00	0.23	0.38	1.00	0.22	0.36
	StrainEst_strain	0.45	0.13	0.20	0.55	0.14	0.22	0.55	0.14	0.22	0.55	0.13	0.21
	StrainScan	1.00	1.00	**1.00**	1.00	1.00	**1.00**	1.00	1.00	**1.00**	1.00	1.00	**1.00**
	Krakenuniq	0.80	1.00	0.88	0.80	1.00	0.88	0.80	1.00	0.88	0.80	0.84	0.82
	StrainSeeker	0.45	0.03	0.06	0.45	0.03	0.06	0.35	0.02	0.05	0.20	0.11	0.14
	StrainGE_cluster	0.85	0.77	0.81	0.90	0.81	0.86	0.95	0.86	0.90	0.95	0.90	0.93
C. acnes	StrainGE_strain	0.35	0.32	0.33	0.35	0.32	0.33	0.35	0.32	0.33	0.35	0.33	0.34
	StrainEst_cluster	0.85	0.25	0.38	1.00	0.26	0.42	1.00	0.28	0.43	1.00	0.25	0.40
	StrainEst_strain	0.25	0.09	0.13	0.30	0.10	0.15	0.30	0.10	0.15	0.30	0.09	0.14
	StrainScan	1.00	0.95	**0.98**	1.00	1.00	**1.00**	1.00	1.00	**1.00**	1.00	1.00	**1.00**
	Krakenuniq	0.10	0.01	0.02	0.10	0.01	0.02	0.10	0.01	0.02	0.10	0.01	0.02
	StrainSeeker	0.45	0.13	0.20	0.50	0.15	0.24	0.45	0.13	0.21	0.50	0.16	0.25
	StrainGE_cluster	1.00	1.00	1.00	1.00	1.00	1.00	1.00	1.00	1.00	1.00	1.00	1.00
P. copri	StrainGE_strain	0.20	0.20	0.20	0.20	0.20	0.20	0.20	0.20	0.20	0.20	0.20	0.20
	StrainEst_cluster	0.95	0.37	0.52	1.00	0.31	0.48	1.00	0.29	0.44	1.00	0.26	0.42
	StrainEst_strain	0.10	0.06	0.07	0.10	0.04	0.05	0.10	0.04	0.05	0.10	0.04	0.05
	StrainScan	1.00	1.00	**1.00**	1.00	1.00	**1.00**	1.00	1.00	**1.00**	1.00	1.00	**1.00**
	Krakenuniq	0.70	0.12	0.21	0.80	0.09	0.16	0.80	0.08	0.14	0.85	0.05	0.09
	StrainSeeker	0.30	0.08	0.12	0.30	0.08	0.12	0.30	0.08	0.12	0.40	0.07	0.12
	StrainGE_cluster	1.00	1.00	1.00	1.00	1.00	1.00	1.00	1.00	1.00	1.00	1.00	1.00
E. coli	StrainGE_strain	0.80	0.80	0.80	0.80	0.80	0.80	0.80	0.80	0.80	0.80	0.80	0.80
	StrainEst_cluster	0.38	0.13	0.19	1.00	0.14	0.25	1.00	0.16	0.27	1.00	0.17	0.29
	StrainEst_strain	0.15	0.06	0.08	0.35	0.06	0.10	0.35	0.06	0.10	0.35	0.07	0.11
	StrainScan	1.00	1.00	**1.00**	1.00	1.00	**1.00**	1.00	1.00	**1.00**	1.00	1.00	**1.00**
	Krakenuniq	0.90	1.00	**0.94**	0.95	1.00	**0.97**	0.95	0.95	**0.95**	0.95	0.61	0.75
	StrainSeeker	0	0	0	0	0	0	0	0	0	0	0	0
	StrainGE_cluster	0.15	0.15	0.15	0.15	0.15	0.15	0.15	0.15	0.15	0.15	0.15	0.15
M. tuberculosis	StrainGE_strain	0.15	0.15	0.15	0.15	0.15	0.15	0.15	0.15	0.15	0.15	0.15	0.15
	StrainEst_cluster	0.38	0.09	0.15	0.80	0.11	0.19	0.85	0.11	0.20	0.80	0.11	0.19
	StrainEst_strain	0	0	0	0.05	0.01	0.01	0.05	0.01	0.01	0	0	0
	StrainScan	0.80	1.00	0.89	0.85	1.00	0.92	0.90	1.00	**0.95**	0.90	1.00	**0.95**
	Krakenuniq	0.45	0.56	0.50	0.50	0.41	0.45	0.50	0.30	0.38	0.55	0.23	0.33
	StrainSeeker	0.45	0.09	0.15	0.45	0.12	0.19	0.50	0.08	0.13	0.45	0.03	0.06
	StrainGE_cluster	0.95	0.95	0.95	0.95	0.95	0.95	0.95	0.95	0.95	0.95	0.95	0.95
S. epidermidis	StrainGE_strain	0.30	0.30	0.30	0.30	0.30	0.30	0.30	0.30	0.30	0.30	0.30	0.30
	StrainEst_cluster	0.70	0.30	0.42	0.80	0.23	0.36	0.80	0.24	0.37	0.80	0.20	0.32
	StrainEst_strain	0.05	0.02	0.03	0.05	0.02	0.03	0.05	0.02	0.03	0.05	0.02	0.03
	StrainScan	0.95	1.00	**0.97**	1.00	1.00	**1.00**	1.00	1.00	**1.00**	1.00	1.00	**1.00**

Supplementary Table S7. The recall, precision, and F1 score of 5 tools on the low-depth simulated datasets. Each dataset contains one dominant and one minor strain from different clusters. The bold text: the biggest value of F1 score at the strain level.

Species	Tools	1X, 10X			3X, 10X			5X, 10X			10X, 10X		
		Recall	Precision	F1 score	Recall	Precision	F1 score	Recall	Precision	F1 score	Recall	Precision	F1 score
A. mucinipa	Krakenuniq	0.30	0.21	0.24	0.35	0.16	0.22	0.35	0.10	0.15	0.35	0.06	0.11
	StrainSeeker	0.85	0.63	**0.72**	0.85	0.63	0.72	0.85	0.63	0.72	0.85	0.63	0.72
	StrainGE_cluster	0.90	0.90	0.90	0.90	0.90	0.90	0.90	0.90	0.90	0.90	0.90	0.90
	StrainGE_strain	0.20	0.40	0.27	0.20	0.40	0.27	0.20	0.40	0.27	0.20	0.40	0.27
	StrainEst_cluster	1.00	0.51	0.68	1.00	0.53	0.69	1.00	0.53	0.69	1.00	0.50	0.67
	StrainEst_strain	0.25	0.21	0.23	0.25	0.22	0.23	0.25	0.22	0.23	0.25	0.22	0.23
	StrainScan	0.50	1.00	0.67	0.65	1.00	**0.79**	0.60	1.00	**0.75**	0.85	1.00	**0.92**
C. acnes	Krakenuniq	0.45	1.00	0.62	0.45	1.00	0.62	0.45	1.00	0.62	0.45	1.00	0.62
	StrainSeeker	0.40	0.25	0.30	0.35	0.22	0.26	0.40	0.24	0.30	0.45	0.29	0.35
	StrainGE_cluster	1.00	1.00	1.00	1.00	1.00	1.00	1.00	1.00	1.00	1.00	1.00	1.00
	StrainGE_strain	0.05	0.10	0.07	0.05	0.10	0.07	0.05	0.10	0.07	0.05	0.10	0.07
	StrainEst_cluster	1.00	0.41	0.58	1.00	0.40	0.57	1.00	0.41	0.58	1.00	0.41	0.58
	StrainEst_strain	0.10	0.06	0.08	0.10	0.06	0.08	0.10	0.06	0.08	0.10	0.06	0.08
	StrainScan	0.50	1.00	**0.67**	1.00	0.80	**0.89**	0.95	1.00	**0.97**	1.00	0.96	**0.98**
P. copri	Krakenuniq	0	0	0	0	0	0	0	0	0	0	0	0
	StrainSeeker	0.70	0.54	0.61	0.70	0.52	0.60	0.70	0.52	0.60	0.70	0.54	0.61
	StrainGE_cluster	1.00	1.00	1.00	1.00	1.00	1.00	1.00	1.00	1.00	1.00	1.00	1.00
	StrainGE_strain	0.10	0.20	0.13	0.10	0.20	0.13	0.10	0.20	0.13	0.10	0.20	0.13
	StrainEst_cluster	1.00	0.50	0.67	1.00	0.50	0.67	1.00	0.50	0.67	1.00	0.50	0.67
	StrainEst_strain	0.10	0.06	0.08	0.10	0.06	0.08	0.10	0.06	0.08	0.10	0.06	0.08
	StrainScan	0.50	1.00	**0.67**	0.85	1.00	**0.92**	1.00	1.00	**1.00**	1.00	1.00	**1.00**
E. coli	Krakenuniq	0.15	0.02	0.05	0.15	0.01	0.03	0.15	0.01	0.03	0.15	0.01	0.03
	StrainSeeker	0.80	0.26	0.40	0.85	0.21	0.35	0.85	0.29	0.44	0.85	0.15	0.26
	StrainGE_cluster	1.00	1.00	1.00	1.00	1.00	1.00	1.00	1.00	1.00	1.00	1.00	1.00
	StrainGE_strain	0.45	0.90	0.60	0.45	0.90	0.60	0.45	0.90	0.60	0.45	0.90	0.60
	StrainEst_cluster	1.00	0.53	0.69	1.00	0.53	0.69	1.00	0.49	0.66	1.00	0.49	0.66
	StrainEst_strain	0.35	0.28	0.31	0.35	0.28	0.31	0.35	0.25	0.29	0.35	0.25	0.29
	StrainScan	0.50	1.00	**0.67**	0.55	1.00	**0.71**	0.65	1.00	**0.79**	1.00	1.00	**1.00**
M. tuberculosis	Krakenuniq	0.85	1.00	**0.92**	0.95	0.95	**0.95**	0.95	0.95	**0.95**	0.95	0.48	0.63
	StrainSeeker	0	0	0	0	0	0	0	0	0	0	0	0
	StrainGE_cluster	0	0	0	0	0	0	0	0	0	0	0	0
	StrainGE_strain	0	0	0	0	0	0	0	0	0	0	0	0
	StrainEst_cluster	0.60	0.07	0.13	0.80	0.09	0.17	0.90	0.10	0.19	0.18	0.10	0.13
	StrainEst_strain	0	0	0	0.05	0.01	0.02	0.05	0.01	0.02	0	0	0
	StrainScan	0.50	1.00	0.67	0.95	0.90	0.93	1.00	0.91	**0.95**	1.00	0.91	**0.95**
S. epidermidis	Krakenuniq	0.15	0.30	0.20	0.20	0.17	0.19	0.20	0.13	0.16	0.20	0.10	0.14
	StrainSeeker	0.75	0.43	0.54	0.75	0.38	0.50	0.75	0.11	0.19	0.80	0.12	0.20
	StrainGE_cluster	1.00	1.00	1.00	1.00	1.00	1.00	1.00	1.00	1.00	1.00	1.00	1.00
	StrainGE_strain	0.45	0.90	0.60	0.45	0.90	0.60	0.45	0.90	0.60	0.45	0.90	0.60
	StrainEst_cluster	0.90	0.30	0.44	0.80	0.29	0.42	0.84	0.33	0.47	0.80	0.24	0.37
	StrainEst_strain	0	0	0	0	0	0	0	0	0	0	0	0
	StrainScan	0.50	1.00	**0.67**	0.55	1.00	**0.71**	0.79	1.00	**0.82**	0.85	1.00	**0.92**

Supplementary Table S8. The recall, precision, and F1 score of 5 tools on the low-depth simulated datasets. Each dataset contains one dominant and one minor strain from the same cluster. The bold text: the biggest value of F1 score at the strain level.

RefSeq accesion	CST cluster size	1-Mash distance	Release date	Cluster missing	Closest cluster	Identified Cluster
GCF_021441965	1	[0.98, 0.99)	2022-01-12	Y	C376	C376
GCF_021559855	1	[0.98, 0.99)	2022-01-24	Y	C515	C515
GCF_022493195	1	[0.98, 0.99)	2022-03-07	Y	C326	C326
GCF_023206615	1	[0.98, 0.99)	2022-05-02	Y	C691	C691
GCF_021484785	3	[0.98, 0.99)	2022-01-16	Y	C820	C820
GCF_022493415	3	[0.98, 0.99)	2022-03-07	Y	C820	C820
GCF_021534865	1	[0.99, 0.995)	2022-01-20	Y	C786	C786
GCF_021535025	1	[0.99, 0.995)	2022-01-20	Y	C467	C467
GCF_022385195	1	[0.99, 0.995)	2022-02-24	Y	C17	C17
GCF_022385215	1	[0.99, 0.995)	2022-02-24	Y	C17	C17
GCF_022493115	1	[0.99, 0.995)	2022-03-07	Y	C351	C351
GCF_022493295	1	[0.99, 0.995)	2022-03-07	Y	C671	C671
GCF_022493335	1	[0.99, 0.995)	2022-03-07	Y	C704	C704
GCF_022493455	1	[0.99, 0.995)	2022-03-07	Y	C79	C79
GCF_022493735	1	[0.99, 0.995)	2022-03-07	Y	C351	C351
GCF_022493875	1	[0.99, 0.995)	2022-03-07	Y	C63	C63
GCF_022494785	1	[0.99, 0.995)	2022-03-07	Y	C93	C93
GCF_022846235	1	[0.99, 0.995)	2022-03-25	Y	C332	C332
GCF_021498045	2	[0.99, 0.995)	2022-01-18	Y	C674	C674
GCF_022493255	4	[0.99, 0.995)	2022-03-07	Y	C274	C274
GCF_022493475	4	[0.99, 0.995)	2022-03-07	Y	C274	C274
GCF_021378315	1	[0.995, 0.999)	2022-01-06	Y	C225	C225
GCF_021398465	1	[0.995, 0.999)	2022-01-10	Y	C425	C425
GCF_021398995	1	[0.995, 0.999)	2022-01-10	Y	C458	C458
GCF_021498025	1	[0.995, 0.999)	2022-01-18	Y	C242	C242
GCF_021498165	1	[0.995, 0.999)	2022-01-18	Y	C97	C97
GCF_021534985	1	[0.995, 0.999)	2022-01-20	Y	C264	C264
GCF_022220885	1	[0.995, 0.999)	2022-02-14	Y	C635	C635
GCF_022368815	1	[0.995, 0.999)	2022-02-23	Y	C107	C107
GCF_022370515	1	[0.995, 0.999)	2022-02-23	Y	C159	C159
GCF_022492995	1	[0.995, 0.999)	2022-03-07	Y	C184	C184
GCF_022493355	1	[0.995, 0.999)	2022-03-07	Y	C115	C115
GCF_022493495	1	[0.995, 0.999)	2022-03-07	Y	C255	C255
GCF_022493655	1	[0.995, 0.999)	2022-03-07	Y	C111	C111
GCF_022493755	1	[0.995, 0.999)	2022-03-07	Y	C115	C115
GCF_022493855	1	[0.995, 0.999)	2022-03-07	Y	C150	C150
GCF_022494235	1	[0.995, 0.999)	2022-03-07	Y	C521	C521
GCF_022494685	1	[0.995, 0.999)	2022-03-07	Y	C424	C424
GCF_022494705	1	[0.995, 0.999)	2022-03-07	Y	C695	C695
GCF_022494725	1	[0.995, 0.999)	2022-03-07	Y	C109	C109
GCF_022494825	1	[0.995, 0.999)	2022-03-07	Y	C470	C470
GCF_022558685	1	[0.995, 0.999)	2022-03-14	Y	C362	C362
GCF_022559285	1	[0.995, 0.999)	2022-03-14	Y	C136	C136
GCF_022559305	1	[0.995, 0.999)	2022-03-14	Y	C140	C140
GCF_022559325	1	[0.995, 0.999)	2022-03-14	Y	C137	C137
GCF_022559875	1	[0.995, 0.999)	2022-03-14	Y	C505	C505
GCF_022919035	1	[0.995, 0.999)	2022-04-12	Y	C500	C500
GCF_022964815	1	[0.995, 0.999)	2022-04-13	Y	C593	C593
GCF_022494745	2	[0.995, 0.999)	2022-03-07	N	-	-

RefSeq accesion	CST cluster size	1-Mash distance	Release date	Cluster missing	Closest cluster	Identified Cluster
GCF_022559245	2	[0.995, 0.999)	2022-03-14	Y	C484	C484
GCF_022493375	3	[0.995, 0.999)	2022-03-07	Y	C13	C13
GCF_022493435	4	[0.995, 0.999)	2022-03-07	Y	C398	C398
GCF_022558665	4	[0.995, 0.999)	2022-03-14	N	-	-
GCF_022558705	4	[0.995, 0.999)	2022-03-14	N	-	-
GCF_022558765	4	[0.995, 0.999)	2022-03-14	N	-	-
GCF_022558785	4	[0.995, 0.999)	2022-03-14	N	-	-
GCF_022559025	4	[0.995, 0.999)	2022-03-14	Y	C290	C290
GCF_022559065	10	[0.995, 0.999)	2022-03-14	Y	C358	C358
GCF_021496425	33	[0.995, 0.999)	2022-01-18	N	-	-
GCF_022220385	120	[0.995, 0.999)	2022-02-14	Y	C192	C192
GCF_021398485	1	[0.999, 1)	2022-01-10	N	-	-
GCF_021398935	1	[0.999, 1)	2022-01-10	N	-	-
GCF_021559815	1	[0.999, 1)	2022-01-24	N	-	-
GCF_022453605	1	[0.999, 1)	2022-03-03	N	-	-
GCF_022494805	1	[0.999, 1)	2022-03-07	N	-	-
GCF_022558725	1	[0.999, 1)	2022-03-14	N	-	-
GCF_022559685	1	[0.999, 1)	2022-03-14	N	-	-
GCF_022918835	1	[0.999, 1)	2022-04-12	N	-	-
GCF_022559005	3	[0.999, 1)	2022-03-14	N	-	-
GCF_022759825	3	[0.999, 1)	2022-03-30	N	-	-
GCF_022759845	3	[0.999, 1)	2022-03-30	N	-	-
GCF_022759865	3	[0.999, 1)	2022-03-30	N	-	-
GCF_022759885	3	[0.999, 1)	2022-03-30	N	-	-
GCF_022759905	3	[0.999, 1)	2022-03-30	N	-	-
GCF_023206635	3	[0.999, 1)	2022-05-02	N	-	-
GCF_023206655	3	[0.999, 1)	2022-05-02	N	-	-
GCF_023206675	3	[0.999, 1)	2022-05-02	N	-	-
GCF_023206695	3	[0.999, 1)	2022-05-02	N	-	-
GCF_023206715	3	[0.999, 1)	2022-05-02	N	-	-
GCF_023206735	3	[0.999, 1)	2022-05-02	N	-	-
GCF_022759725	4	[0.999, 1)	2022-03-30	N	-	-
GCF_022759745	4	[0.999, 1)	2022-03-30	N	-	-
GCF_022759765	4	[0.999, 1)	2022-03-30	N	-	-
GCF_022759785	4	[0.999, 1)	2022-03-30	N	-	-
GCF_022759805	4	[0.999, 1)	2022-03-30	N	-	-
GCF_021496445	33	[0.999, 1)	2022-01-18	N	-	-
GCF_021559875	33	[0.999, 1)	2022-01-24	N	-	-
GCF_022221385	120	[0.999, 1)	2022-02-14	N	-	-
GCF_022591215	120	[0.999, 1)	2022-03-16	N	-	-
GCF_022869945	201	[0.999, 1)	2022-04-11	N	-	-

Supplementary Table S9. The 90 *E. coli* genomes downloaded from NCBI. CST cluster size: the cluster size of the closest match of the actual strain in the StrainScan's reference database. Clustering missing: whether the cluster of the strain is missing from the pre-build database. Closest cluster: the closest matched cluster in the database of the actual strain.

Dataset ID	Strains ID (Depth)	Truth	StrainScan	StrainGE	StrainEst	StrainSeeker	Krakenuniq
D1	GCF_022493495 (100X)<br>GCF_022492995 (10X)	(0.002298, 0.004087, 2)	**(0.002298, 0.004087, 2)**	**(0.002298, 0.004087, 2)**	**(0.002298, 0.004087, 12)**	**(0.002298, 0.004087, 16)**	**(0.002298, 0.004087, 1199)**
D2	GCF_022759865 (100X)<br>GCF_022494725 (10X)	(0.000288, 0.003489, 2)	**(0.000288, 0.003489, 2)**	(0.000680, 0.003489, 2)	(0.003726, 0.005055, 14)	(0.000288, 0.015508, 263)	(0.015454, 0.037938, 1220)
D3	GCF_022759885 (100X)<br>GCF_022493655 (10X)	(0.000288, 0.001600, 2)	**(0.000288, 0.001600, 2)**	(0.000680, 0.001600, 2)	(0.003726, 0.001600, 10)	(0.000288, 0.010229, 99)	(0.037811, 0.036818, 1210)
D4	GCF_022559305 (100X)<br>GCF_022558685 (10X)	(0.002380, 0.003169, 2)	**(0.002380, 0.003169, 2)**	**(0.002380, 0.003169, 2)**	**(0.002380, 0.003169, 7)**	(0.005808, 0.003169, 176)	(0.002380, 0.037311, 1244)
D5	GCF_022559285 (100X)<br>GCF_021559815 (10X)	(0.002134, 2.382e-05, 2)	**(0.002134, 2.382e-05, 2)**	(0.002134, 0.003519, 2)	(0.002134, 0.001999, 18)	(0.034136, 0.004393, 77)	(0.002134, 0.041426, 1233)
D6	GCF_021398485 (100X)<br>GCF_021378315 (10X)	(0.000606, 0.001865, 2)	**(0.000606, 0.001865, 2)**	(0.002742, 0.001865, 3)	(0.002107, 0.001865, 8)	(0.014871, 0.004148, 44)	(0.014871, 0.015833, 1200)
D7	GCF_022559685 (100X)<br>GCF_022494745 (10X)	(0.000705, 0.001262, 2)	**(0.000705, 0.001262, 2)**	(0.002080, 0.001391, 3)	(0.003489, 0.001732, 9)	(0.003343, 0.031074, 269)	(0.000705, 0.039233, 1243)
D8	GCF_021559855 (100X)<br>GCF_022964815 (10X)	(0.012214, 0.001522, 2)	**(0.012214, 0.001522, 2)**	(0.002408, 0.002271, 2)	(0.002026, 0.002271, 6)	(0.000119, 0.015998, 27)	(0.041143, 0.042290, 1216)
D9	GCF_021559875 (100X)<br>GCF_023206715 (10X)	(0.000264, 2.382e-05, 2)	(0.000264, 7.158e-05, 2)	(0.001574, 0.001732, 2)	(0.000483, 0.001999, 8)	(0.000409, 0.014150, 34)	(0.041568, 0.042144, 1221)
D10	GCF_021496425 (100X)<br>GCF_023206735 (10X)	(0.001134, 2.382e-05, 2)	(0.001679, 2.382e-05, 2)	(0.001574, 0.001732, 2)	(0.001865, 0.001999, 10)	(0.013848, 7.158e-05, 46)	(0.041284, 0.042144, 1233)
D11	GCF_022453605 (100X)<br>GCF_021498025 (10X)	(0.000336, 0.001443, 2)	**(0.000336, 0.001443, 2)**	**(0.000336, 0.001443, 2)**	**(0.000336, 0.001443, 8)**	(0.014200, 0.001443, 141)	(0.000336, 0.036214, 1218)
D12	GCF_022493375 (100X)<br>GCF_021398465 (10X)	(0.002855, 0.001785, 2)	**(0.002855, 0.001785, 2)**	(0.002940, 0.001785, 2)	(0.003140, 0.002883, 12)	(0.029423, 0.001785, 339)	(0.014923, 0.038969, 1219)
D13	GCF_021398995 (100X)<br>GCF_022558765 (10X)	(0.002353, 0.001032, 2)	**(0.002353, 0.001032, 2)**	(0.002997, 0.002161, 3)	(0.005542, 0.031174, 8)	(0.002997, 0.032301, 135)	(0.002353, 0.037311, 1223)
D14	GCF_022558725 (100X)<br>GCF_021534865 (10X)	(0.000906, 0.005281, 2)	**(0.000906, 0.005281, 2)**	(0.002271, 0.005281, 2)	(0.003696, 0.005281, 15)	(0.003548, 0.016330, 668)	(0.014507, 0.016330, 1236)
D15	GCF_022385195 (100X)<br>GCF_022385215 (10X)	(0.005708, 0.005346, 1)	**(0.005708, 0.005346, 1)**	**(0.005708, 0.005346, 2)**	(0.005708, 0.006698, 10)	(0.013452, 0.013015, 131)	(0.027600, 0.005346, 1233)
D16	GCF_022759865 (100X)<br>GCF_022759825 (10X)	(0.000288, 0.000287, 1)	**(0.000288, 0.000287, 1)**	(0.012075, 0.012065, 1)	(0.003726, 0.011308, 6)	(0.000288, 0.000981, 121)	(0.037811, 0.038320, 1228)
D17	GCF_022759885 (100X)<br>GCF_022759845 (10X)	(0.000288, 0.000287, 1)	**(0.000288, 0.000287, 1)**	(0.012075, 0.012065, 1)	(0.003726, 0.011308, 6)	(0.000981, 0.000288, 107)	(0.037811, 0.038320, 1218)
D18	GCF_021559815 (100X)<br>GCF_021398485 (10X)	(2.382e-05, 0.000606, 1)	**(2.382e-05, 0.000606, 1)**	(0.002912, 0.002742, 2)	(0.001999, 0.015240, 7)	(0.004178, 0.004178, 38)	(2.382e-05, 0.041003, 1214)
D19	GCF_022558725 (100X)<br>GCF_022559685 (10X)	(0.000906, 0.000705, 1)	**(0.000906, 0.000705, 1)**	(0.002271, 0.002080, 1)	(0.003696, 0.003112, 6)	(0.000906, 0.003343, 314)	(0.000906, 0.012121, 1237)
D20	GCF_023206715 (100X)<br>GCF_023206735 (10X)	(2.382e-05, 2.348e-05, 1)	**(2.382e-05, 2.348e-05, 1)**	(0.001732, 0.001742, 1)	(0.001999, 0.012729, 5)	(2.382e-05, 7.158e-05, 46)	(0.040724, 0.042144, 1204)

Supplementary Table S10. The mash distance and the number of strains identified by five tools in 20 simulated datasets. Each cell contains a tuple, with the first and second numbers being the mash distance between the closest matched or identified strains and the true strains and the third number being the number of the closest matched or identified strains. "Truth": the closest matched strains in the database of the actual strains. The bold text: the mash distance same to the ground truth.

Species	Group	TP	TN	FP	Recall	Precision	F1 score
P. copri	single-strain simulated data	50	0	0	1	1	1
	single-strain simulated data + SRR769529	50	0	0	1	1	1
	multiple-strain simulated data	50	0	0	1	1	1
	multiple-strain simulated data + SRR769529	50	0	0	1	1	1
E. coli	single-strain simulated data	60	0	0	1	1	1
	single-strain simulated data + SRR341648	60	0	0	1	1	1
	multiple-strain simulated data	50	0	0	1	1	1
	multiple-strain simulated data + SRR341648	50	0	0	1	1	1

Supplementary Table S11. The TP, TN, FP, recall, and precision of StrainScan on simulated datasets and spiked metagenomic datasets of *P. copri* and *E. coli*.

Species	# of strains	# of clusters	Time used to build the database		
			CST contruction	Sibeliaz	K-mer matrices construction
A. muciniphila	157	53	0.4 h	0.2 h	1.75 h
C. acnes	275	28	0.3 h	0.16 h	0.75 h
P. copri	112	51	0.6 h	0.13 h	0.87 h
E. coli	1433	823	20.4 h	2 h	9.6 h
M. tuberculosis	792	25	1.3 h	17 h	21 h
S. epidermidis	995	378	3 h	1.30 h	2.65 h

Supplementary Table S12. The database construction time of StrainScan for each species. The program is run using 8 threads. The running time of each component depends on the number of strains and their similarity distributions. A large number of strains tend to require more time in CST construction. Highly similar strains (such as *M. tuberculosis*) can generate big clusters and thus require more time for indexing the big clusters. For reference, the memory used to build the *E. coli* (the species with a maximum number of strains) database is 215 GB.

References

[1] E. S. Lander and M. S. Waterman. Genomic mapping by fingerprinting random clones: a mathematical analysis. *Genomics*, 2(3):231–239, 1988.

[2] I. Minkin and P. Medvedev. Scalable multiple whole-genome alignment and locally collinear block construction with SibeliaZ. *Nat Commun*, 11(1):6327, 2020.

[3] M. Scholz, D. V. Ward, E. Pasolli, T. Tolio, M. Zolfo, F. Asnicar, D. T. Truong, A. Tett, A. L. Morrow, and N. Segata. Strain-level microbial epidemiology and population genomics from shotgun metagenomics. *Nat Methods*, 13(5):435–438, 2016.

[4] D. Albanese and C. Donati. Strain profiling and epidemiology of bacterial species from metagenomic sequencing. *Nat Commun*, 8(1):2260, 2017.

[5] T. Vatanen, A. D. Kostic, E. d'Hennezel, H. Siljander, E. A. Franzosa, M. Yassour, R. Kolde, H. Vlamakis, T. D. Arthur, A. Hämäläinen, et al. Variation in microbiome LPS immunogenicity contributes to autoimmunity in humans. *Cell*, 165(6):1551, 2016.

[6] J. Qin, Y. Li, Z. Cai, S. Li, J. Zhu, F. Zhang, S. Liang, W. Zhang, Y. Guan, D. Shen, et al. A metagenome-wide association study of gut microbiota in type 2 diabetes. *Nature*, 490(7418):55–60, 2012.

[7] T. Seemann. Prokka: rapid prokaryotic genome annotation. *Bioinformatics*, 30(14):2068–2069, 2014.

[8] A. J. Page, C. A. Cummins, M. Hunt, V. K. Wong, S. Reuter, M. T. Holden, M. Fookes, D. Falush, J. A. Keane, and J. Parkhill. Roary: rapid large-scale prokaryote pan genome analysis. *Bioinformatics*, 31(22):3691–3693, 2015.

[9] F. De Filippis, E. Pasolli, A. Tett, S. Tarallo, A. Naccarati, M. De Angelis, E. Neviani, L. Cocolin, M. Gobbetti, N. Segata, et al. Distinct genetic and functional traits of human intestinal Prevotella copri strains are associated with different habitual diets. *Cell Host Microbe*, 25(3):444–453, 2019.

[10] G. Marçais, A. L. Delcher, A. M. Phillippy, R. Coston, S. L. Salzberg, and A. Zimin. MUMmer4: A fast and versatile genome alignment system. *PLoS Comput Biol*, 14(1):e1005944, 2018.

[11] M. L. Cummins, C. J. Reid, P. R. Chowdhury, R. N. Bushell, and S. P. Djordjevic. Whole genome sequence analysis of Australian avian pathogenic Escherichia coli that carry the class 1 integrase gene. *Microb Genom*, 5(2), 2019.

[12] C. J. Reid, E. R. Wyrsch, P. R. Chowdhury, T. Zingali, and S. P. Djordjevic. Porcine commensal Escherichia coli: a reservoir for class 1 integrons associated with IS26. *Microb Genom*, 3(12), 2017.

[13] C. Huttenhower, D. Gevers, R. Knight, S. Abubucker, J. H. Badger, A. T. Chinwalla, H. H. Creasy, A. M. Earl, M. G. FitzGerald, R. S. Fulton, et al. Structure, function and diversity of the healthy human microbiome. *Nature*, 486(7402):207–214, 2012.

[14] Lucas R. van D., B. J. Walker, T. J. Straub, C. J. Worby, A. Grote, H. L. Schreiber, C. Anyansi, A. J. Pickering, S. J. Hultgren, A. L. Manson, et al. StrainGE: a toolkit to track and characterize low-abundance strains in complex microbial communities. *Genome Biol*, 23(1):74, 2022.

[15] N. J. Loman, C. Constantinidou, M. Christner, H. Rohde, J. Z. Chan, J. Quick, J. C. Weir, C. Quince, G. P. Smith, J. R. Betley, et al. A culture-independent sequence-based metagenomics approach to the investigation of an outbreak of Shiga-toxigenic Escherichia coli O104:H4. *JAMA*, 309(14):1502–1510, 2013.

[16] J. M. Bryant, S. R. Harris, J. Parkhill, R. Dawson, A. H. Diacon, P. van Helden, A. Pym, A. A. Mahayiddin, C. Chuchottaworn, I. M. Sanne, et al. Whole-genome sequencing to establish relapse or re-infection with Mycobacterium tuberculosis: a retrospective observational study. *Lancet Respir Med*, 1(10):786–792, 2013.

[17] A. Emiola, W. Zhou, and J. Oh. Metagenomic growth rate inferences of strains in situ. *Sci Adv*, 6(17):eaaz2299, 04 2020.

[18] Mária Džunková, Andrés Moya, Xinhua Chen, Ciaran Kelly, and Giuseppe D'Auria. Detection of mixed-strain infections by FACS and ultra-low input genome sequencing. *Gut Microbes*, 11(3):305–309, 2020.

[19] J. Oh, A. L. Byrd, M. Park, H. H. Kong, and J. A. Segre. Temporal stability of the human skin microbiome. *Cell*, 165(4):854–866, 2016.
